# Population Structure in a Comprehensive Genomic Data Set on Human Microsatellite Variation

**DOI:** 10.1534/g3.113.005728

**Published:** 2013-05-01

**Authors:** Trevor J. Pemberton, Michael DeGiorgio, Noah A. Rosenberg

**Affiliations:** * Department of Biology, Stanford University, Stanford, California 94305; † Department of Integrative Biology, University of California, Berkeley, California 94720

**Keywords:** population structure, relatives, short tandem repeats

## Abstract

Over the past two decades, microsatellite genotypes have provided the data for landmark studies of human population-genetic variation. However, the various microsatellite data sets have been prepared with different procedures and sets of markers, so that it has been difficult to synthesize available data for a comprehensive analysis. Here, we combine eight human population-genetic data sets at the 645 microsatellite loci they share in common, accounting for procedural differences in the production of the different data sets, to assemble a single data set containing 5795 individuals from 267 worldwide populations. We perform a systematic analysis of genetic relatedness, detecting 240 intra-population and 92 inter-population pairs of previously unidentified close relatives and proposing standardized subsets of unrelated individuals for use in future studies. We then augment the human data with a data set of 84 chimpanzees at the 246 loci they share in common with the human samples. Multidimensional scaling and neighbor-joining analyses of these data sets offer new insights into the structure of human populations and enable a comparison of genetic variation patterns in chimpanzees with those in humans. Our combined data sets are the largest of their kind reported to date and provide a resource for use in human population-genetic studies.

Since their discovery as an important form of human genetic variation, microsatellites have been central to human evolutionary studies. In a landmark paper, Bowcock *et al.* (1994) reported the first microsatellite study of global human variation, using 30 markers in 148 individuals from 14 indigenous populations, finding that populations cluster by geographic region on a neighbor-joining tree, and that Africans have the highest microsatellite diversity. The Bowcock *et al.* data were used in a variety of subsequent studies (Goldstein *et al.* 1995a,b; Nei and Takezaki 1996; Barbujani *et al.* 1997; Reich and Goldstein 1998; Zhivotovsky *et al.* 2000), and the general findings from these data were refined and confirmed in a series of studies that largely used data sets of comparable size (Jorde *et al.* 1995, 1997; Calafell *et al.* 1998; Jin *et al.* 2000).

The availability of standardized genome-wide marker panels originally designed for linkage analysis (Ghebranious *et al.* 2003) for use in population-genetic samples provided the next major development in studies of human microsatellite variation, increasing the size of data sets from dozens to several hundreds of markers. The first of the larger studies was the worldwide study of Rosenberg *et al.* (2002), who genotyped 377 autosomal markers in 1056 samples from the Human Genome Diversity Project (HGDP-CEPH) cell line panel (Cann *et al.* 2002; Cavalli-Sforza 2005). Partly as a result of its use of a large marker panel, this study uncovered patterns that had not previously been observed. Subsequent studies extended similar approaches to still larger numbers of markers (Ramachandran *et al.* 2005; Rosenberg *et al.* 2005) and additional populations from different regions of the world (Rosenberg *et al.* 2006; Wang *et al.* 2007, 2008; Friedlaender *et al.* 2008; Kopelman *et al.* 2009; Tishkoff *et al.* 2009; Pemberton *et al.* 2012). The data sets from these studies have become widely used in numerous types of analyses (Barnholtz-Sloan *et al.* 2005; Mountain and Ramakrishnan 2005; Amos 2006; Barbujani and Belle 2006; Handley *et al.* 2007; Takezaki and Nei 2008; Romero *et al.* 2009; Hunley and Healy 2011; Ramachandran and Rosenberg 2011; Rosenberg 2011), including tests of new statistical methods (Rosenberg *et al.* 2003; Corander *et al.* 2004; Pfaff *et al.* 2004; Rosenberg 2005; Foll and Gaggiotti 2006; Francois *et al.* 2006; Patterson *et al.* 2006; Cercueil *et al.* 2007; Szpiech *et al.* 2008; DeGiorgio and Rosenberg 2009; Hubisz *et al.* 2009; Shringarpure and Xing 2009; Jombart *et al.* 2010; Fu *et al.* 2011; Gao *et al.* 2011) and evaluations of theoretical results (Rosenberg and Calabrese 2004; Rosenberg and Blum 2007; Rosenberg and Jakobsson 2008; Boca and Rosenberg 2011; DeGiorgio *et al.* 2011; Szpiech and Rosenberg 2011; Reddy and Rosenberg 2012; Tal 2012; Jakobsson *et al.* 2013). They have provided insights into such topics as the worldwide spread of anatomically modern humans (Zhivotovsky *et al.* 2003; Prugnolle *et al.* 2005a; Ray *et al.* 2005; Liu *et al.* 2006; Schroeder *et al.* 2007; DeGiorgio *et al.* 2009; Deshpande *et al.* 2009; Hunley *et al.* 2009; Amos and Hoffman 2010; Ray *et al.* 2010), the relationship of genetic and linguistic variation (Hunley *et al.* 2008, 2012; Lewis 2010; Jay *et al.* 2011; de Filippo *et al.* 2012), and the mechanisms of microsatellite mutation itself (Amos *et al.* 2008; Pemberton *et al.* 2009; Sun *et al.* 2009; Amos 2011). They have been used in host-pathogen evolutionary studies (Prugnolle *et al.* 2005b; Linz *et al.* 2007; Ettinger *et al.* 2009; Ramalho *et al.* 2010), comparisons with anthropometric data (Relethford 2004; Roseman 2004; Manica *et al.* 2007; Nievergelt *et al.* 2007; Weaver *et al.* 2007), and assessments of natural selection (Bamshad and Wooding 2003; Storz *et al.* 2004; Rockman *et al.* 2005; Foll and Gaggiotti 2008; Excoffier *et al.* 2009; Hofer *et al.* 2009), and even in distant fields such as economics (Jellema 2008; Ashraf and Galor 2013).

Most large microsatellite studies since 2005 have merged data with the data set of Rosenberg *et al.* (2002) and its extension (Ramachandran *et al.* 2005; Rosenberg *et al.* 2005) to broaden the set of populations examined (Rosenberg *et al.* 2006; Wang *et al.* 2007, 2008; Friedlaender *et al.* 2008; Kopelman *et al.* 2009; Tishkoff *et al.* 2009). However, these data sets have been prepared with different procedures and sets of markers, and they have therefore been difficult to combine for a comprehensive analysis. A definitive data set that amalgamates all of these data sets offers new opportunities for more complete analyses of patterns of human genetic variation.

Here, we compile the largest modern genome-wide population-genetic data set on human populations assembled to date, in terms of the number of populations investigated. This data set comprises 645 microsatellite loci with genotypes in 5795 individuals from 267 populations. We define subsets of unrelated individuals for use in studies in which relatedness needs to be clearly characterized, and we explore patterns of genetic variation both worldwide and within each of seven major geographic regions. Further, we merge this data set with data for 84 chimpanzees at 246 overlapping loci (Becquet *et al.* 2007), and we investigate relationships between chimpanzee and human genetic variation. Our study yields a resource that can facilitate the use of patterns of human genetic variation in many areas of application.

## Materials and Methods

### Merging of human data sets

We sought to merge eight data sets (Table 1), each comprising individuals genotyped at autosomal microsatellites from the Marshfield Screening Sets (Ghebranious *et al.* 2003). This process presents a challenge for several reasons (Presson *et al.* 2006; Rosenberg *et al.* 2006; Wang *et al.* 2007). First, the screening sets have changed over time, and the different data sets do not have identical sets of markers. Second, the PCR primers used for genotyping have in many cases also changed, so that a locus might appear with systematically different allele sizes in different studies. Third, with or without primer changes, changes in genotype-calling have introduced systematic allele-size changes at some loci. We aim to identify a maximal set of markers found in all studies, accounting for changes in markers, primers, and genotype-calling, so that genotypes from different sources are commensurable.

**Table 1:** Data sets included in the combined data set and their sample sizes

	Sample Size					
Data Set Name	Original Data Set	MS5795*^a^*	MS5547*^b^*	MS5435*^c^*	Obtained From	Reference(s)
HGDP-CEPH	1048	1046	966	947	Rosenberg laboratory	Rosenberg *et al.* 2002, 2005; Ramachandran *et al.* 2005
Native American	436	418	363	338	Rosenberg laboratory	Wang *et al.* 2007
Latino	249	246	244	241	Dataset S1 of	Wang *et al.* 2008
					Wang *et al.* (2008)	
Jewish	80	79	79	77	Rosenberg laboratory	Kopelman *et al.* 2009
Asian Indian	432	430	430	430	Rosenberg laboratory	Rosenberg *et al.* 2006
Chha Gaam Patel (CGP)*^d^*	249 (203)	203	185	180	Rosenberg laboratory	Pemberton *et al.* 2012
Pacific Islander	936	847	756	709	F. Friedlaender and	Friedlaender *et al.* 2008
					J. Friedlaender	
African	2561	2526	2524	2513	Supplement of Tishkoff *et al.* (2009)	Tishkoff *et al.* 2009
		**MS5879***^a^*	**MS5631***^b^*	**MS5519***^c^*		
Chimpanzee	84	84	84	84	Dataset S1 of	Becquet *et al.* 2007
					Becquet *et al.* (2007)	

a MS5795 and MS5879 represent the complete combined-human and combined-human–chimpanzee data sets, respectively, and they include intra-population relative pairs.

b MS5547 and MS5631 are constructed from MS5795 and MS5879, respectively, by the removal of a member of every intra-population first-degree relative pair (Table S21).

c MS5435 and MS5519 are constructed from MS5547 and MS5631, respectively, by the removal of a member of every intra-population second-degree relative pair (Table S22).

d Some Gujarati individuals were included in both the Rosenberg *et al.* (2006) and Pemberton *et al.* (2012) studies. The number of unique individuals included from the CGP data set is given in parentheses.

**Figure 1:**
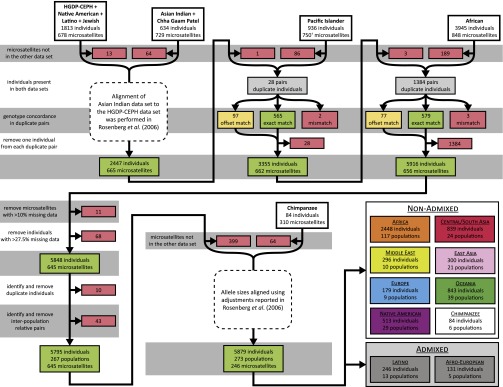
Data filtering steps used to prepare the combined data sets. Steps are shown in the order in which they were applied. Loci removed in one step were not subsequently considered. The numbers of loci or individuals removed are shown in red shaded boxes, numbers of loci whose genotypes were adjusted by a common size difference are shown in yellow shaded boxes (offset match), and numbers of loci whose genotypes matched in duplicate individuals are shown in green shaded boxes (exact match). The numbers of loci and individuals in the combined data set after each merging are shown in green shaded boxes. Sample sizes for each geographic region appear in Table 2. Key: ^†^Two of the 751 loci in the initial Pacific Islander data set (ATA27A06N and ATA27A06P) genotype the same locus; ATA27A06P was included in the combined data set.

The Rosenberg *et al.* (2002) study of 1056 individuals from the HGDP-CEPH panel was the first study to utilize a Marshfield Screening Set for population genetics, genotyping 377 autosomal microsatellites in Marshfield Screening Set 10. These data were later augmented by 406 additional loci from Marshfield Screening Sets 13 and 52, producing, after small changes to the set of individuals, a collection of 1048 individuals at 783 loci (Ramachandran *et al.* 2005; Rosenberg *et al.* 2005). Several studies then used overlapping marker collections to perform similar investigations in other populations. Three studies concurrently genotyped their samples for 751 autosomal microsatellites in Marshfield Screening Sets 16 and 54: Wang *et al.* (2007) studied 436 individuals from 24 Native American populations and one Siberian population (Native American data set henceforth), Wang *et al.* (2008) studied 249 individuals from 13 Latin American Mestizo populations (Latino data set), and Kopelman *et al.* (2009) studied 80 individuals from four Jewish populations (Jewish data set). In the Native American study, all three data sets were merged with the HGDP-CEPH data (Ramachandran *et al.* 2005; Rosenberg *et al.* 2005), only considering markers shared among data sets and adjusting for allele-size differences introduced by primer changes, so that alleles in the newer genotypes matched those in the HGDP-CEPH data set (Wang *et al.* 2007). We used the combined HGDP-CEPH, Native American, Latino, and Jewish data set of 1813 individuals and 678 loci as the starting point for producing our combined data set (Figure 1).

#### Asian Indians:

Rosenberg *et al.* (2006) studied 432 individuals from 15 Asian Indian populations (Asian Indian data set), and Pemberton *et al.* (2012) studied an overlapping set of 249 individuals from the Gujarati population, one of the populations of Rosenberg *et al.* (Chha Gaam Patel or CGP data set). The two studies performed genotyping and data preparation concurrently for 729 autosomal microsatellites in Marshfield Screening Sets 13 and 52. We merged the 634 distinct individuals from the Asian Indian and CGP data sets with the combined HGDP-CEPH, Native American, Latino, and Jewish data set at the 665 loci that these data sets shared in common (Figure 1). Rosenberg *et al.* had previously adjusted the genotypes in the Asian Indian and CGP data to match the HGDP-CEPH data set (Rosenberg *et al.* 2006); consequently, the adjusted Asian Indian and CGP data sets could simply be concatenated with the combined HGDP-CEPH, Native American, Latino, and Jewish data set, without any need for additional genotypic adjustments.

#### Pacific Islanders:

Friedlaender *et al.* (2008) studied 936 individuals from 38 Pacific Islander and two Taiwanese populations using 751 autosomal microsatellites in Marshfield Screening Sets 16 and 54 (Pacific Islander data set). We merged these data with the combined HGDP-CEPH, Native American, Latino, Jewish, Asian Indian and CGP data set at the 664 loci shared by the Pacific Islander data set (Figure 1). We found that two loci in the Pacific Islander data set (ATA27A06N and ATA27A06P) genotyped the same locus using different primer pairs, with ATA27A06P having genotypes 7 nucleotides (nt) longer than those of ATA27A06N. We chose ATA27A06P at random for inclusion in the combined data set. To ensure that each individual and each population had a unique identifier in the combined data set, we added 1000 and 2000, respectively, to population and individual identifiers in the Pacific Islander data set.

Some individuals in the Pacific Islander data set had been previously genotyped as part of the HGDP-CEPH Melanesian and Papuan populations (Friedlaender *et al.* 2008). We therefore determined the proportions of loci at which a pair of individuals shared 0, 1, and 2 alleles identical by state (IBS)—denoted *p*_0_, *p*_1_, and *p*_2_, respectively—for each pair of individuals, one from the Pacific Islander data set and the other from the HGDP-CEPH Melanesians and Papuans, and using in the calculation for a given pair only those loci for which neither individual was missing genotypes. We identified twenty-eight pairs with *p*_2_ > 0.831 as putative duplicate pairs; all other pairs had *p*_2_ < 0.460. Nine of the putative duplicate pairs involved the HGDP-CEPH Papuan and Pacific Islander East Highlands (Gimi & Goroka) populations, and the remaining 19 pairs linked the HGDP-CEPH Melanesian and Pacific Islander Nasioi populations.

To identify loci at which a systematic change in allele size exists between the Pacific Islander data set and the combined HGDP-CEPH, Native American, Latino, Jewish, Asian Indian, and CGP data set, separately for each locus, we translated the allele sizes of the Pacific Islander data set by a constant *c*, and we computed the proportion of duplicate pairs that shared 2 alleles IBS (*g*_c,2_). For a given locus, denoting the smallest and largest alleles among individuals from the combined data set by *a* and *A*, respectively, and the smallest and largest alleles in the Pacific Islander data set by *b* and *B*, respectively, we considered all possible integer translation constants in the range [*a*–*B*,*A*–*b*]. The constant that maximized *g*_c,2_, considering only duplicate pairs for which both individuals had non-missing genotypes, was labeled c*. For this calculation, all loci had non-missing genotypes for at least 15 of 28 duplicate pairs.

Of the 664 loci considered, with the optimal constant of translation applied, 662 had a close match of the translated Pacific Islander genotypes to the HGDP-CEPH genotypes, with *g*_c*,2_ > 0.773. The other two loci, ATAC026 and ATA84D02, had *g*_c*,2_ < 0.519, and we excluded them from the combined data set on the grounds of an inability to determine the shift in allele sizes (Figure 1, “mismatch” loci).

For the 662 remaining loci, to further validate the inferred values of c*, we performed additional analyses of agreement between duplicate pairs. Among these loci, 527 had *g*_c*,2_ = 1 and 629 had *g*_c*,2_ ≥ 0.950. Considering *g*_c,1˅2_, the proportion of duplicate pairs with non-missing genotypes that shared 1 or 2 alleles IBS when using translation constant *c*, all 662 loci had *g*_c*,1˅2_ > 0.954, and 655 had *g*_c*,1˅2_ = 1. Of 135 loci with 0.773 < *g*_c*,2_ < 1, one locus had a single pair with no matching alleles, while the other 134 had at least one pair that shared only 1 allele IBS; 106 loci had only a single pair, while the remaining 28 had at most 4 pairs. Because mismatches could be plausibly explained by systematic allele-size translations with a small amount of genotyping error, we retained all 662 loci. Among these loci, 565 had c* = 0 (Figure 1, “exact match” loci); at the other 97 (Figure 1, “offset match” loci), we adjusted allele sizes in the Pacific Islander data set by the appropriate c* (Supporting Information, Table S1).

We note that in their study, Friedlaender *et al.* had also performed adjustments, adjusting HGDP-CEPH genotypes to match the Pacific Islander data set. At 92 of our 97 offset match loci, our genotype adjustments and those of Friedlaender *et al.* agreed (Table S1). For loci D13S796, D3S1744, and D8S1477, our adjustment was 1 nt longer than that used by Friedlaender *et al.*; however, after applying our adjustment, all three loci have *g*_c*,2_ = 1, and we therefore regarded our adjustment as likely to be accurate. For locus D5S1725, our adjustment exceeds that of Friedlaender *et al.* by 4 nt, but it is identical to the adjustment of Rosenberg *et al.* (2006) when aligning the Asian Indian data set to the HGDP-CEPH data set. For locus D18S1376, we apply an adjustment, but Friedlaender *et al.* did not; our adjustment again matches that used by Rosenberg *et al.* (2006). For loci D5S1725 and D18S1376, the same DNA primer pairs were used by Friedlaender *et al.* and Rosenberg *et al.*; consequently, we regarded the c* adjustment here as likely to be accurate. The five discrepancies between adjustments determined here and those reported by Friedlaender *et al.* likely reflect either typographical errors in the adjustment table of Friedlaender *et al.* or incorrect adjustments applied by Friedlaender *et al.* in their combined data set.

After adjusting the 97 offset-match loci, we recalculated *p*_2_ for all 28 duplicate pairs, using all 662 remaining loci; each pair had *p*_2_ > 0.974. From each pair, we excluded from the combined data set the individual from the Pacific Islander data set, leaving 3355 individuals (Figure 1). Because the HGDP-CEPH Melanesians and Papuans were contributed to the HGDP-CEPH panel from the Pacific Islander Nasioi and East Highlands (Gimi & Goroka) populations, respectively (Friedlaender *et al.* 2008), we merged the HGDP-CEPH Melanesian and Pacific Islander Nasioi samples and the HGDP-CEPH Papuan and Pacific Islander East Highlands samples, retaining the labels “Nasioi” and “East Highlands.”

#### Africans:

Tishkoff *et al.* (2009) studied genotypes of 2561 individuals from 112 African populations, five populations with admixed African and European ancestry (henceforth Afro-Europeans), one Native Australian population, and one Yemenite population at 848 microsatellites in Marshfield Screening Sets 16 and 54 (African data set). We merged these data with the combined HGDP-CEPH, Native American, Latino, Jewish, Asian Indian, CGP, and Pacific Islander data set at the 659 loci that the African data set shared. To ensure that each individual and each population had a unique identifier in the combined data set, we added 1100 and 70,000, respectively, to all population and individual identifiers in the African data set.

The African data set was provided in the Tishkoff *et al.* (2009) online supplement already merged with the HGDP-CEPH (Ramachandran *et al.* 2005; Rosenberg *et al.* 2005) and Asian Indian (Rosenberg *et al.* 2006) data sets. Tishkoff *et al.* had adjusted genotypes in the HGDP-CEPH and Asian Indian data sets to match the African data set, whereas we aim to adjust their African genotypes to match the combined HGDP-CEPH, Native American, Latino, Jewish, Asian Indian, CGP, and Pacific Islander data set. To identify loci at which a systematic change in allele size exists between the African data set and the combined data set, we applied the same procedure used for the Pacific Islander data set, considering at each locus only those pairs among the 1384 duplicate pairs (952 HGDP-CEPH and 432 Asian Indian) for which both individuals had non-missing genotypes; all loci had at least 960 of the 1384 duplicate pairs with non-missing genotypes.

Among the 659 loci, with the optimal constant of translation applied, 656 had *g*_c*,2_ = 1, indicating perfect agreement, and the other three had *g*_c*,2_ < 0.990 (Figure 1, “mismatch”). At one of these three loci (D21S1411), the 421 Asian Indian duplicate pairs with non-missing genotypes had an optimal shift that differed from that of the 899 HGDP-CEPH duplicate pairs with non-missing genotypes; we hypothesize that Tishkoff *et al.* separately merged the HGDP-CEPH and Asian Indian data sets with their own new genotypes, and that the translation was applied differently to the two data sets at this locus. We excluded this locus from the combined data set on the grounds of an inability to determine the shift in allele sizes. At the other two loci (TAGA031Z and GATA8H05), Tishkoff *et al.* had applied size adjustments only to specific alleles, introducing mismatches in a small number of duplicate pairs; we excluded both TAGA031Z and GATA8H05 from our combined data set. Of the 659 loci that the African data set shared in common with the combined data set, these were the only two loci for which Tishkoff *et al.* had performed allele-specific adjustments. Among the 656 loci with *g*_c*,2_ = 1, 579 had c* = 0 (Figure 1, “exact match” loci); at the other 77 (Figure 1, “offset match” loci), we adjusted allele sizes in the African data set by the inferred c* to align them with those in the combined data set (Table S2).

Our genotype adjustments and those used by Tishkoff *et al.* agreed at 75 of the 77 offset match loci (Table S2). For locus D5S1725, our adjustment exceeds that of Tishkoff *et al.* by 4 nt, but it is identical to the adjustment used by Rosenberg *et al.* (2006) when aligning the Asian Indian data set to the HGDP-CEPH data set. For locus D18S1376, we applied an adjustment, but Tishkoff *et al.* did not; our adjustment matches that of Rosenberg *et al.* (2006). For both of these loci, Tishkoff *et al.* and Rosenberg *et al.* used the same primer pair; consequently, we regard the c* adjustment determined here as likely to be accurate. The two differences between adjustments determined here and those reported by Tishkoff *et al.* (2009) likely reflect discrepancies between their genotype data file and their adjustment table, and not incorrect adjustments in the data used in their study; unlike for the Pacific Islander data set, for which we inferred c* values from the Pacific Islander genotypes prior to merging, for the African data set, we inferred c* from merged genotypes that were actually analyzed in the study by Tishkoff *et al.*

Following the genotypic adjustment for the 77 offset-match loci, we recalculated *p*_2_ for all 1384 HGDP-CEPH and Asian Indian duplicate pairs; all pairs had *p*_2_ = 1. From each pair, we excluded the HGDP-CEPH or Asian Indian individual from the African data set, leaving 5916 individuals in total (Figure 1).

#### A note on the merging order:

While we chose to merge all non-HGDP-CEPH data sets with the HGDP-CEPH data set—aligning allele sizes to the HGDP-CEPH—changing the merging order or the data set to which alleles are aligned is unlikely to substantially alter the patterns observed in subsequent analyses. The high degree of concordance between our allele size adjustments and those applied in previous studies suggests that at only a small number of loci does the potential exist for small allele size discrepancies to occur between the data set reported here and data sets that might be obtained with alternative merging strategies; consequently, patterns in allele size differences across populations would remain almost entirely unchanged.

### Missing data

In the combined HGDP-CEPH, Native American, Latino, Jewish, Asian Indian, CGP, Pacific Islander, and African data set of 5916 individuals (combined human data set henceforth), separately for each locus, we computed the fraction of individuals whose genotypes were missing (*l*_m_). We removed 11 loci with *l*_m_ > 0.146 (Table S3); all other loci had *l*_m_ < 0.086 (mean 0.034, standard deviation [SD] 0.011).

Next, separately for each individual, we determined the fraction *i*_m_ of missing genotypes among the 645 remaining loci. We removed 68 individuals with *i_m_* > 0.277 (Table S4); all other individuals had *i*_m_ < 0.275 (mean 0.059, SD 0.056). This threshold ensured that all pairs of individuals in the combined human data set shared non-missing genotypes at more than half of the loci.

Following the exclusion of these 11 loci and 68 individuals, the combined human data set comprised 5848 individuals from 267 worldwide populations, with genotypes at 645 microsatellite loci (Figure 1).

### Relative pairs

We identified three types of pairs of duplicate or related individuals in the combined human data set. First, in merging data sets, we have already identified and removed individuals duplicated between data sets. Second, because during sample collection, individuals from the same family might have been included in a population sample, we identified intra-population relative pairs in each population. Third, as relative pairs might also exist across population samples, owing to labeling error or sample collection from neighboring populations, we identified inter-population relative pairs within each geographic region.

We identified pairs of individuals who were related more closely than first-cousins, following the methods of Rosenberg (2006) using identity-by-state allele sharing (*p*_0_, *p*_1_, and *p*_2_) and the likelihood approach of RELPAIR (version 2.0.1) (Boehnke and Cox 1997; Epstein *et al.* 2000). RELPAIR assesses likelihoods of eight relationship types: monozygotic-twin (MZ), full-sibling (FS), parent-offspring (PO), half-sibling (HS), grandparent-grandchild (GG), avuncular (AV), first-cousin (CO), and unrelated (UN). We disregard CO inferences, as they are less reliable than inferences for closer relationships (Boehnke and Cox 1997; Epstein *et al.* 2000; Pemberton *et al.* 2010). RELPAIR sometimes has difficulty distinguishing among types of second-degree relative pairs (AV, GG, HS). Here, we regard second-degree inferences as correct and report the most likely inference. In all RELPAIR analyses, we set the critical value to 100 and the genotyping error rate to 0.008 (Rosenberg 2006). Physical positions of 628 of the 645 microsatellite loci were available from Pemberton *et al.* (2009), and we were able to interpolate the genetic map position for 612 of these 628 loci on the Rutgers combined physical-linkage map (http://compgen.rutgers.edu/mapinterpolator) (Kong *et al.* 2004; Matise *et al.* 2007); we restricted RELPAIR analyses to these 612 loci.

#### Intra-population relative pairs:

To exclude intra-population pairs of close relatives from the combined human data set, separately in each population, we applied RELPAIR using count estimates of allele frequencies in that population. In these analyses, we disregarded the HGDP-CEPH Karitiana and Surui populations, as it has been noted that it is particularly difficult to reliably infer relative pairs in these populations (Rosenberg 2006).

Intra-population relative pairs had previously been identified in the HGDP-CEPH (Rosenberg 2006), Native American (Wang *et al.* 2007), Asian Indian (Rosenberg *et al.* 2006), CGP (Pemberton *et al.* 2012), and Jewish (Kopelman *et al.* 2009) data sets; we found no additional intra-population pairs in these data sets beyond those reported previously.

Intra-population relative pairs had also been previously identified in the African data set (Tishkoff *et al.* 2009); however, separately considering the 119 populations from the African data set that are present in the combined data set, we identified 14 previously unreported intra-population pairs (Figure S1): 2 first-degree pairs (Table S5; 1 PO, 1 FS), and 12 second-degree pairs (Table S6; 1 AV, 5 GG, 6 HS).

Neither the Latino (Wang *et al.* 2008) nor the Pacific Islander (Friedlaender *et al.* 2008) data sets had been previously checked for intra-population relative pairs. We identified 6 intra-population relative pairs among the Latino populations in the combined data set (Figure S2): 2 first-degree (Table S7; 1 PO, 1 FS) and 4 second-degree pairs (Table S8; 2 AV, 2 HS). In the populations from the Pacific Islander data set present in the combined human data set, we identified 220 relative pairs (Figure S3, Figure S4, and Figure S5): 6 MZ pairs (Table S9), 127 first-degree pairs (Table S10; 56 PO, 71 FS), and 87 second-degree pairs (Table S11; 37 AV, 25 GG, 25 HS). The 56 intra-population PO pairs include 13 parent/parent/offspring trios (Table S12).

#### Inter-population relative pairs:

To exclude inter-population pairs of close relatives from the combined human data set, separately on subsets of individuals from each of seven geographic regions (Africa, the Middle East, Europe, Central/South Asia, East Asia, Oceania, and the Americas), we applied RELPAIR using count estimates of allele frequencies in the region, and considered only pairs of individuals from distinct populations. We also applied RELPAIR on the pooled set of five Afro-European populations, using count estimates of allele frequencies in these individuals. We included Latino individuals in the Americas analysis, as concurrent genotyping of the Native American and Latino data sets could have generated opportunities for sample mislabeling and therefore, for unexpected inter-population relationships.

Inter-population relative pairs have been previously identified in the HGDP-CEPH (Rosenberg 2006), Asian Indian (Rosenberg *et al.* 2006), CGP (Pemberton *et al.* 2012), and Jewish (Kopelman *et al.* 2009) data sets; consistent with these analyses, we found no inter-population relative pairs involving these data sets. Further, we found no such pairs in our analyses of the Middle East, Europe, Central/South Asia, and East Asia, or in the Afro-Europeans (Figure S6).

However, among the 2450 African individuals in the combined human data set, we identified two inter-population pairs of individuals (Figure S6): 1 first-degree PO pair (Table S13) and 1 second-degree HS pair (Table S14). Both pairs involve individuals from the African data set, and neither was reported by Tishkoff *et al.* (2009) in their analysis of inter-population relative pairs.

Among the 894 Oceanian individuals in the combined human data set, we identified 80 inter-population relative pairs (Figure S6): 2 MZ pairs (Table S15), 24 first-degree pairs (Table S16; 12 PO, 12 FS), and 54 second-degree pairs (Table S17; 46 AV, 4 GG, 4 HS). Together with the 56 intra-population PO pairs we identified (Table S12), the inter-population PO pairs contribute to 3 additional trios (Table S18).

Finally, among the 759 individuals from the Americas present in the combined human data set (513 Native Americans, 246 Latinos), we identified ten inter-population relative pairs (Figure S6), all of which were AV pairs (Table S19). All ten pairs involve individuals from the Native American data set.

#### Standardized subsets of individuals:

In our comprehensive evaluation of relatedness among the 5848 individuals in the combined human data set, we identified 332 previously unreported relative pairs (Tables S5–S19 in Supporting Information). Incorporating information on relative pairs previously reported in similar analyses of some of its constituent data sets (Rosenberg 2006; Wang *et al.* 2007; Kopelman *et al.* 2009; Pemberton *et al.* 2012), we next created three standard sets of individuals: (1) a set with no MZ pairs or first-degree inter-population relatives, (2) a set with no MZ pairs, first-degree inter-population relatives, or intra-population first-degree relatives, and (3) a set with no MZ pairs, first-degree inter-population relatives, or intra-population first- or second-degree relatives.

The production of these subsets followed a similar procedure to that of Rosenberg (2006). First, we removed those individuals excluded from the recommended subsets of the HGDP-CEPH (Rosenberg 2006), CGP (Pemberton *et al.* 2012), Jewish (Kopelman *et al.* 2009), and Native American (Wang *et al.* 2007) data sets. We removed one member from each intra-population pair identified here (Tables S5–S11 in Supporting Information) and both individuals from each of the 2 MZ (Table S15) and 25 first-degree inter-population relative pairs (Table S13 and Table S16), as the correct population affiliation was unknown. Because RELPAIR can erroneously report relative pairs in structured populations, identifying unrelated individuals from the same or similar populations as relatives, our inter-population second-degree inferences are less reliable than for closer relationships. We therefore did not exclude members of the 65 inter-population second-degree relative pairs (Table S14, Table S17, and Table S19). To minimize the number of individuals removed, we preferentially omitted individuals present in two or more relative pairs (either intra- or inter-population). In situations where either individual in a relative pair could be removed, we removed the individual with the higher level of missing data.

While the 8 MZ pairs we identified in the Pacific Islander data set might indeed represent twins (Table S9 and Table S15), it is perhaps more likely that they are pairs of duplicate samples. Following the exclusion of 53 individuals from MZ and inter-population first-degree relative pairs (some of whom appeared in more than one such pair), the combined human data set contained 5795 individuals from 267 populations (Table S20; mean sample size 21.7, SD 16.8, minimum 3) with genotypes at 645 loci (Figure 1; subset MS5795 henceforth).

Next, by removing from MS5795 a member of every intra-population first-degree relative pair, we created subset MS5547, a set of 5547 individuals (Table S21). Finally, by the additional removal from MS5547 of a member of every intra-population second-degree relative pair, we created subset MS5435, consisting of 5435 individuals (Table S22).

In MS5795, the sample size for Gujaratis is 252 individuals, much greater than for the other populations (Table S20; maximum 61, mean 20.8, SD 9.1). Rosenberg *et al.* had used a subset of 50 Gujaratis to make the Gujarati sample size similar to those of other populations (Rosenberg *et al.* 2006). We therefore propose three further subsets that restrict the Gujarati population to only the 49 individuals studied by Rosenberg *et al.* that appear in MS5795; the restrictions of MS5795, MS5547, and MS5435 generate data sets MS5592, MS5362, and MS5255, respectively.

### Geographic coordinates

We obtained geographic coordinates for 258 of the 267 populations in the combined human data set (Table S20), taking population locations for the HGDP-CEPH data set from Rosenberg (2011), for the Native American data set from Wang *et al.* (2007), for the Latino data set from Wang *et al.* (2008), for the Asian Indian data set from Rosenberg *et al.* (2006), for the Pacific Islander data set from Françoise Friedlaender (personal communication), and for the African data set from Tishkoff *et al.* (2009), where available.

For four of the five Afro-European populations and the one Australian population in the African data set, Tishkoff *et al.* did not provide geographic coordinates. For three of the Afro-European populations, we provide the coordinates of the city where sampling took place—Baltimore, Chicago, and Pittsburgh. The fourth Afro-European population included samples from across North Carolina and we do not provide coordinates. No sampling location was available for the Australian population. For the four populations in the Jewish data set, we report the coordinates of Ashkelon, Israel, where sampling took place (Kopelman *et al.* 2009).

### Chimpanzee data

Becquet *et al.* (2007) had studied 84 chimpanzees—78 common chimpanzees and six bonobos—from six groups, using genotypes at 310 microsatellite loci (Table S23; chimpanzee data set henceforth). We merged these data with the MS5795 human data set at the 246 loci shared by the chimpanzee data set (Figure 1; combined human–chimpanzee data set henceforth).

The chimpanzee data set had been genotyped in 2005 for a panel of microsatellites that included Marshfield Screening Set 13 (Becquet *et al.* 2007). The Asian Indian data set was also genotyped for this screening set in 2004, by the same group that genotyped the chimpanzees (Marshfield Clinic, Marshfield, WI). It is therefore likely that primer pairs and genotype-calling procedures for both data sets were identical; consequently, we used the size adjustments applied by Rosenberg *et al.* (2006) to align the Asian Indian data set to the HGDP-CEPH data set for aligning the chimpanzee data set to the combined human data set.

In the combined human–chimpanzee data set, we calculated *l*_m_ for each locus and *i*_m_ for each individual. All loci have *l*_m_ < 0.065 (mean 0.030, SD 0.010). We retained three human individuals with *i*_m_ > 0.275 (Table S24; maximum = 0.382) in the combined human–chimpanzee data set, to make its analyses directly comparable to those for the combined human data set; all other humans and chimpanzees had *i_m_* < 0.269 (mean 0.030, SD 0.030). Both intra- and inter-population relative pairs have been previously identified in the chimpanzee data set (Becquet *et al.* 2007); identity-by-state allele sharing among the 84 chimpanzees in the combined human–chimpanzee data set did not suggest the presence of additional relative pairs (Figure S7). Consequently, adding all 84 chimpanzees to MS5795, MS5592, MS5547, MS5362, MS5435, and MS5255 generates data sets MS5879, MS5676, MS5631, MS5446, MS5519, and MS5339, respectively (Table 2). The combined human–chimpanzee data set contains 5879 individuals from 267 human and six chimpanzee populations, with genotypes at 246 loci (Figure 1).

### Population level per-locus missing data

In each of the three subsets of the combined human data set of 645 loci (MS5795, MS5547, and MS5435) and each of the three subsets of the combined human–chimpanzee data set of 246 loci (MS5879, MS5631, and MS5519), we identified loci with no genotype data in at least one population. For the human data sets, we identified 27 such loci (Table S25); we identified 10 such loci in the human–chimpanzee data sets (Table S26). We retained these loci in our combined data sets and population genetic analyses; however, for other analyses, especially at the population level, it is not unreasonable to exclude them.

### Population genetic analyses

#### Multidimensional scaling:

To search for individual labeling errors and to show that the genotypes have been properly aligned across data sets, we performed classical metric multidimensional scaling (MDS). If the population of an individual was mislabeled, we would expect the individual not to cluster genetically with other individuals sharing the same label. Similarly, if data sets were misaligned, then individuals would cluster by data set of origin; this pattern would be most evident for populations sampled in multiple data sets (*e.g.*, separate Yoruba samples in the HGDP-CEPH and African data sets). MDS analysis, both of the whole data set and of various subsets, can then reveal the likely presence of mislabeling or misalignment.

We constructed an allele-sharing distance matrix for all pairs of individuals in the MS5435 human data set, using in the calculation for a given pair only those loci for which neither individual was missing genotypes. We applied MDS on this distance matrix using the *cmdscale* command in R (version 2.15.1; R Development Core Team 2011). We also performed separate MDS analyses on each geographic region, using subsets of the matrix containing only those individuals with membership in a given population subset. In addition, we performed separate MDS analyses on subsets of the matrix in which each geographic region was represented by the same number of individuals (randomly sampled without replacement). For these analyses, we did not consider population membership when sampling the individuals; consequently, not all populations are necessarily represented.

To confirm the alignment of the data sets, we compared locations in the MDS plot of individuals from the Native American, Latino, Jewish, Asian Indian, CGP, Pacific Islander, and African data sets with those of the HGDP-CEPH individuals. We performed each comparison both in a worldwide MDS plot, as well as in separate MDS analyses restricted to particular subsets of individuals. First, following Behar *et al.* (2010), we used *kernelUD* from the *adehabitatHR* package (Calenge 2006) in R to estimate the utilization distribution of the MDS plot by HGDP-CEPH individuals from each geographic region, and we plotted the reported contour containing 92% of the distribution, as smoothed using the least-square cross-validation option. To investigate the alignment of the Pacific Islander and HGDP-CEPH data sets, we used the same approach to estimate the 92% contour for the 17 HGDP-CEPH East Highlands individuals, and then plotted this range alongside the coordinates of the 10 East Highlands individuals in the Pacific Islander data set. For the alignment of the African and HGDP-CEPH data sets, we similarly compared locations of the 22 HGDP-CEPH Yoruba individuals with those of the 25 Yoruba individuals in the African data set. As no overlapping HGDP-CEPH populations were available for the other data sets, we instead plotted the individuals from these data sets and compared the locations of (1) individuals from the Native American data set and the HGDP-CEPH Native American populations, (2) individuals from the Latino data set and the HGDP-CEPH European and Native American populations, (3) individuals from the Jewish data set and the HGDP-CEPH Middle Eastern and European populations, and (4) individuals from the Asian Indian and CGP data sets and the HGDP-CEPH Central/South Asian populations.

To investigate the similarity of MDS plots to the geographic locations of sampled individuals, we used the Procrustes approach (Wang *et al.* 2010). Assigning individuals from 244 non-admixed, non-Jewish populations the coordinates of their populations (Table S20), we computed the Procrustes similarity, *t_0_*, and rotation angle, θ, between the Gall-Peters projection of their (longitude, latitude) coordinates to their MDS (dimension 1, dimension 2) coordinates. We evaluated the significance of *t_0_* under the null hypothesis of no similarity between geographic and MDS locations using 10,000 permutations of population labels, each retaining a shared label for all individuals from the same population. We performed separate Procrustes comparisons of genetic and Gall-Peters-projected geographic coordinates for various subsets of MS5435, producing MDS plots by rotating individual MDS coordinates by angle θ about the centroid.

#### Neighbor-joining:

Using *microsat* (Minch *et al.* 1998), we evaluated population-level pairwise allele-sharing distance (one minus the proportion of shared alleles), using all 246 loci in the MS5519 set. For a population pair, loci for which one or both populations had no data were ignored in the calculation. We constructed a greedy-consensus (Bryant 2003) neighbor-joining tree (Saitou and Nei 1987) using the *neighbor* and *consensus* programs in the *phylip* package (Felsenstein 2008) from 1000 bootstrap resamples across loci, and we visualized the tree with Dendroscope (version 3) (Huson and Scornavacca 2012).

#### Heterozygosity:

We evaluated mean expected heterozygosity across the 645 loci in the MS5795 human data set and across the 246 loci in the MS5879 human–chimpanzee data set. We calculated per-locus estimates accounting for the presence of close relatives (DeGiorgio and Rosenberg 2009), treating RELPAIR inferences (Tables S5–S11 in Supporting Information) as accurate. At a given locus, we considered only non-missing genotypes and corrected for only those relative pairs for which both individuals had non-missing genotypes. In 13 human populations and 1 chimpanzee population, one or more loci at which all individuals had missing data were omitted from the calculation.

To evaluate the relationship between expected heterozygosity and distance from Africa, for each of the 239 non-admixed, non-Jewish populations with geographic coordinates available and a sample size of five or more individuals, we calculated distance from Addis Ababa, Ethiopia (9°N, 38°E) along waypoint routes (Ramachandran *et al.* 2005) with *rdist.earth* from the *fields* package in R, using 6371 km for the radius of the earth. The Cairo waypoint was used for all populations except Sub-Saharan African populations and the Beja and Mozabite populations. Istanbul was used for all populations classified as European, other than the Adygei and Russian populations. Phnom Penh was used for Oceanian populations, and Anadyr and Prince Rupert were used for Native American populations. We used *lm* in R to compute the coefficient of determination (*R^2^*) for the regression of expected heterozygosity on geographic distance.

## Results and Discussion

**Figure 2:**
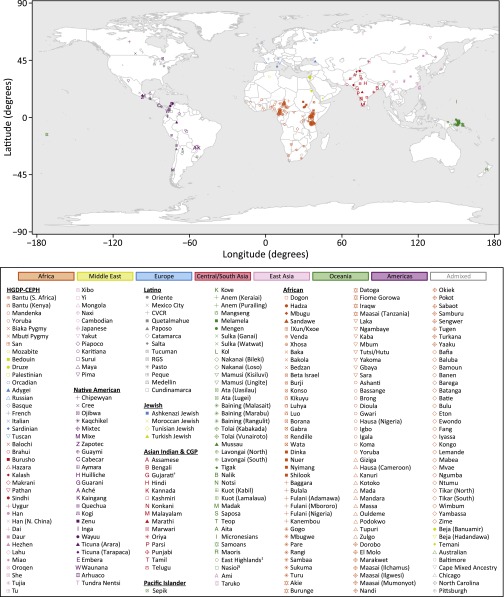
Equirectangular projection of the geographic coordinates of 265 populations in the combined human data set. Two populations without geographic coordinates (Australian, North Carolina) are not shown. Geographic coordinates appear in Table S20. African populations were assigned the same symbol if they had similar cluster memberships in the *K* = 14 *Structure* analysis of Tishkoff *et al.* (2009). Pacific Islander populations from the same tribe were assigned the same symbol. Key: ^†^This population includes the CGP Gujarati individuals studied by Pemberton *et al.* (2012); ^‡^This population subsumes the HGDP-CEPH Papuan population; ^§^This population subsumes the HGDP-CEPH Melanesian population.

We have integrated eight published human microsatellite genotype data sets to create a comprehensive data set of 5795 individuals representing 267 worldwide human populations (Figure 2), with genotypes at 645 loci. This data set provides the largest data resource assembled to date for studies of microsatellite variation, and it contains the most populations of any modern genome-wide population genetic data set.

### Data validation

**Figure 3:**
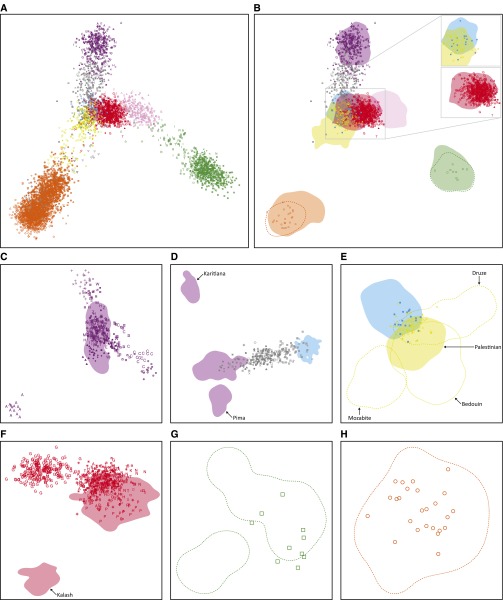
Procrustes-transformed multidimensional scaling (MDS) representations of pairwise allele-sharing distances between individuals. (A) MDS plot of all individuals in the MS5435 data set, colored by geographic affiliation and indicated by the symbols defined in Figure 2. (B) MDS locations of selected individuals from the non-HGDP-CEPH data sets overlaid on utilization distributions for the HGDP-CEPH data set. The figure is a different graphical representation of the MDS coordinates in A. Inset, the Jewish data set in relation to the HGDP-CEPH Middle Eastern and European samples (top), and the Asian Indian and CGP data sets in relation to the HGDP-CEPH Central/South Asian samples (bottom). (C) MDS plot of 325 Native American individuals in the Native American data set and 64 HGDP-CEPH Native American individuals. (D) MDS plot of 241 individuals in the Latino data set and 64 HGDP-CEPH Native American and 158 HGDP-CEPH European individuals. (E) MDS plot of 77 individuals in the Jewish data set and 158 HGDP-CEPH European and 163 HGDP-CEPH Middle Eastern individuals. (F) MDS plot of 610 Asian Indian individuals in the Asian Indian and CGP data sets and 200 HGDP-CEPH Central/South Asian individuals. All HGDP-CEPH Kalash samples lie in the bottom-left shaded area; all other HGDP-CEPH Central/South Asian samples lie in the top-right shaded area. (G) MDS plot of 10 East Highlands individuals in the Pacific Islander data set and 17 HGDP-CEPH East Highlands individuals. The bottom-left contour contains four HGDP-CEPH individuals (540, 545, 546, and 547); all other HGDP-CEPH individuals lie in the top-right contour. (H) MDS plot of 25 Yoruba individuals in the African data set and 22 HGDP-CEPH Yoruba individuals. (B–H) Colored areas represent HGDP-CEPH utilization distribution ranges for full geographic regions, with the exception that the yellow shaded area in E represents the distribution range of 46 HGDP-CEPH Palestinian individuals. The dashed orange (B, H), green (B, G), and yellow (E) lines represent contours of the distribution ranges of 22 HGDP-CEPH Yoruba individuals, 17 HGDP-CEPH East Highlands individuals, and three HGDP-CEPH Middle Eastern populations, respectively. Locations of non-HGDP-CEPH individuals are indicated by the same symbols as in Figure 2.

Using the MS5435 subset, we sought to verify the accuracy of the individual labels and the genotypic alignment of the source data sets (Table 1). MDS plots of allele-sharing-distances illustrate that in the first two dimensions, indigenous individuals from the same geographic region largely cluster together, and admixed individuals lie between the clusters of their ancestral populations (Figure 3A). These results support the inference that the individual labels in the aligned human data are correct.

To further demonstrate that after merging, major systematic genotype differences did not exist between data sets of origin, we compared locations in the MDS plot of individuals from each data set to those of individuals in the worldwide HGDP-CEPH data set. As expected under the hypothesis of correct alignment, individuals from the Native American data set lie in or near the cluster of HGDP-CEPH Native American individuals in the worldwide MDS plot (Figure 3B). A similar pattern is observed in an MDS plot of only Native American individuals (Figure 3C), except that the Aché population forms a distinct cluster, in agreement with previous population genetic evidence of the distinctiveness of this population (Battilana *et al.* 2002; Kohlrausch *et al.* 2005; Wang *et al.* 2007; Callegari-Jacques *et al.* 2008).

Individuals in the Latino data set (Wang *et al.* 2008) lie between the HGDP-CEPH Native American and European clusters, as expected given their admixture largely from Native American and European sources (Figure 3B). If we consider only the Latino and HGDP-CEPH Native American and European populations, the Latinos lie between the Europeans and a cluster containing many of the Native Americans (Figure 3D).

Individuals from the Jewish data set predominantly lie at the intersection of the HGDP-CEPH Middle Eastern and European clusters (Figure 3B). A similar pattern is observed in an MDS plot restricted to the Jewish data set together with the HGDP-CEPH European and Middle Eastern populations (Figure 3E). The individuals from the Jewish data set lie near the intersection of the HGDP-CEPH European, Bedouin, Druze, and Palestinian clusters; this pattern accords with the analysis of Kopelman *et al.* (2009) as well as with analyses of other Jewish data sets (Need *et al.* 2009; Atzmon *et al.* 2010; Behar *et al.* 2010; Campbell *et al.* 2012).

Individuals from the Asian Indian and CGP data sets lie in or near the HGDP-CEPH Central/South Asian cluster in the worldwide MDS plot (Figure 3B). In an MDS plot of only Central/South Asian individuals (Figure 3F), we similarly observe non-Gujarati individuals from the Asian Indian and CGP data sets to lie in or near the HGDP-CEPH Central/South Asian cluster, excluding the Kalash individuals. However, Gujaratis instead form a distinct cluster, consistent with a neighbor-joining analysis of the combined Asian Indian and CGP data sets that found 100% bootstrap support for a Gujarati grouping (Pemberton *et al.* 2012).

The Pacific Islander and African data sets include populations that overlap those in the HGDP-CEPH data set, enabling more precise assessments of data-set alignment. The Pacific Islander and HGDP-CEPH data sets both contain individuals sampled from the East Highlands of New Guinea (HGDP-CEPH Papuan population, Pacific Islander East Highlands population). Comparing the location in the worldwide MDS plot of East Highlands individuals from the Pacific Islander data set to those from the HGDP-CEPH data set, the Pacific Islander individuals all lie within the cluster of HGDP-CEPH individuals (Figure 3B); a similar pattern is observed in an MDS plot of only East Highlands individuals (Figure 3G). Similarly, the Yoruba individuals from the African data set all lie within the cluster of HGDP-CEPH Yoruba individuals, both in the worldwide MDS plot (Figure 3B) and in an MDS plot of only Yoruba individuals (Figure 3H).

Because no systematic clustering of populations by data set of origin is observed for any of the source data sets, our MDS analyses support the correct alignment of genotypes in the individual data sets during construction of the combined human data set.

**Table 2:** Sample sizes of groups represented in the combined data sets

	Sample Size		
Group	MS5795	MS5547	MS5435
Africa	2448	2435	2418
Middle East	296	290	281
Europe	179	177	177
Central/South Asia	839	817	810
East Asia	300	292	291
Oceania	843	745	697
America	513	416	389
Afro-European	131	131	131
Latino	246	244	241
	**MS5879**	**MS5631**	**MS5519**
Chimpanzee	84	84	84

### Population genetic analyses

Our validated combined microsatellite data set provides opportunities for revisiting population-genetic analyses previously performed on smaller data sets, as well as for developing new analyses for which earlier data did not provide sufficient population coverage. We illustrate the utility of the data set by reporting novel observations in MDS, neighbor-joining, and expected heterozygosity analyses.

#### Multidimensional scaling:

In general, the patterns we observe in our worldwide MDS analysis of MS5435 (Figure 3A) are consistent with those observed in similar analyses of single-nucleotide polymorphism (SNP) genotypes on the HGDP-CEPH data set (Jakobsson *et al.* 2008; Li *et al.* 2008; Biswas *et al.* 2009; Wang *et al.* 2010, 2012). African, East Asian, Oceanian, and Native American populations form largely distinct clusters, while the Middle Eastern, European, and Central/South Asian populations form a central heterogeneous cluster. However, we also observe some new patterns. For example, the Native American and Oceanian clusters lie farther from the clusters corresponding to other geographic regions than has been observed in similar analyses. These differences reflect the greater sample sizes for Native American and Oceanian populations in our microsatellite data set compared to the HGDP-CEPH SNP data sets analyzed previously. If we restrict the sample size of each geographic region to 158 individuals—the smallest sample size across geographic regions—in our MDS analysis, we observe a similar pattern to that seen with the complete data set (Figure S8, A and B). However, if we instead consider a subset in which the sample sizes for individual geographic regions match those of subset H952 of the HGDP-CEPH data set (Rosenberg 2006), we instead observe the same pattern (Figure S8C) reported previously with the HGDP-CEPH data set (Jakobsson *et al.* 2008; Li *et al.* 2008; Biswas *et al.* 2009; Wang *et al.* 2010, 2012).

**Figure 4:**
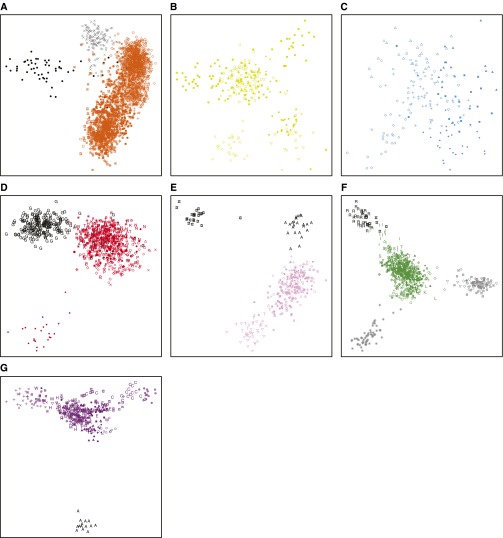
Procrustes-transformed multidimensional scaling representations of separate individual allele-sharing distance matrices from each geographic region. (A) 2418 African, (B) 281 Middle Eastern, (C) 177 European, (D) 810 Central/South Asian, (E) 291 East Asian, (F) 697 Oceanian, and (G) 389 Native American individuals in the MS5435 data set are shown. Symbols follow Figure 2, with the following exceptions for populations specifically highlighted in the text: (A) Hadza individuals are shown in black and Pygmy individuals (Baka, Bakola, Bedzan, Biaka, and Mbuti) are shown in gray; (D) Gujarati individuals are shown in black; (E) Taiwanese individuals (Ami and Taruko) are shown in black; (F) individuals from inland populations—Baining (Malasait, Marabu, and Rangulit), Ata (Lugei & Uasilau), Mamusi (Kisiluvi & Lingite), and Nakani (Loso)—are shown in gray and Polynesians (Maoris and Samoans) are shown in black; (G) Aché individuals are shown in black.

Separate MDS analyses of populations from each geographic region (Figure 4) identify population patterns not evident in the worldwide MDS plot (Figure 3A). In Africa, the click-speaking Hadza and Pygmy hunter–gatherers (Baka, Bakola, Bedzan, Biaka, and Mbuti) form distinct clusters separate from the other African populations (Figure 4A). In Oceania, the three Baining populations (Malasait, Marabu, and Rangulit) form a distinct cluster, as do the Ata (Lugei & Uasilau), Mamusi (Kisiluvi & Lingite), and Nakani (Loso) populations, and the Maoris and Samoans (Figure 4F). These patterns agree with the *Structure* analyses of Friedlaender *et al.* (2008), separating coastal Melanesian populations from both inland populations—Baining (Malasait, Marabu, and Rangulit), Ata (Lugei & Uasilau), Mamusi (Kisiluvi & Lingite), and Nakani (Loso)—and Polynesians (Maoris and Samoans).

A number of studies have investigated the correlation between geographic and genetic coordinates on the basis of multivariate statistical techniques such as MDS applied primarily to SNP genotype data, finding a strong correlation (Ramachandran *et al.* 2005; Novembre *et al.* 2008; Wang *et al.* 2010, 2012). Comparing the genetic and geographic coordinates of individuals in our worldwide MDS plot (Figure 3A), we find a lower correlation (*t_0_* = 0.342, *P* < 10^−4^) than was observed previously by Wang *et al.* (2012) with SNP genotypes in an overlapping set of individuals (*t_0_* = 0.705). This difference might partly reflect the effect of the increased presence of Native Americans and Oceanians in changing the shape of the MDS plot; however, if we restrict our MDS analysis to individuals from the same 53 populations analyzed by Wang *et al.*, our correlation still remains lower (*t_0_* = 0.299, *P* < 10^−4^). Separate comparisons in our MDS plots for each geographic region (Figure 4 and Table 3) also provide lower correlations between genes and geography than were observed by Wang *et al.* (2012). These differences might potentially reflect differences in population sets or differences in resolution between the microsatellites used here and the larger number of SNPs used by Wang *et al.*

**Table 3:** Procrustes similarity between genetic and geographic coordinates in data set MS5435

Subset	Sample Size	*t_0_*	*P*
Worldwide	4977	0.342	**<10^−4^**
Africa	2418	0.303	**2.0 × 10^−4^**
Middle East	223	0.305	0.463
Europe	158	0.237	0.150
Central/South Asia	810	0.086	0.540
East Asia	291	0.181	0.396
Oceania	688	0.352	**0.032**
America	389	0.167	0.257
HGDP-CEPH*^a^*	961	0.299	**0.014**

Tests with *P* < 0.05 are highlighted in boldface type.

a The East Highlands and Nasioi populations were used for the HGDP-CEPH Papuan and Melanesian populations, respectively.

#### Neighbor-joining:

**Figure 5:**
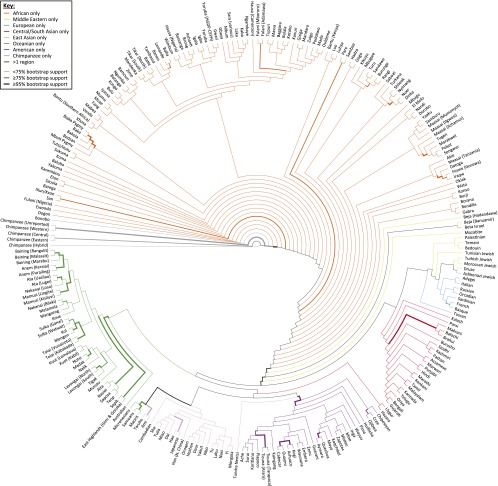
Consensus neighbor-joining tree of the 249 non-admixed human populations and six chimpanzee populations. In 1000 bootstrap replicates using 246 microsatellite markers, the thickest edges have at least 95% bootstrap support, and the edges of intermediate thickness have at least 75% support. Rooting the tree at the human–chimpanzee divergence, if all populations subtended by an edge are from the same geographic region, the edge is drawn in the color representing that region; otherwise, it appears in black.

Neighbor-joining analysis of population structure in the MS5519 human–chimpanzee data set provides 100% bootstrap support for a separate grouping of the chimpanzee populations (Figure 5). It also provides 100% support for the separate grouping of bonobos within the chimpanzee clade, and 88.5% support for the separate grouping of the western and unreported common chimpanzees, in agreement with the inference of Becquet *et al.* (2007) that the unreported individuals are predominantly western. Within the human clade, the separate grouping of non-African populations has 90.0% support. Groupings of all Native American populations and all Oceanian populations excluding the Australians, Micronesians, Maoris, and Samoans have 99.9% and 100% support, respectively. Interestingly, the grouping of the Micronesian, Maori, and Samoan populations with the Taiwanese aboriginal Ami and Taruko populations has 90.5% support. This observation is compatible with the support provided by Friedlaender *et al.* (2008) to the “express train” model for the colonization of Polynesia, which posits that populations of Micronesia and Polynesia derive their ancestry largely from a migration outward from Taiwan (Diamond 1988; Hurles *et al.* 2003).

#### Heterozygosity:

**Figure 6:**
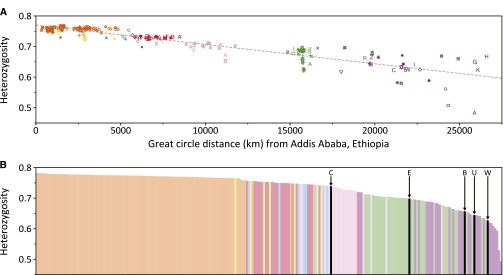
Mean expected heterozygosity across loci. (A) Decrease in heterozygosity in 239 non-admixed non-Jewish populations in the MS5795 human data set, as a function of distance from Addis Ababa, Ethiopia (9°N, 38°E). The coefficient of determination is *R^2^* = 0.841. Symbols follow Figure 2. (B) Heterozygosity in 244 non-admixed non-Jewish populations in the MS5879 human–chimpanzee data set. Populations are ordered by decreasing expected heterozygosity and are colored by geographic affiliation as in Figure 2; chimpanzee bars appear in black. Key: B, bonobo; C, central common chimpanzees; E, eastern common chimpanzees; U, unreported common chimpanzees; W, western common chimpanzees. In both plots, populations with fewer than five individuals are excluded (Barega, Dogon, Eton, Ewondo, Fulani [Nigeria], and hybrid chimpanzees). Expected heterozygosities are provided for human populations in Table S20 and for chimpanzee populations in Table S23.

Previous studies have identified a linear correlation between genetic diversity, as measured by expected heterozygosity, and geographic distance from points in Africa (Prugnolle *et al.* 2005a; Ramachandran *et al.* 2005). Using the 645 loci in MS5795, we found a similar decay of expected heterozygosity with increasing geographic distance from East Africa (Figure 6A, *R^2^* = 0.841); an analogous decay is observed with the 246 microsatellites in the combined human–chimpanzee MS5879 (*R^2^* = 0.820). Among the chimpanzees, expected heterozygosity is highest in the central group and lowest in the western group (Table S23), and its range encompasses values observed in human populations from East Asia to the Americas (Figure 6B). We note, however, that the microsatellites in our data sets were ascertained for length and variability in human samples (Ghebranious *et al.* 2003) and then applied to chimpanzees (Becquet *et al.* 2007). Thus, while genome comparisons hint at genuine differences in variability for orthologous microsatellites in humans and chimpanzees (Cooper *et al.* 1998; Webster *et al.* 2002; Vowles and Amos 2006; Kelkar *et al.* 2008), the variability in chimpanzees of the loci we examined might be systematically lower than would be obtained for loci ascertained to be variable in both species (Kelkar *et al.* 2008).

### Conclusions

We have combined eight human microsatellite data sets at the loci that appear in all the data sets. As previous compilations (Rosenberg *et al.* 2006; Wang *et al.* 2007, 2008; Friedlaender *et al.* 2008; Kopelman *et al.* 2009; Tishkoff *et al.* 2009; Hunley *et al.* 2012; Pemberton *et al.* 2012) have combined at most half of the data sets we included here, we have assembled the largest microsatellite data set of human populations reported to date. We have augmented the data with similar data for chimpanzees, and we report both the combined human and human–chimpanzee data sets (File S1). These resources offer new opportunities for more complete analyses of patterns of human genetic variation in numerous areas of application.

## Supplementary Material

Supporting Information
