# Supplementary material for: Population Structure in a Comprehensive Genomic Data Set on Human Microsatellite Variation
Source: G3 (Bethesda). 2013 May 1;3(5):891–907. doi: 10.1534/g3.113.005728 (PMC3656735; doi:10.1534/g3.113.005728)
Supplement: Supporting Information [file supp_3_5_891__index.html]

Supporting Information 

# Population Structure in a Comprehensive Genomic Data Set on Human Microsatellite Variation

## Supporting Information for Pemberton, DeGiorgio, and Rosenberg, 2013

**Files in this Data Supplement:**

- Supporting Information - Figures S1-S8, File S1, Tables S1-S26, and Supporting References (PDF, 5 MB)
- Figure S1 - Intra-population allele-sharing for pairs of individuals in those populations in the African data set for which we inferred at least one relative pair (PDF, 581 KB)
- Figure S2 - Intra-population allele-sharing for pairs of individuals in the Latino data set (PDF, 442 KB)
- Figure S3 - Intra-population allele-sharing for pairs of individuals in the Pacific Islander data set (part 1) (PDF, 661 KB)
- Figure S4 - Intra-population allele-sharing for pairs of individuals in the Pacific Islander data set (part 2). (PDF, 607 KB)
- Figure S5 - Intra-population allele-sharing for pairs of individuals in the Pacific Islander data set (part 3). (PDF, 446 KB)
- Figure S6 - Inter-population allele-sharing for pairs of individuals in each of eight subsets that group populations by their geographic affiliation (Africa, the Middle East, Europe, Central/South Asia, East Asia, Oceania, and the Americas) or admixture status (Afro-European). (PDF, 1 MB)
- Figure S7 - Intra- and inter-population allele-sharing for pairs of individuals in the chimpanzee data set (PDF, 483 KB)
- Figure S8 - The effect of sample size on population patterns in worldwide multidimensional scaling (MDS) analyses (PDF, 1 MB)
- Table S1 - Allele size adjustments used to make the Pacific Islander data set comparable to the combined HGDP-CEPH, Native American, Latino, Jewish, Asian Indian, and CGP data set (PDF, 48 KB)
- Table S2 - Allele size adjustments used to make the African data set comparable to the combined HGDP-CEPH, Native American, Latino, Jewish, Asian Indian, CGP, and Pacific Islander data set (PDF, 46 KB)
- Table S3 - 11 loci excluded from the combined data set of 656 loci due to >10% missing data (PDF, 38 KB)
- Table S4 - 68 individuals excluded from the combined data set of 5916 individuals due to >27.5% missing data (PDF, 46 KB)
- Table S5 - Two previously unreported intra-population first-degree relative pairs in the African data set (PDF, 38 KB)
- Table S6 - 12 previously unreported intra-population second-degree relative pairs in the African data set (PDF, 41 KB)
- Table S7 - Two previously unreported intra-population first-degree relative pairs in the Latino data set (PDF, 38 KB)
- Table S8 - Four previously unreported intra-population second-degree relative pairs in the Latino data set (PDF, 38 KB)
- Table S9 - Six previously unreported intra-population monozygotic pairs in the Pacific Islander data set (PDF, 38 KB)
- Table S10 - 127 previously unreported intra-population first-degree relative pairs in the Pacific Islander data set (PDF, 52 KB)
- Table S11 - 87 previously unreported intra-population second-degree relative pairs in the Pacific Islander data set (PDF, 48 KB)
- Table S12 - 13 previously unreported intra-population parent/parent/offspring trios in the Pacific Islander data set (PDF, 41 KB)
- Table S13 - One previously unreported inter-population first-degree relative pair in the African data set (PDF, 27 KB)
- Table S14 - One previously unreported inter-population second-degree relative pair in the African data set (PDF, 27 KB)
- Table S15 - Two previously unreported inter-population monozygotic pairs in the Pacific Islander data set (PDF, 27 KB)
- Table S16 - 24 previously unreported inter-population first-degree relative pairs in the Pacific Islander data set (PDF, 35 KB)
- Table S17 - 54 previously unreported inter-population second-degree relative pairs in the Pacific Islander data set (PDF, 39 KB)
- Table S18 - Three previously unreported inter-population parent/parent/offspring trios in the Pacific Islander data set (PDF, 29 KB)
- Table S19 - 10 previously unreported inter-population second-degree relative pairs in the Native American data set (PDF, 31 KB)
- Table S20 - 267 human populations present in the combined data set together with their geographic coordinates, sample sizes, and mean heterozygosities (PDF, 90 KB)
- Table S21 - The 248 individuals in MS5795 but not in MS5547 (PDF, 49 KB)
- Table S22 - The 112 individuals in MS5547 but not in MS5435 (PDF, 45 KB)
- Table S23 - Six chimpanzee populations present in the chimpanzee data set together with their sample sizes and average heterozygosities in the combined human-chimpanzee data set (PDF, 43 KB)
- Table S24 - Three individuals with >27.5% missing data in the combined human-chimpanzee data set (PDF, 38 KB)
- Table S25 - 27 loci from the combined human data set of 645 loci with no genotype data in at least one population (PDF, 40 KB)
- Table S26 - 10 loci from the combined human-chimpanzee data set of 246 loci with no genotype data in at least one population (PDF, 38 KB)
- Supporting References - PDF, 40 KB
- File S1 - Genotype data sets (.zip, 10 MB)
