# Supplementary material for: Population Structure in a Comprehensive Genomic Data Set on Human Microsatellite Variation
Source: G3 (Bethesda). 2013 May 1;3(5):891–907. doi: 10.1534/g3.113.005728 (PMC3656735; doi:10.1534/g3.113.005728)
Supplement: Supporting Information [file supp_g3.113.005728_005728SI.pdf]

G3 · Genes | Genomes | Genetics

Population structure in a comprehensive genomic data set on human microsatellite variation

Trevor J. Pemberton[§,1], Michael DeGiorgio[†], and Noah A. Rosenberg[§]

[§]Department of Biology, Stanford University, Stanford, CA 94305

[†]Department of Integrative Biology, University of California, Berkeley, CA 94720

[1]Present address: Department of Biochemistry and Medical Genetics, University of Manitoba, Winnipeg, MB R3E 0J9

Author to whom correspondence should be addressed:
Dr. Trevor J. Pemberton
Department of Biochemistry and Medical Genetics,
745 Bannatyne Avenue, Winnipeg, MB R3E 0J9
Tel: (204) 789-3602
E-mail: pembertont@med.umanitoba.ca

DOI: 10.1534/g3.113.005728

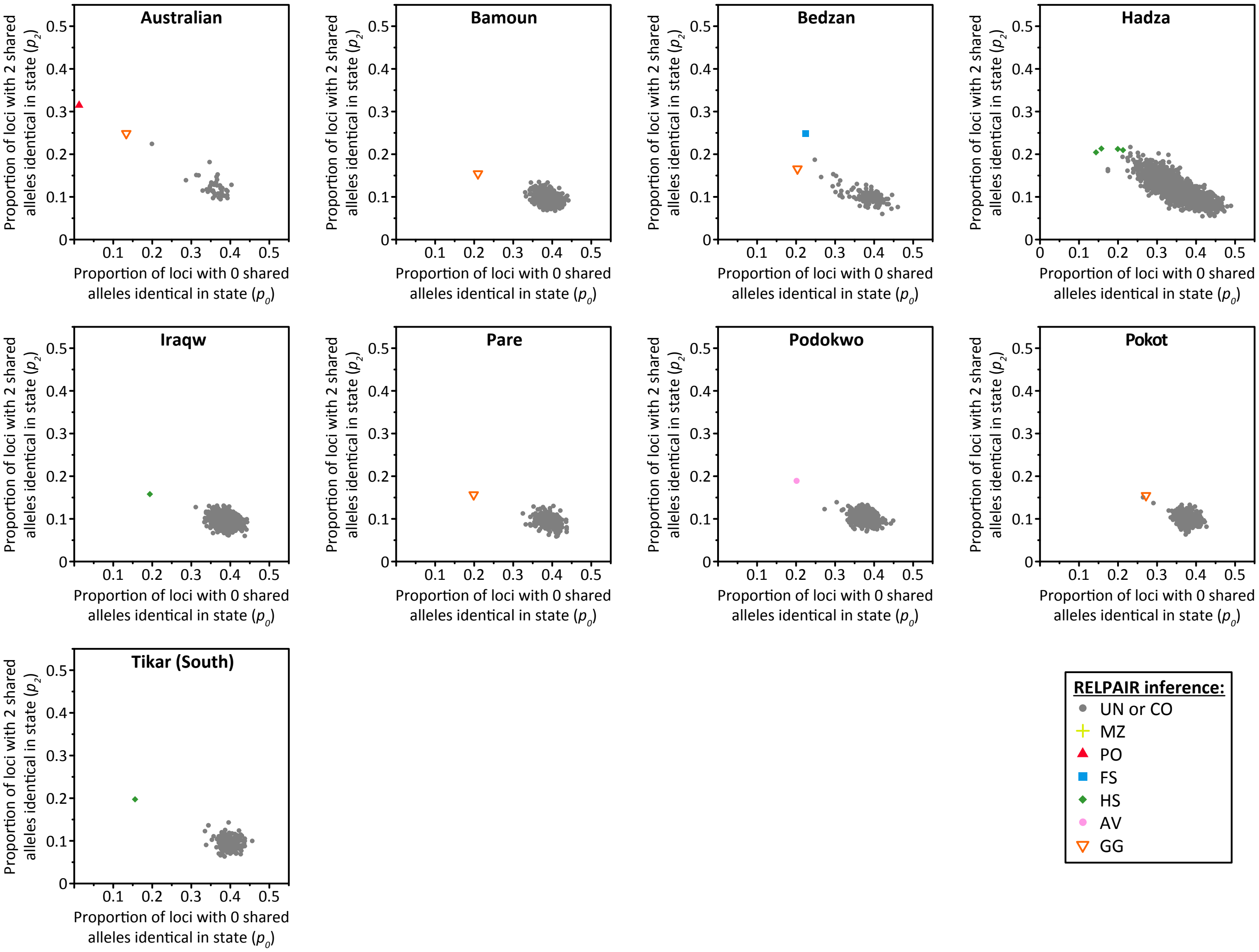

Figure S1 Intra-population allele-sharing for pairs of individuals in those populations in the African data set for which we inferred at least one relative pair. First- and second-degree intra-population relative pairs are reported in Tables S5 and S6, respectively.

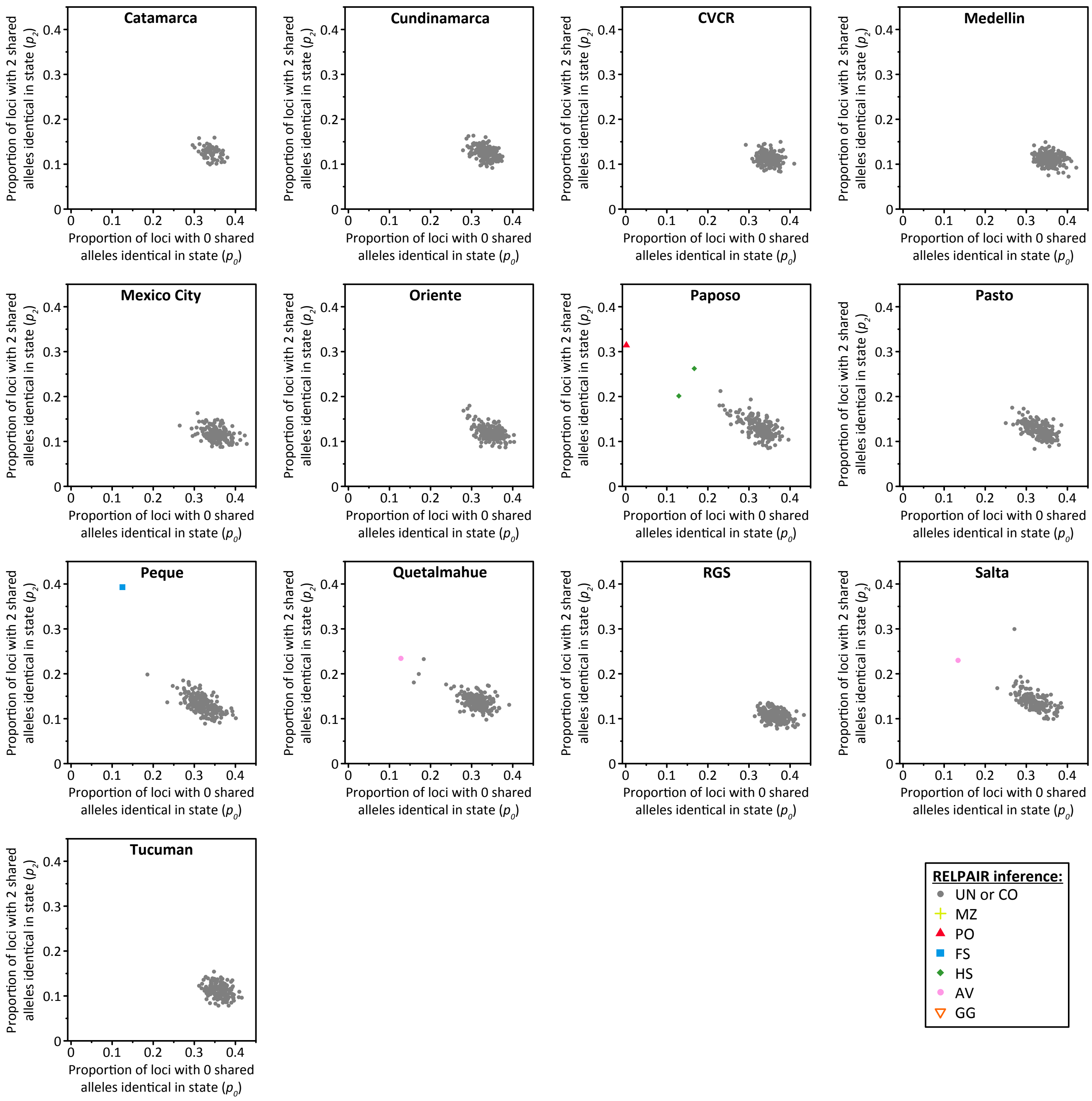

Figure S2 Intra-population allele-sharing for pairs of individuals in the Latino data set. First- and second-degree relative pairs are reported in Tables S7 and S8, respectively.

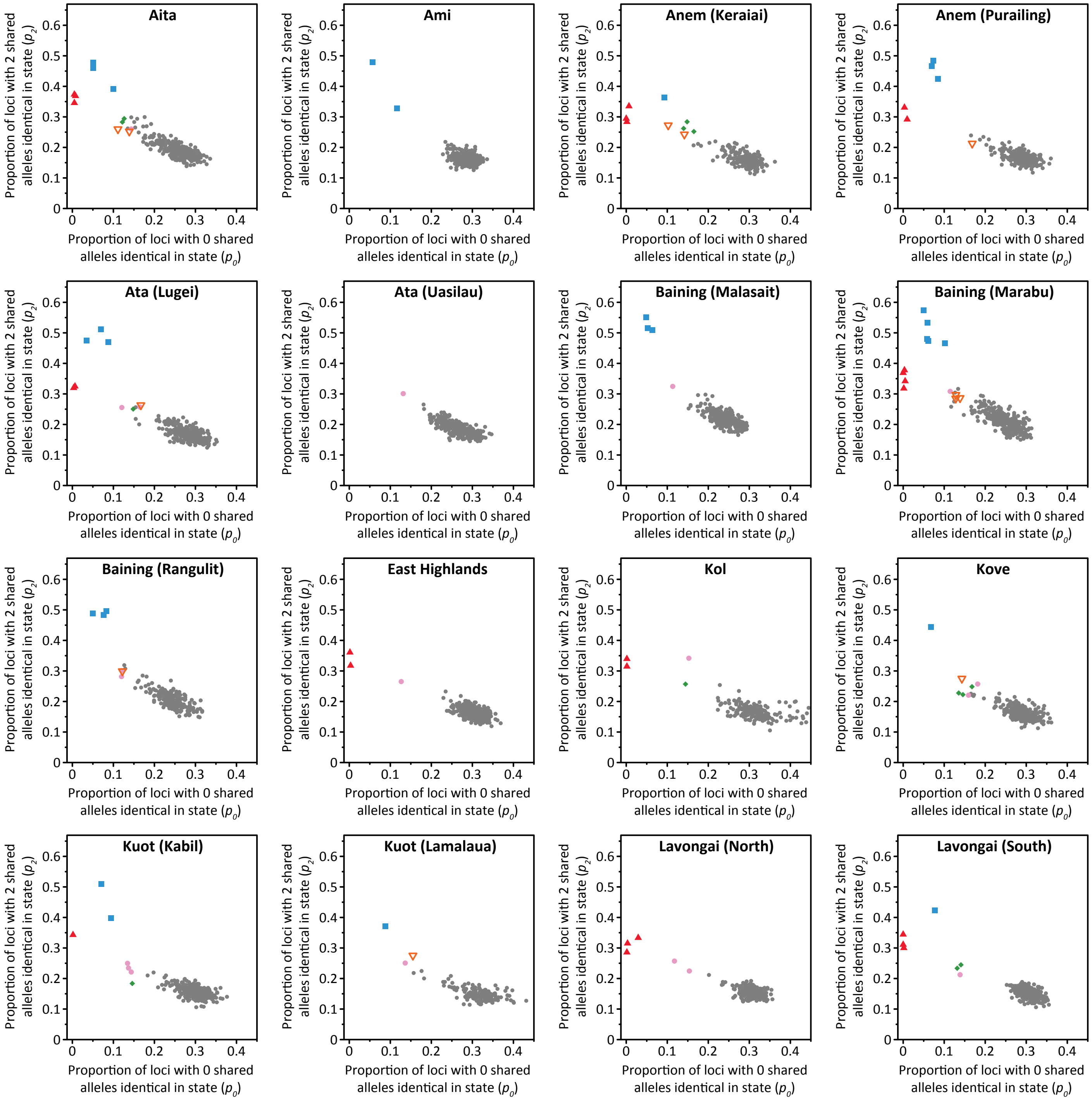

Figure S3 Intra-population allele-sharing for pairs of individuals in the Pacific Islander data set (part 1). Symbols are as defined in Figure S1. First- and second-degree relative pairs are reported in Tables S10 and S11, respectively.

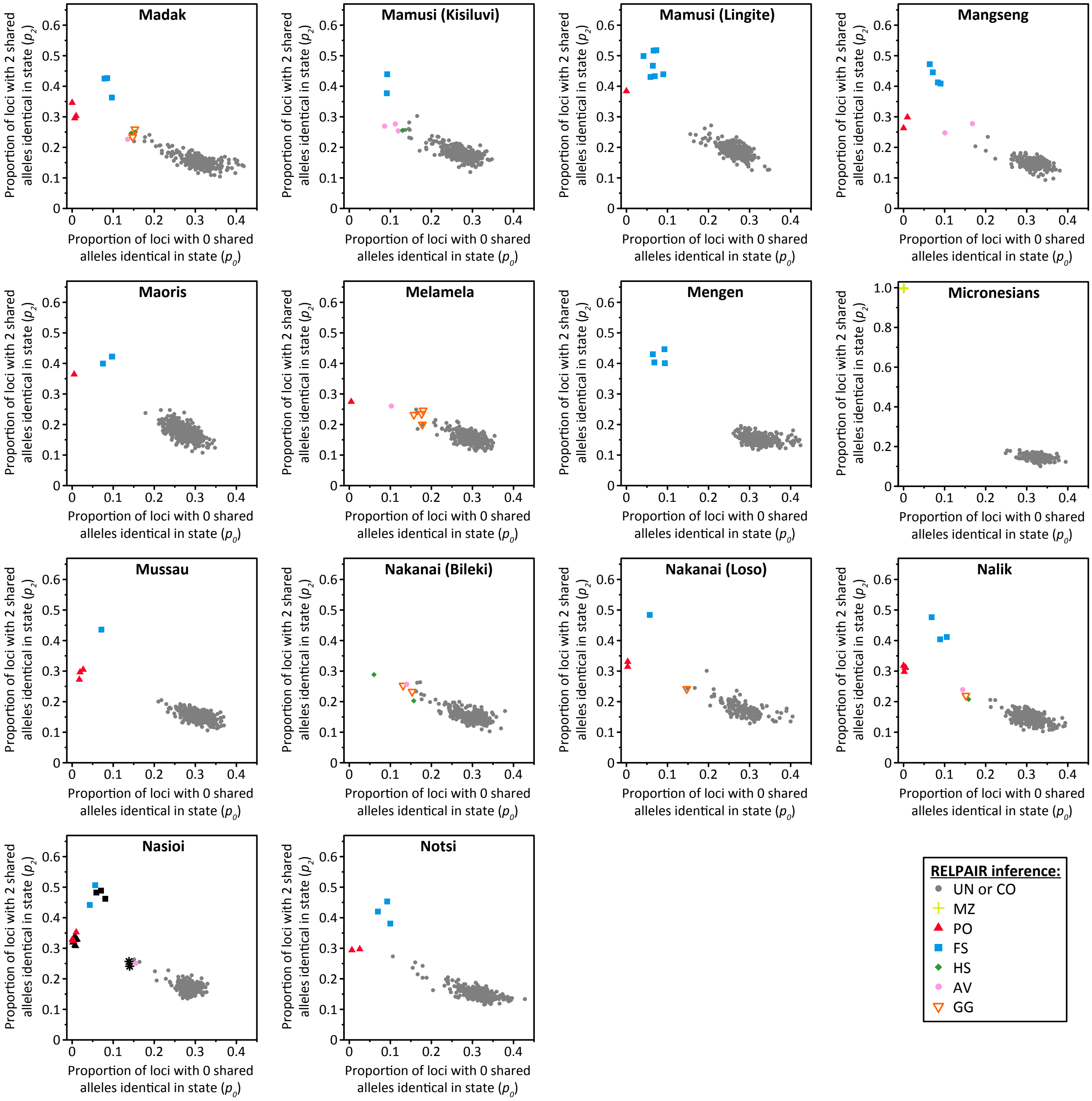

Figure S4 Intra-population allele-sharing for pairs of individuals in the Pacific Islander data set (part 2). Symbols colored black in the Nasioi population are relative pairs identified in Rosenberg [1]. Monozygotic, first-degree, and second-degree relative pairs are reported in Tables S9, S10, and S11, respectively.

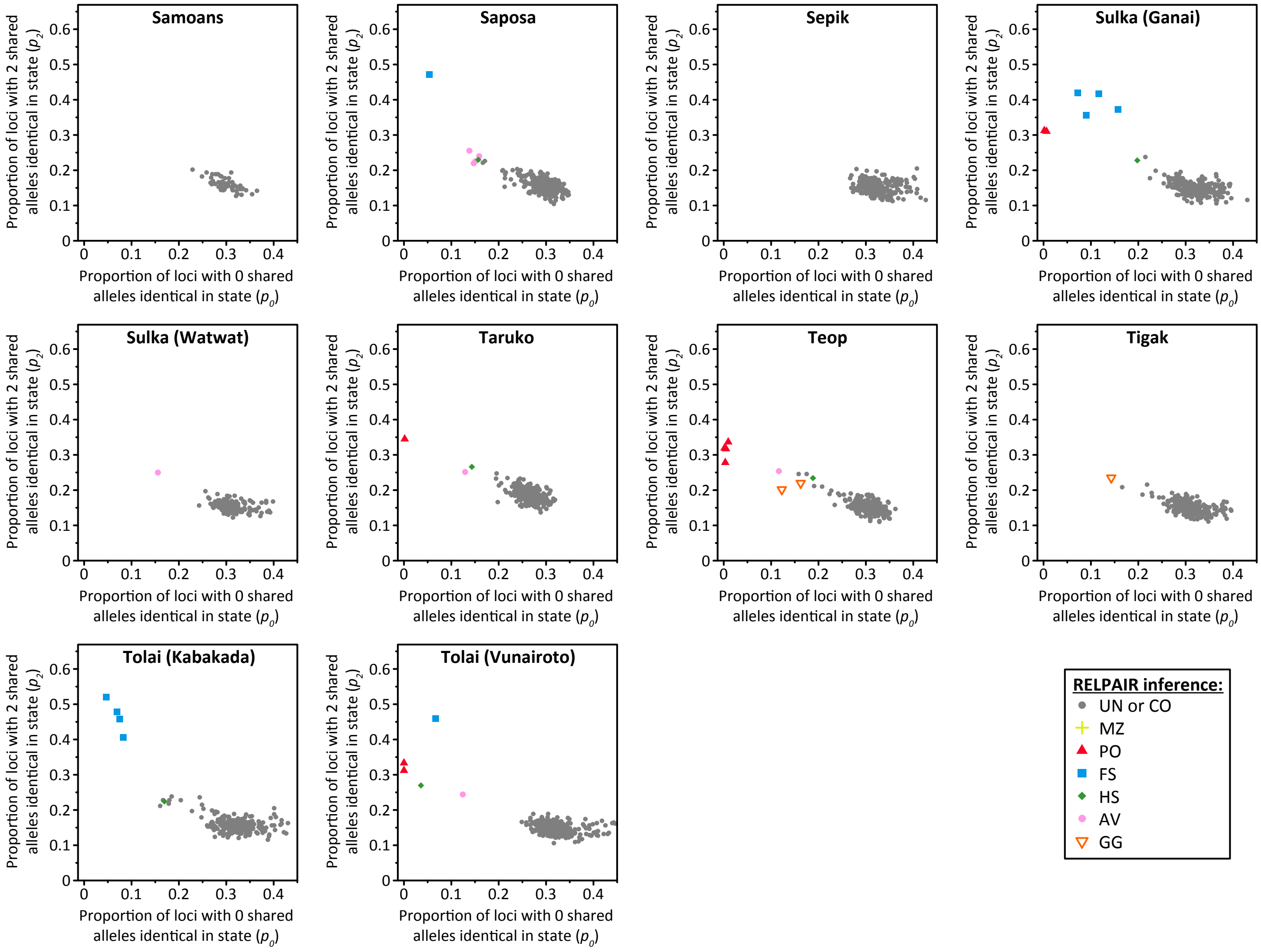

Figure S5 Intra-population allele-sharing for pairs of individuals in the Pacific Islander data set (part 3). First- and second-degree relative pairs are reported in Tables S10 and S11, respectively.

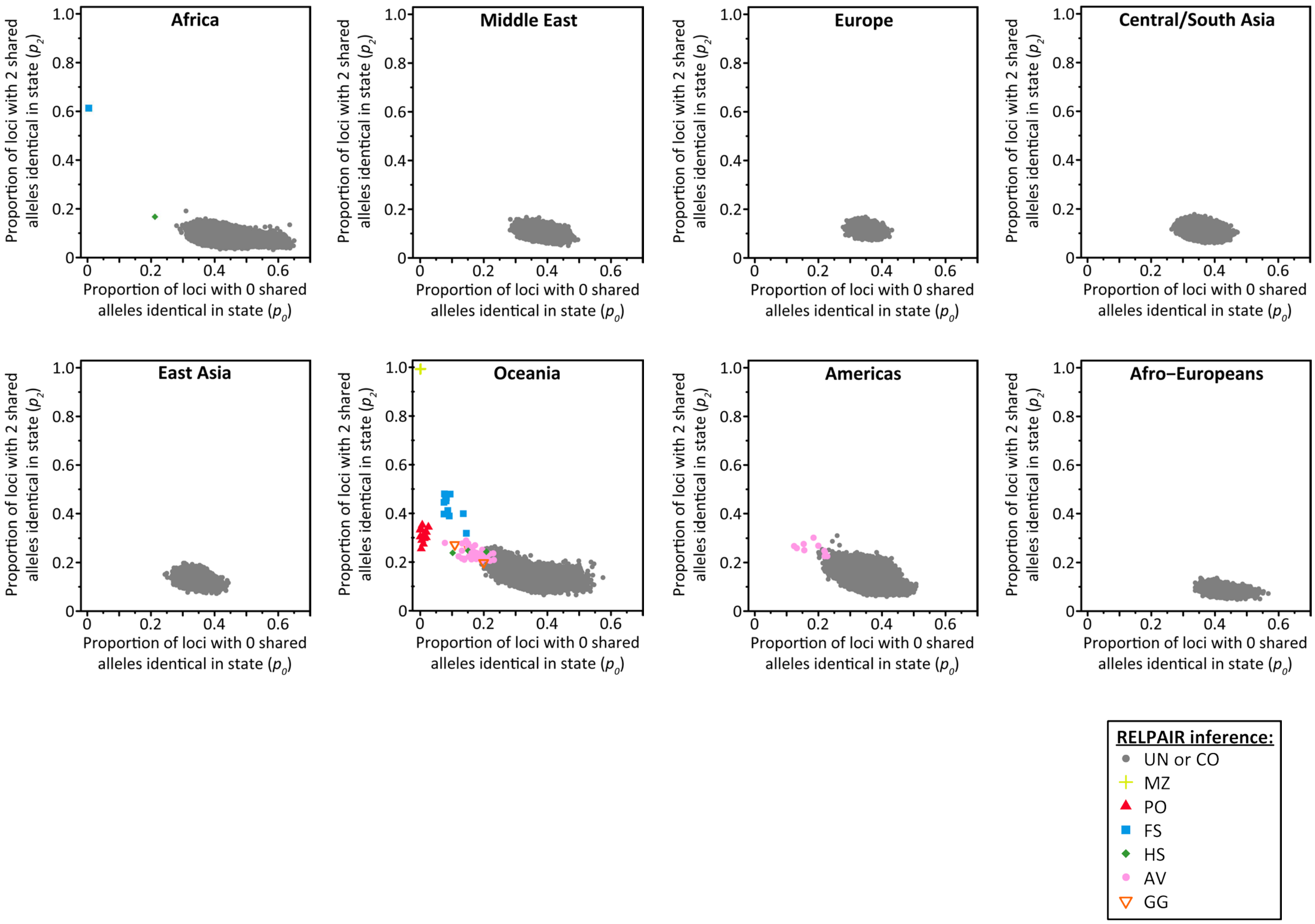

Figure S6 Inter-population allele-sharing for pairs of individuals in each of eight subsets that group populations by their geographic affiliation (Africa, the Middle East, Europe, Central/South Asia, East Asia, Oceania, and the Americas) or admixture status (Afro-European). Latino individuals were included in the Americas analysis, as they were genotyped concurrently with the Native American data set. First- and second-degree relative pairs in the Africa analysis are reported in Tables S13 and S14, respectively. Monozygotic, first-degree, and second-degree relative pairs in the Oceania analysis are reported in Tables S15, S16, and S17, respectively. Second-degree relative pairs in the Americas analysis are reported in Table S19.

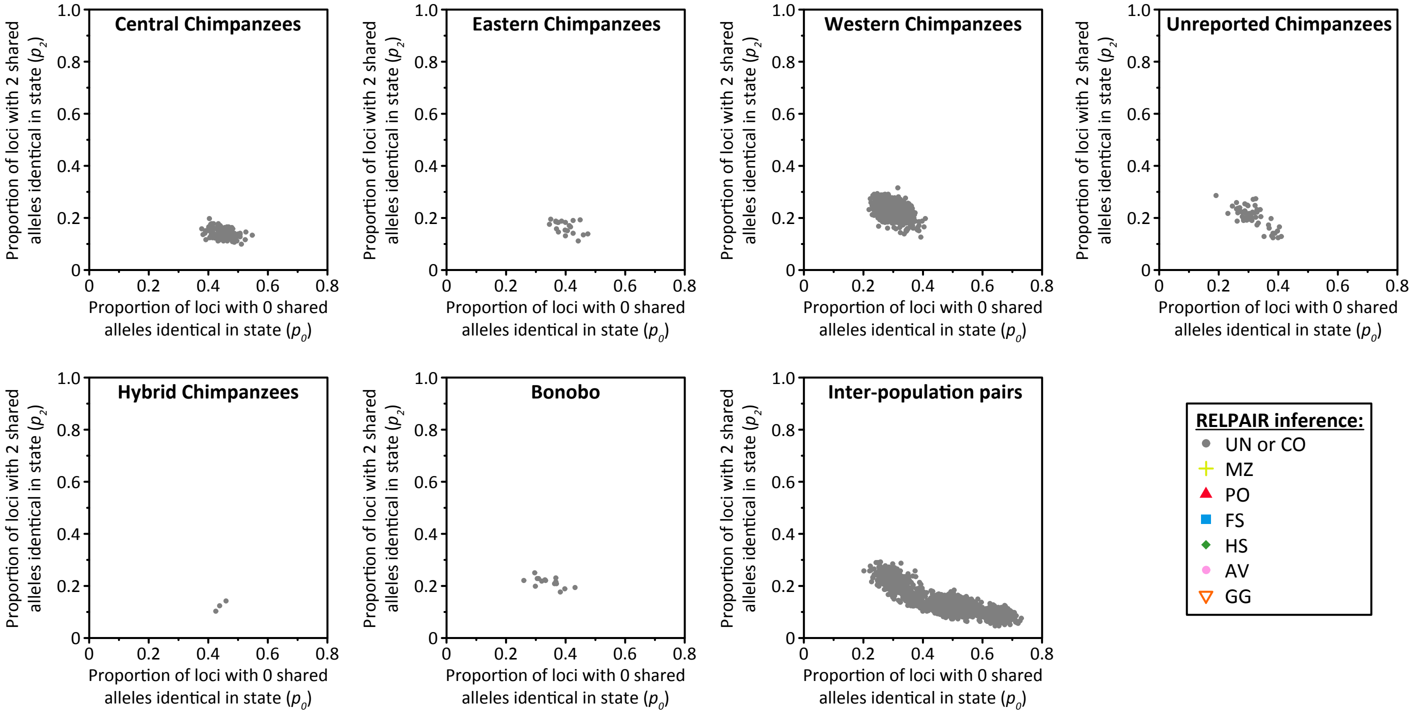

Figure S7 Intra- and inter-population allele-sharing for pairs of individuals in the chimpanzee data set.

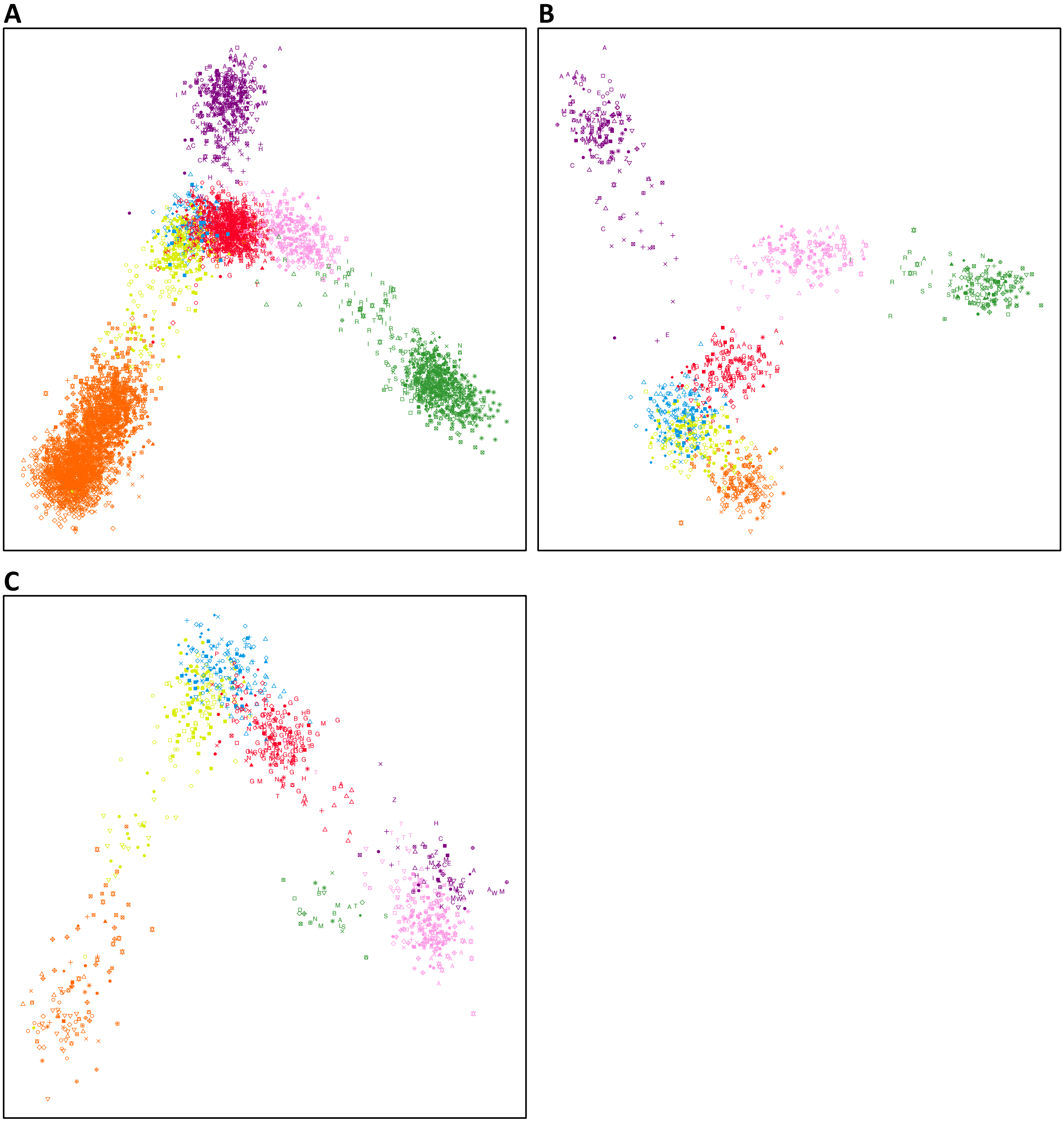

Figure S8 The effect of sample size on population patterns in worldwide multidimensional scaling (MDS) analyses. Procrustes-transformed MDS representations of pairwise allele-sharing distances between (A) 5063 non-admixed individuals in the MS5435 data set, (B) 1106 non-admixed individuals from the MS5435 data set where each geographic region is represented by a random sample of 158 individuals—the smallest sample size across geographic regions—with membership to that region, and (C) 952 non-admixed individuals from the MS5435 data set where each geographic region is represented by the same number of individuals as in subset H952 of the HGDP-CEPH data set [1], chosen at random from all individuals with membership to that region. Individuals are colored by geographic affiliation and indicated by the symbols in Figure 2.

File S1

Genotype data sets

File S1 is a Zip archive containing the genotype data of the combined human data set of 5795 individuals and the combined human-chimpanzee data set of 5879 individuals in the format used by the Structure program, along with a list of individual memberships in the subsets described in the paper

File S1 is available for download at http://www.g3journal.org/lookup/suppl/doi:10.1534/g3.113.005728/-/DC1

Table S1 Allele size adjustments used to make the Pacific Islander data set comparable to the combined HGDP-CEPH, Native American, Latino, Jewish, Asian Indian, and CGP data set

ID in combined data set	ID in Pacific Islander data set	Amount added to genotypes in the Pacific Islander data set (*c**)
AAT107_16	AAT107Z	-53
AAT200_1	AAT200ZP	-137
AATA019_8	AATA019ZP	-131
ATA50C05_6	ATA50C05ZP	116
ATA70B03P_14	ATA70B03ZP	-124
ATA77F05_14	ATA77F05Z	74
D10S1221	ATA21A03Z	73
D10S1412	ATA31G11P	-7
D10S1430	GATA84C01ZP	-39
D11S1304	UT2095M	-1
D11S1392	GATA6B09P	-7
D11S1984	GGAA17G05P	-7
D11S1998	GATA23E06L	-3
D11S1999	GATA23F06L	-3
D11S2000	GATA28D01M	-1
D12S1042	ATA27A06P	-7
D12S1045	ATA29A06P	-7
D12S1052	GATA26D02M	-1
D12S1064	GATA63D12P	-7
D12S1300	GATA85A04M	-1
D12S1301	GATA91H06M	-1
D12S2070	ATA25F09M	-1
D13S1807	GATA11C08P	-7
D13S787	GATA23C03P	-7
D13S796[a]	GATA51B02ZP	-46
D13S895	GGAA22G01ZP	17
D14S608	GATA43H01M	-1
D14S617	GGAA21G11L	-2
D15S1515	GATA197B10P	-7
D15S816	GATA73F01M	-1
D16S2624	GATA81D12M	-1
D16S3253	GATA22F09P	-7
D17S1298	GAAT2C03P	-7
D17S2180	ATC6A06M	-1
D17S2195	ATA58A02P	-7
D18S1376[b]	GATA185C06Z	-17
D19S589	GATA29B01L	1
D19S591	GATA44F10P	-7
D1S1596	GATA26G09P	-7
D1S1612	GGAA3A07M	-1
D1S1627	ATA25E07M	-1
D1S1728	GATA109Z	-130
D1S3669	GATA29A05P	-7
D20S477	GATA29F06Z	-1
D21S1411	UT1355Z	-3

D22S686	GGAA10F06M	-1
D2S1352	ATA27D04P	-7
D2S1363	GATA23D03ZP	-107
D2S1384	GATA52A04M	-1
D2S1391	GATA65C03M	-1
D2S1394	GATA69E12M	-1
D2S1400	GGAA20G10M	-1
D2S2944	GATA30E06P	-7
D2S2968	GATA178G09M	-1
D3S1744[c]	GATA3C02ZP	-26
D3S1768	GATA8B05M	-1
D3S2427	GATA22F11NZ	58
D3S2432	GATA27C08P	-7
D3S4529	GATA128C02M	-1
D4S1627	GATA7D01ZP	37
D4S1652	GATA5B02M	-1
D4S2366	GATA22G05M	-1
D4S2397	ATA27C07P	-7
D4S2417	GATA42H02P	-8
D4S2623	GATA62A12Z	-35
D4S2632	GATA72G09Z	25
D5S1456	GATA11A11P	-7
D5S1462	GATA3H06M	-1
D5S1470	GATA7C06M	-1
D5S1480	ATA23A10M	-1
D5S1725[d]	GATA89G08Z	31
D5S2488	ATA20G07M	-1
D6S1017	GGAT3H10M	-1
D6S1027	ATA22G07P	-7
D6S2436	GATA165G02M	-1
D7S1802	GATA41G07M	-1
D7S1818	GATA24D12P	-7
D7S2204	GATA73D10L	4
D7S2477	035XB9ZP	-70
D7S3056	GATA24F03ZP	7
D7S3070	GATA189C06M	-1
D8S1048	UT7129L	1
D8S1110	GATA8G10M	-1
D8S1132	GATA26E03M	-1
D8S1477[e]	GGAA20C10Z	-104
D8S373	UT721M	-1
D8S592	GATA6B02P	-7
D9S1120	GATA81C04M	-1
D9S2169	GATA62F03M	-1
D9S910	ATA18A07M	-1
GATA138B05_5	GATA138B05ZP	49
GATA169E06_14	GATA169E06ZP	38
GATA66D01_2	GATA66D01ZP	-110
GGAA30H04_14	GGAA30H04ZP	-103
NA.D18S.1	GATA178F11Z	67

NA.D1S.3	GATA133A08Q	-4
TPO.D2S	SRAP	-7

[a]Friedlaender *et al*. [2] used an adjustment of -45 nt.

[b]This locus was not present in the list of adjusted loci reported by Friedlaender *et al*. [2].

[c]Friedlaender *et al*. [2] used an adjustment of -25 nt.

[d]Friedlaender *et al*. [2] used an adjustment of 27 nt.

[e]Friedlaender *et al*. [2] used an adjustment of -103 nt.

Table S2 Allele size adjustments used to make the African data set comparable to the combined HGDP-CEPH, Native American, Latino, Jewish, Asian Indian, CGP, and Pacific Islander data set

ID in combined data set	ID in African data set	Amount added to genotypes in the African data set (c^*)
D10S1412	ATA31G11P	-7
D11S1304	UT2095M	-1
D11S1392	GATA6B09P	-7
D11S1984	GGAA17G05P	-7
D11S1998	GATA23E06L	-3
D11S1999	GATA23F06L	-3
D11S2000	GATA28D01M	-1
D12S1042	ATA27A06P	-7
D12S1045	ATA29A06P	-7
D12S1052	GATA26D02M	-1
D12S1064	GATA63D12P	-7
D12S1300	GATA85A04M	-1
D12S1301	GATA91H06M	-1
D12S2070	ATA25F09M	-1
D13S1807	GATA11C08P	-7
D13S787	GATA23C03P	-7
D13S796	GATA51B02M	-1
D14S608	GATA43H01M	-1
D14S617	GGAA21G11L	-2
D15S1515	GATA197B10P	-7
D15S816	GATA73F01M	-1
D16S2624	GATA81D12M	-1
D16S3253	GATA22F09P	-7
D17S1298	GAAT2C03P	-7
D17S2180	ATC6A06M	-1
D17S2195	ATA58A02P	-7
D18S1376[a]	GATA185C06Z	-17
D19S589	GATA29B01L	1
D19S591	GATA44F10P	-7
D1S1596	GATA26G09P	-7
D1S1612	GGAA3A07M	-1
D1S1627	ATA25E07M	-1
D1S3669	GATA29A05P	-7
D20S477	GATA29F06Z	-1
D22S686	GGAA10F06M	-1
D2S1352	ATA27D04P	-7
D2S1384	GATA52A04M	-1
D2S1391	GATA65C03M	-1
D2S1394	GATA69E12M	-1
D2S1400	GGAA20G10M	-1
D2S2944	GATA30E06P	-7
D2S2968	GATA178G09M	-1
D3S1744	GATA3C02M	-1
D3S1768	GATA8B05M	-1
D3S2427	GATA22F11NZ	58

D3S2432	GATA27C08P	-7
D3S4529	GATA128C02M	-1
D4S1627	GATA7D01ZP	37
D4S1652	GATA5B02M	-1
D4S2366	GATA22G05M	-1
D4S2397	ATA27C07P	-7
D4S2417	GATA42H02P	-8
D5S1456	GATA11A11P	-7
D5S1462	GATA3H06M	-1
D5S1470	GATA7C06M	-1
D5S1480	ATA23A10M	-1
D5S1725[b]	GATA89G08Z	31
D5S2488	ATA20G07M	-1
D6S1017	GGAT3H10M	-1
D6S1027	ATA22G07P	-7
D6S2436	GATA165G02M	-1
D7S1802	GATA41G07M	-1
D7S1818	GATA24D12P	-7
D7S2204	GATA73D10L	4
D7S3070	GATA189C06M	-1
D8S1048	UT7129L	1
D8S1110	GATA8G10M	-1
D8S1132	GATA26E03M	-1
D8S1477	GGAA20C10M	-1
D8S373	UT721M	-1
D8S592	GATA6B02P	-7
D9S1120	GATA81C04M	-1
D9S2169	GATA62F03M	-1
D9S910	ATA18A07M	-1
NA.D18S.1	GATA178F11Z	67
NA.D1S.3	GATA133A08Q	-4
TPO.D2S	SRAP	-7

[a]This locus was not present in the list of adjusted loci reported by Tishkoff *et al*. [3].

[b]Tishkoff *et al*. [3] used an adjustment of 27 nt.

Table S3 11 loci excluded from the combined data set of 656 loci due to >10% missing data

Locus ID	Fraction of individuals with missing genotypes
D2S1780	0.287
D9S938	0.192
GTTTT002	0.188
AGAT135	0.177
AAT258	0.175
NA.D1S.1	0.171
D1S2134	0.167
AAT267	0.161
AGAT017	0.157
D1S3721	0.147
D3S2432	0.147

Table S4 68 individuals excluded from the combined data set of 5916 individuals due to >27.5% missing data

Population			Identification number of individual	Fraction of loci with missing genotypes
ID	Name	Data set of origin		
1009	Mengen	Pacific Islander	14131	0.868
1009	Mengen	Pacific Islander	14141	0.786
1116	Ngumba	African	70753	0.586
1280	Temani	African	103376	0.524
1278	Dogon	African	103335	0.513
1280	Temani	African	103372	0.510
811	Chipewyan	Native American	2384	0.501
1013	Nakanai (Bileki)	Pacific Islander	15241	0.491
845	Ticuna (Arara)	Native American	2547	0.490
1022	Tolai (Kabakada)	Pacific Islander	22071	0.478
824	Zapotec	Native American	2091	0.471
1017	Ata (Uasilau)	Pacific Islander	7003	0.471
1272	Cape Mixed Ancestry	African	103215	0.468
1005	Anem (Keraiai)	Pacific Islander	4031	0.457
1268	Nuer	African	73132	0.457
884	Catamarca	Latino	2154	0.456
1273	Venda	African	103242	0.456
1254	Wimbum	African	72932	0.451
871	Oriente	Latino	2219	0.440
822	Mixtec	Native American	2042	0.434
1034	Teop	Pacific Islander	35111	0.434
843	Inga	Native American	2509	0.431
831	Guaymi	Native American	2009	0.420
690	Tundra Nentsi	Native American	2457	0.419
1271	Sara (Various)	African	73026	0.419
1278	Dogon	African	103333	0.419
1241	Maasai (Ilchamus)	African	72708	0.411
1272	Cape Mixed Ancestry	African	103216	0.411
837	Kaingang	Native American	2757	0.408
824	Zapotec	Native American	2082	0.406
831	Guaymi	Native American	2003	0.398
884	Catamarca	Latino	2150	0.398
836	Ache	Native American	2742	0.389
1235	Gabra	African	72527	0.388
1278	Dogon	African	103339	0.386
1278	Dogon	African	103352	0.383
1276	Koma	African	103304	0.369
1278	Dogon	African	103332	0.367
1012	Kol	Pacific Islander	8281	0.364
1274	!Xun/Kxoe	African	103258	0.364
849	Arhuaco	Native American	2733	0.363
1280	Temani	African	103385	0.353
1272	Cape Mixed Ancestry	African	103186	0.349
836	Ache	Native American	2737	0.347
1273	Venda	African	103248	0.346
1101	Hadza	African	71463	0.343
54	Hazara	HGDP-CEPH	127	0.341

1022	Tolai (Kabakada)	Pacific Islander	22041	0.341
812	Cree	Native American	2403	0.340
1272	Cape Mixed Ancestry	African	103211	0.340
1274	!Xun/Kxoe	African	103260	0.338
514	Punjabi	Asian Indian	63000346	0.333
57	Makrani	HGDP-CEPH	139	0.332
837	Kaingang	Native American	2756	0.332
1272	Cape Mixed Ancestry	African	103206	0.330
1275	Xhosa	African	103287	0.326
1101	Hadza	African	71454	0.322
845	Ticuna (Arara)	Native American	2541	0.316
834	Huilliche	Native American	2122	0.313
1276	Koma	African	103306	0.305
901	Ashkenazi Jewish	Jewish	2315	0.301
1011	Sulka (Watwat)	Pacific Islander	21163	0.299
841	Kogi	Native American	2472	0.298
1107	Burunge	African	70347	0.290
1259	Baluba	African	73014	0.288
1272	Cape Mixed Ancestry	African	103210	0.287
1278	Dogon	African	103343	0.279
1229	Kikuyu	African	72381	0.278

Table S5 Two previously unreported intra-population first-degree relative pairs in the African data set

Population		Identification number		RELPAIR inference: parent/offspring (PO) or full-sibling (FS)	Support for inference: RELPAIR (R) or allele-sharing (A)
ID	Name	First individual	Second individual		
1222	Australian	79193	79195	PO	R,A
1114	Bedzan	71580	71584	FS	R†

†Allele-sharing suggests this pair is a second-degree relative pair (Figure S1). However, in the RELPAIR analysis, the likelihood ratio statistic for all other relative types was <0.0001 for this pair. To be conservative, this pair was treated as a first-degree relative pair when creating the standardized subsets MS5547 and MS5435.

Table S6 12 previously unreported intra-population second-degree relative pairs in the African data set

Population		Identification number		RELPAIR inference: Avuncular (AV), grandparental (GG), or half-sibling (HS)	Support for inference: RELPAIR (R) or allele-sharing (A)
ID	Name	First individual	Second individual		
1132	Podokwo	73033	72886	AV	R,A
1110	Pare	70446	70452	GG	R,A
1114	Bedzan	71578	70672	GG	R,A
1127	Bamoun	71053	71054	GG	R,A
1222	Australian	79194	79195	GG	R,A
1239	Pokot	72632	72636	GG	R,A
1101	Hadza	70030	70027	HS	R,A
1101	Hadza	70047	70038	HS	R,A
1101	Hadza	70048	70071	HS	R,A
1101	Hadza	71474	70079	HS	R,A
1103	Iraqw	70207	70204	HS	R,A
1200	Tikar (South)	71729	71731	HS	R,A

Table S7 Two previously unreported intra-population first-degree relative pairs in the Latino data set

Population		Identification number		RELPAIR inference: parent/offspring (PO) or full-sibling (FS)	Support for inference: RELPAIR (R) or allele-sharing (A)
**ID**	**Name**	**First individual**	**Second individual**		
883	Paposo	2264	2266	PO	R,A
892	Peque	2623	2639	FS	R,A

Table S8 Four previously unreported intra-population second-degree relative pairs in the Latino data set

Population		Identification number		RELPAIR inference: Avuncular (AV), grandparental (GG), or half-sibling (HS)	Support for inference: RELPAIR (R) or allele-sharing (A)
ID	Name	First individual	Second individual		
882	Quetalmahue	2282	2288	AV	R,A
885	Salta	2169	2180	AV	R,A
883	Paposo	2267	2268	HS	R,A
883	Paposo	2267	2274	HS	R,A

Table S9 Six previously unreported intra-population monozygotic pairs in the Pacific Islander data set

Population		Identification number		Support for inference: RELPAIR (R) or allele-sharing (A)
ID	Name	First individual	Second individual	
1040	Micronesians	53071	53141	R,A
1040	Micronesians	53081	53151	R,A
1040	Micronesians	53091	53161	R,A
1040	Micronesians	53101	53181	R,A
1040	Micronesians	53111	53191	R,A
1040	Micronesians	53131	53061	R,A

Table S10 127 previously unreported intra-population first-degree relative pairs in the Pacific Islander data set

Population		Identification number		RELPAIR inference: parent/offspring (PO) or full-sibling (FS)	Support for inference: RELPAIR (R) or allele-sharing (A)
ID	Name	First individual	Second individual		
1001	East Highlands (Gimi & Goroka)	54071	54061	PO	R,A
1001	East Highlands (Gimi & Goroka)	54091	54071	PO	R,A
1005	Anem (Keraiai)	4051	4091	PO	R,A
1005	Anem (Keraiai)	4061	4051	PO	R,A
1005	Anem (Keraiai)	4141	4021	PO	R,A
1006	Anem (Purailing)	5041	5061	PO	R,A
1006	Anem (Purailing)	5053	5061	PO	R,A
1007	Mangseng	12021	12111	PO	R,A
1007	Mangseng	12041	12091	PO	R,A
1008	Melamela	13071	13111	PO	R,A
1010	Sulka (Ganai)	20171	20193	PO	R,A
1010	Sulka (Ganai)	20171	20201	PO	R,A
1012	Kol	8021	8071	PO	R,A
1012	Kol	8113	8051	PO	R,A
1014	Nakanai (Loso)	16001	16041	PO	R,A
1014	Nakanai (Loso)	16131	16061	PO	R,A
1016	Mamusi (Lingite)	11121	11101	PO	R,A
1018	Ata (Lugei)	6013	6123	PO	R,A
1018	Ata (Lugei)	6161	6231	PO	R,A
1020	Baining (Marabu)	18191	18111	PO	R,A
1020	Baining (Marabu)	18221	18153	PO	R,A
1020	Baining (Marabu)	18241	18021	PO	R,A
1020	Baining (Marabu)	18241	18041	PO	R,A
1023	Tolai (Vunairoto)	23212	23214	PO	R,A
1023	Tolai (Vunairoto)	23213	23212	PO	R,A
1024	Mussau	27041	27201	PO	R,A
1024	Mussau	27201	27101	PO	R,A
1024	Mussau	27221	27181	PO	R,A
1025	Lavongai (North)	25033	25001	PO	R,A
1025	Lavongai (North)	25041	25181	PO	R,A
1025	Lavongai (North)	25051	25121	PO	R,A
1026	Lavongai (South)	26141	26154	PO	R,A
1026	Lavongai (South)	26141	26171	PO	R,A
1026	Lavongai (South)	26231	26201	PO	R,A
1028	Nalik	31071	31101	PO	R,A
1028	Nalik	31071	31111	PO	R,A
1028	Nalik	31161	31173	PO	R,A
1028	Nalik	31161	31193	PO	R,A
1029	Notsi	32131	32171	PO	R,A
1029	Notsi	32143	32181	PO	R,A
1030	Kuot (Kabil)	28101	28211	PO	R,A
1032	Madak	30021	30081	PO	R,A
1032	Madak	30053	30221	PO	R,A
1032	Madak	30211	30161	PO	R,A
1034	Teop	35061	35221	PO	R,A

1034	Teop	35071	35001	PO	R,A
1034	Teop	35181	35211	PO	R,A
1034	Teop	35201	35221	PO	R,A
1035	Aita	36061	36121	PO	R,A
1035	Aita	36073	36003	PO	R,A
1035	Aita	36073	36011	PO	R,A
1037	Nasioi	663	52004	PO	R,A
1037	Nasioi	52004	662	PO	R,A
1037	Nasioi	52041	658	PO	R,A
1042	Maoris	62161	62181	PO	R,A
1044	Taruko	42161	42141	PO	R,A
1004	Kove	9201	9241	FS	R,A
1005	Anem (Keraiai)	4101	4013	FS	R,A
1006	Anem (Purailing)	5053	5041	FS	R,A
1006	Anem (Purailing)	5071	5151	FS	R,A
1006	Anem (Purailing)	5181	5101	FS	R,A
1007	Mangseng	12021	12034	FS	R,A
1007	Mangseng	12033	12021	FS	R,A
1007	Mangseng	12033	12034	FS	R,A
1007	Mangseng	12131	12101	FS	R,A
1009	Mengen	14001	14021	FS	R,A
1009	Mengen	14081	14071	FS	R,A
1009	Mengen	14111	14071	FS	R,A
1009	Mengen	14111	14081	FS	R,A
1010	Sulka (Ganai)	20013	20161	FS	R,A
1010	Sulka (Ganai)	20031	20001	FS	R,A
1010	Sulka (Ganai)	20201	20193	FS	R,A
1010	Sulka (Ganai)	20241	20221	FS	R,A
1014	Nakanai (Loso)	16051	16001	FS	R,A
1015	Mamusi (Kisiluvi)	10091	10161	FS	R,A
1015	Mamusi (Kisiluvi)	10241	10111	FS	R,A
1016	Mamusi (Lingite)	11011	11141	FS	R,A
1016	Mamusi (Lingite)	11031	11131	FS	R,A
1016	Mamusi (Lingite)	11051	11011	FS	R,A
1016	Mamusi (Lingite)	11051	11141	FS	R,A
1016	Mamusi (Lingite)	11071	11031	FS	R,A
1016	Mamusi (Lingite)	11071	11131	FS	R,A
1016	Mamusi (Lingite)	11091	11191	FS	R,A
1018	Ata (Lugei)	6003	6013	FS	R,A
1018	Ata (Lugei)	6101	6071	FS	R,A
1018	Ata (Lugei)	6181	6051	FS	R,A
1019	Baining (Malasait)	17011	17211	FS	R,A
1019	Baining (Malasait)	17051	17141	FS	R,A
1019	Baining (Malasait)	17091	17081	FS	R,A
1020	Baining (Marabu)	18041	18021	FS	R,A
1020	Baining (Marabu)	18171	18131	FS	R,A
1020	Baining (Marabu)	18171	18201	FS	R,A
1020	Baining (Marabu)	18201	18131	FS	R,A
1020	Baining (Marabu)	18211	18001	FS	R,A
1021	Baining (Rangulit)	19011	19101	FS	R,A

1021	Baining (Rangulit)	19071	19201	FS	R,A
1021	Baining (Rangulit)	19081	19151	FS	R,A
1022	Tolai (Kabakada)	22113	22091	FS	R,A
1022	Tolai (Kabakada)	22113	22121	FS	R,A
1022	Tolai (Kabakada)	22121	22091	FS	R,A
1022	Tolai (Kabakada)	22191	22211	FS	R,A
1023	Tolai (Vunairoto)	23213	23214	FS	R,A
1024	Mussau	27081	27031	FS	R,A
1026	Lavongai (South)	26171	26154	FS	R,A
1028	Nalik	31101	31111	FS	R,A
1028	Nalik	31134	31031	FS	R,A
1028	Nalik	31144	31121	FS	R,A
1029	Notsi	32061	32081	FS	R,A
1029	Notsi	32061	32091	FS	R,A
1029	Notsi	32091	32081	FS	R,A
1030	Kuot (Kabil)	28031	28021	FS	R,A
1030	Kuot (Kabil)	28091	28073	FS	R,A
1031	Kuot (Lamalaua)	29171	29001	FS	R,A
1032	Madak	30061	30171	FS	R,A
1032	Madak	30061	30221	FS	R,A
1032	Madak	30221	30171	FS	R,A
1033	Saposa	34021	34121	FS	R,A
1035	Aita	36021	36151	FS	R,A
1035	Aita	36031	36081	FS	R,A
1035	Aita	36221	36231	FS	R,A
1037	Nasioi	52004	490	FS	R,A
1037	Nasioi	52041	664	FS	R,A
1042	Maoris	62011	62171	FS	R,A
1042	Maoris	62221	62011	FS	R,A
1042	Maoris	62221	62171	FS	R,A
1043	Ami	47181	47221	FS	R[†]
1043	Ami	47211	47191	FS	R,A

[†]Allele-sharing suggests this pair is a second-degree relative pair (Figure S3). In the RELPAIR analysis, the likelihood ratio statistic for HS was the next highest after FS for this pair. To be conservative, this pair was treated as a first-degree relative pair when creating the standardized subsets MS5547 and MS5435.

Table S11 87 previously unreported intra-population second-degree relative pairs in the Pacific Islander data set

Population		Identification number		RELPAIR inference: Avuncular (AV), grandparental (GG), or half-sibling (HS)	Support for inference: RELPAIR (R) or allele-sharing (A)
ID	Name	First individual	Second individual		
1001	East Highlands (Gimi & Goroka)	54001	54011	AV	R,A
1004	Kove	9111	9221	AV	R,A
1004	Kove	9151	9031	AV	R,A
1007	Mangseng	12053	12041	AV	R,A
1007	Mangseng	12121	12071	AV	R,A
1008	Melamela	13001	13181	AV	R,A
1011	Sulka (Watwat)	21081	21031	AV	R,A
1012	Kol	8171	8161	AV	R,A
1013	Nakanai (Bileki)	15111	15151	AV	R,A
1015	Mamusi (Kisiluvi)	10061	10241	AV	R,A
1015	Mamusi (Kisiluvi)	10211	10121	AV	R,A
1015	Mamusi (Kisiluvi)	10241	10121	AV	R,A
1017	Ata (Uasilau)	7131	7181	AV	R,A
1018	Ata (Lugei)	6003	6123	AV	R,A
1018	Ata (Lugei)	6021	6201	AV	R,A
1019	Baining (Malasait)	17161	17201	AV	R,A
1020	Baining (Marabu)	18031	18131	AV	R,A
1021	Baining (Rangulit)	19001	19021	AV	R,A
1021	Baining (Rangulit)	19111	19081	AV	R,A
1023	Tolai (Vunairoto)	23071	23131	AV	R,A
1025	Lavongai (North)	25041	25001	AV	R,A
1025	Lavongai (North)	25121	25091	AV	R,A
1026	Lavongai (South)	26011	26091	AV	R,A
1028	Nalik	31211	31121	AV	R,A
1030	Kuot (Kabil)	28073	28171	AV	R,A
1030	Kuot (Kabil)	28081	28181	AV	R,A
1030	Kuot (Kabil)	28091	28171	AV	R,A
1031	Kuot (Lamalaua)	29041	29131	AV	R,A
1032	Madak	30191	30104	AV	R,A
1033	Saposa	34011	34041	AV	R,A
1033	Saposa	34033	34151	AV	R,A
1033	Saposa	34111	34231	AV	R,A
1033	Saposa	34171	34191	AV	R,A
1034	Teop	35201	35211	AV	R,A
1035	Aita	36051	36211	AV	R,A
1037	Nasioi	825	823	AV	R,A
1044	Taruko	42151	42201	AV	R,A
1004	Kove	9091	9121	GG	R,A
1005	Anem (Keraiai)	4081	4051	GG	R,A
1005	Anem (Keraiai)	4141	4131	GG	R,A
1006	Anem (Purailing)	5181	5083	GG	R,A
1008	Melamela	13051	13141	GG	R,A
1008	Melamela	13091	13193	GG	R,A
1008	Melamela	13131	13211	GG	R,A
1008	Melamela	13231	13141	GG	R,A

1013	Nakanai (Bileki)	15121	15235	GG	R,A
1013	Nakanai (Bileki)	15161	15121	GG	R,A
1014	Nakanai (Loso)	16051	16023	GG	R,A
1018	Ata (Lugei)	6181	6041	GG	R,A
1020	Baining (Marabu)	18051	18191	GG	R,A
1020	Baining (Marabu)	18171	18031	GG	R,A
1020	Baining (Marabu)	18221	18241	GG	R,A
1021	Baining (Rangulit)	19111	19101	GG	R,A
1027	Tigak	33081	33051	GG	R,A
1028	Nalik	31051	31144	GG	R,A
1031	Kuot (Lamalaua)	29091	29131	GG	R,A
1032	Madak	30081	30141	GG	R,A
1032	Madak	30184	30111	GG	R,A
1034	Teop	35043	35051	GG	R,A
1034	Teop	35201	35061	GG	R,A
1035	Aita	36151	36003	GG	R,A
1035	Aita	36191	36091	GG	R,A
1004	Kove	9014	9133	HS	R,A
1004	Kove	9091	9001	HS	R,A
1004	Kove	9201	9051	HS	R,A
1005	Anem (Keraiai)	4061	4091	HS	R,A
1005	Anem (Keraiai)	4131	4021	HS	R,A
1005	Anem (Keraiai)	4181	4071	HS	R,A
1010	Sulka (Ganai)	20161	20072	HS	R,A
1012	Kol	8001	8081	HS	R,A
1013	Nakanai (Bileki)	15063	15221	HS	R,A
1013	Nakanai (Bileki)	15211	15204	HS	R,A
1015	Mamusi (Kisiluvi)	10121	10111	HS	R,A
1018	Ata (Lugei)	6081	6031	HS	R,A
1022	Tolai (Kabakada)	22231	22051	HS	R,A
1023	Tolai (Vunairoto)	23142	23143	HS	R,A
1026	Lavongai (South)	26121	26091	HS	R,A
1026	Lavongai (South)	26191	26211	HS	R,A
1028	Nalik	31211	31144	HS	R,A
1030	Kuot (Kabil)	28141	28081	HS	R,A
1032	Madak	30081	30171	HS	R,A
1032	Madak	30231	30072	HS	R,A
1033	Saposa	34071	34151	HS	R,A
1034	Teop	35024	35001	HS	R,A
1035	Aita	36003	36161	HS	R,A
1035	Aita	36021	36003	HS	R,A
1044	Taruko	42141	42111	HS	R,A

Table S12 13 previously unreported intra-population parent/parent/offspring trios in the Pacific Islander data set

Population		Identification number			Support for inference: RELPAIR (R) or allele-sharing (A)
ID	Name	Parent 1	Parent 2	Offspring	
1001	East Highlands (Gimi & Goroka)	54061	54091	54071	R,A
1005	Anem (Keraiai)	4061	4091	4051	R,A
1006	Anem (Purailing)	5041	5053	5061	R,A
1010	Sulka (Ganai)	20193	20201	20171	R,A
1020	Baining (Marabu)	18021	18041	18241	R,A
1023	Tolai (Vunairoto)	23213	23214	23212	R,A
1024	Mussau	27041	27101	27201	R,A
1026	Lavongai (South)	26154	26171	26141	R,A
1028	Nalik	31101	31111	31071	R,A
1028	Nalik	31173	31193	31161	R,A
1034	Teop	35061	35201	35221	R,A
1035	Aita	36003	36011	36073	R,A
1037	Nasioi	662	663	52004	R,A

Table S13 One previously unreported inter-population first-degree relative pair in the African data set

First Individual			Second Individual			RELPAIR inference: parent/offspring (PO) or full-sibling (FS)	Support for inference: RELPAIR (R) or allele-sharing (A)
Population		Identification number	Population		Identification number		
ID	Name		ID	Name			
1191	Dinka	71432	1276	Koma	103309	FS	R,A

Table S14 One previously unreported inter-population second-degree relative pair in the African data set

First Individual			Second Individual			RELPAIR inference: Avuncular (AV), grandparental (GG), or half-sibling (HS)	Support for inference: RELPAIR (R) or allele-sharing (A)
Population		Identification number	Population		Identification number		
ID	Name		ID	Name			
1113	Bakola	70618	1115	Mvae	70717	HS	R,A

Table S15 Two previously unreported inter-population monozygotic pairs in the Pacific Islander data set

First individual			Second individual			Support for inference: RELPAIR (R) or allele-sharing (A)
Population		Identification number	Population		Identification number	
ID	Name		ID	Name		
1018	Ata (Lugei)	6021	1026	Lavongai (North)	26051	R,A
1040	Micronesians	53171	1041	Samoans	55001	R,A

Table S16 24 previously unreported inter-population first-degree relative pairs in the Pacific Islander data set

First individual			Second individual			RELPAIR inference: parent/offspring (PO) or full-sibling (FS)	Support for inference: RELPAIR (R) or allele-sharing (A)
Population		Identification number	Population		Identification number		
ID	Name		ID	Name			
1003	Sepik	3091	1026	Lavongai (North)	26163	PO	R,A
1003	Sepik	3191	1029	Madak	32161	PO	R,A
1003	Sepik	3171	1032	Notsi	30201	PO	R,A
1005	Anem (Keraiai)	4021	1006	Anem (Purailing)	5021	PO	R,A
1005	Anem (Keraiai)	4081	1006	Anem (Purailing)	5011	PO	R,A
1005	Anem (Keraiai)	4181	1006	Anem (Purailing)	5111	PO	R,A
1005	Anem (Keraiai)	4201	1006	Anem (Purailing)	5101	PO	R,A
1005	Anem (Keraiai)	4201	1006	Anem (Purailing)	5181	PO	R,A
1010	Sulka (Ganai)	20101	1011	Sulka (Watwat)	21081	PO	R,A
1015	Mamusi (Kisiluvi)	10103	1016	Mamusi (Lingite)	11081	PO	R,A
1022	Tolai (Kabakada)	22131	1023	Tolai (Vunairoto)	23212	PO	R,A
1028	Nalik	31221	1029	Notsi	32021	PO	R,A
1005	Anem (Keraiai)	4114	1006	Anem (Purailing)	5091	FS	R,A
1010	Sulka (Ganai)	20091	1011	Sulka (Watwat)	21101	FS	R,A
1014	Nakanai (Loso)	16061	1018	Ata (Lugei)	6211	FS	R,A
1015	Mamusi (Kisiluvi)	10051	1016	Mamusi (Lingite)	11031	FS	R,A
1015	Mamusi (Kisiluvi)	10051	1016	Mamusi (Lingite)	11071	FS	R,A
1015	Mamusi (Kisiluvi)	10051	1016	Mamusi (Lingite)	11131	FS	R,A
1015	Mamusi (Kisiluvi)	10181	1016	Mamusi (Lingite)	11221	FS	R[†]
1022	Tolai (Kabakada)	22091	1023	Tolai (Vunairoto)	23152	FS	R,A
1022	Tolai (Kabakada)	22113	1023	Tolai (Vunairoto)	23152	FS	R,A
1022	Tolai (Kabakada)	22121	1023	Tolai (Vunairoto)	23152	FS	R,A
1022	Tolai (Kabakada)	22131	1023	Tolai (Vunairoto)	23213	FS	R,A
1022	Tolai (Kabakada)	22131	1023	Tolai (Vunairoto)	23214	FS	R,A

[†]Allele-sharing suggests this pair is a second-degree relative pair (Figure S6). In the RELPAIR analysis, the likelihood ratio statistic for AV was the next highest after FS for this pair. To be conservative, this pair was treated as a first-degree relative pair when creating the MS5795 and MS5879 data sets.

Table S17 54 previously unreported inter-population second-degree relative pairs in the Pacific Islander data set

First individual			Second individual			RELPAIR inference: Avuncular (AV), grandparental (GG), or half-sibling (HS)	Support for inference: RELPAIR (R) or allele-sharing (A)
Population		Identification number	Population		Identification number		
ID	Name			Name			
1005	Anem (Keraiai)	4001	1006	Anem (Purailing)	5031	AV	R,A
1005	Anem (Keraiai)	4051	1006	Anem (Purailing)	5091	AV	R,A
1005	Anem (Keraiai)	4071	1006	Anem (Purailing)	5191	AV	R,A
1005	Anem (Keraiai)	4131	1006	Anem (Purailing)	5021	AV	R,A
1005	Anem (Keraiai)	4181	1006	Anem (Purailing)	5191	AV	R,A
1005	Anem (Keraiai)	4191	1006	Anem (Purailing)	5083	AV	R,A
1005	Anem (Keraiai)	4191	1006	Anem (Purailing)	5101	AV	R,A
1007	Mangseng	12011	1013	Nakanai (Bileki)	15001	AV	R,A
1009	Mengen	14201	1012	Kol	8261	AV	R,A
1010	Sulka (Ganai)	20041	1011	Sulka (Watwat)	21021	AV	R,A
1010	Sulka (Ganai)	20171	1011	Sulka (Watwat)	21021	AV	R,A
1010	Sulka (Ganai)	20131	1027	Tigak	33241	AV	R,A
1014	Nakanai (Loso)	16161	1015	Mamusi (Kisiluvi)	10091	AV	R,A
1014	Nakanai (Loso)	16161	1015	Mamusi (Kisiluvi)	10161	AV	R,A
1014	Nakanai (Loso)	16131	1018	Ata (Lugei)	6211	AV	R,A
1015	Mamusi (Kisiluvi)	10041	1016	Mamusi (Lingite)	11081	AV	R,A
1015	Mamusi (Kisiluvi)	10071	1016	Mamusi (Lingite)	11031	AV	R,A
1015	Mamusi (Kisiluvi)	10071	1016	Mamusi (Lingite)	11071	AV	R,A
1015	Mamusi (Kisiluvi)	10071	1016	Mamusi (Lingite)	11131	AV	R,A
1015	Mamusi (Kisiluvi)	10181	1016	Mamusi (Lingite)	11231	AV	R,A
1015	Mamusi (Kisiluvi)	10191	1016	Mamusi (Lingite)	11021	AV	R,A
1015	Mamusi (Kisiluvi)	10201	1016	Mamusi (Lingite)	11175	AV	R,A
1015	Mamusi (Kisiluvi)	10231	1016	Mamusi (Lingite)	11043	AV	R,A
1018	Ata (Lugei)	6201	1026	Lavongai (South)	26051	AV	R,A
1019	Baining (Malasait)	17001	1021	Baining (Rangulit)	19141	AV	R,A
1019	Baining (Malasait)	17031	1021	Baining (Rangulit)	19181	AV	R,A
1019	Baining (Malasait)	17051	1021	Baining (Rangulit)	19071	AV	R,A
1019	Baining (Malasait)	17051	1021	Baining (Rangulit)	19201	AV	R,A
1019	Baining (Malasait)	17081	1021	Baining (Rangulit)	19171	AV	R,A
1019	Baining (Malasait)	17141	1021	Baining (Rangulit)	19071	AV	R,A

1019	Baining (Malasait)	17141	1021	Baining (Rangulit)	19201	AV	R,A
1019	Baining (Malasait)	17151	1021	Baining (Rangulit)	19201	AV	R,A
1019	Baining (Malasait)	17211	1021	Baining (Rangulit)	19131	AV	R,A
1019	Baining (Malasait)	17221	1021	Baining (Rangulit)	19051	AV	R,A
1019	Baining (Malasait)	17221	1021	Baining (Rangulit)	19181	AV	R,A
1019	Baining (Malasait)	17241	1021	Baining (Rangulit)	19181	AV	R,A
1022	Tolai (Kabakada)	22001	1023	Tolai (Vunairoto)	23171	AV	R,A
1022	Tolai (Kabakada)	22161	1023	Tolai (Vunairoto)	23201	AV	R,A
1022	Tolai (Kabakada)	22211	1023	Tolai (Vunairoto)	23152	AV	R,A
1024	Mussau	27051	1030	Kuot (Kabil)	28201	AV	R,A
1027	Tigak	33021	1029	Notsi	32223	AV	R,A
1027	Tigak	33051	1029	Notsi	32231	AV	R,A
1028	Nalik	31181	1029	Notsi	32021	AV	R,A
1028	Nalik	31121	1030	Kuot (Kabil)	28151	AV	R,A
1028	Nalik	31144	1030	Kuot (Kabil)	28151	AV	R,A
1029	Notsi	32211	1032	Madak	30091	AV	R,A
1005	Anem (Keraiai)	4161	1006	Anem (Purailing)	5061	GG	R,A
1005	Anem (Keraiai)	4181	1006	Anem (Purailing)	5131	GG	R,A
1015	Mamusi (Kisiluvi)	10173	1016	Mamusi (Lingite)	11151	GG	R,A
1026	Lavongai (South)	26081	1030	Kuot (Kabil)	28031	GG	R,A
1005	Anem (Keraiai)	4001	1006	Anem (Purailing)	5141	HS	R,A
1005	Anem (Keraiai)	4191	1006	Anem (Purailing)	5181	HS	R,A
1022	Tolai (Kabakada)	22191	1023	Tolai (Vunairoto)	23152	HS	R,A
1026	Lavongai (South)	26081	1030	Kuot (Kabil)	28021	HS	R,A

Table S18 Three previously unreported inter-population parent/parent/offspring trios in the Pacific Islander data set

Parent 1			Parent 2			Offspring			Support for inference: RELPAIR (R) or allele-sharing (A)
Population		Identification number	Population		Identification number	Population		Identification number	
ID	Name		ID	Name		ID	Name		
1005	Anem (Keraiai)	4141	1006	Anem (Purailing)	5021	1005	Anem (Keraiai)	4021	R,A
1006	Anem (Purailing)	5101	1006	Anem (Purailing)	5181	1005	Anem (Keraiai)	4201	R,A
1022	Tolai (Kabakada)	22131	1023	Tolai (Vunairoto)	23213	1023	Tolai (Vunairoto)	23212	R,A

Table S19 10 previously unreported inter-population second-degree relative pairs in the Native American data set

First individual			Second individual			RELPAIR inference: Avuncular (AV), grandparental (GG), or half-sibling (HS)	Support for inference: RELPAIR (R) or allele-sharing (A)
Population		Identification number	Population		Identification number		
ID	Name		ID	Name			
841	Kogi	2463	849	Arhuaco	2157	AV	R,A
841	Kogi	2463	849	Arhuaco	2158	AV	R,A
841	Kogi	2463	849	Arhuaco	2159	AV	R,A
841	Kogi	2463	849	Arhuaco	2160	AV	R,A
841	Kogi	2463	849	Arhuaco	2419	AV	R,A
841	Kogi	2463	849	Arhuaco	2420	AV	R,A
841	Kogi	2463	849	Arhuaco	2578	AV	R,A
841	Kogi	2463	849	Arhuaco	2732	AV	R,A
845	Ticuna (Arara)	2543	846	Ticuna (Tarapaca)	2794	AV	R,A
845	Ticuna (Arara)	2545	846	Ticuna (Tarapaca)	2794	AV	R,A

Table S20 267 human populations present in the combined data set together with their geographic coordinates, sample sizes, and mean heterozygosities

Population		Location	Geographic region	Latitude [Degrees North]	Longitude [Degrees East]	Sample size			Unbiased heterozygosity		Source
ID	Name					MS5795	MS5547	MS5435	Mean	SD across loci	
20	Orcadian	Orkney Islands	Europe	59	-3	16	15	15	0.724	0.096	[4-6]
21	Adygei	Russia (Caucasus)	Europe	44	39	17	17	17	0.728	0.088	[4-6]
22	Russian	Russia	Europe	61	40	25	25	25	0.731	0.084	[4-6]
24	Basque	France	Europe	43	0	24	24	24	0.718	0.096	[4-6]
25	French	France	Europe	46	2	29	28	28	0.730	0.083	[4-6]
27	Italian	Italy (Bergamo)	Europe	46	10	13	13	13	0.727	0.095	[4-6]
28	Sardinian	Italy	Europe	40	9	28	28	28	0.723	0.086	[4-6]
29	Tuscan	Italy	Europe	43	11	8	8	8	0.735	0.109	[4-6]
34	Mozabite	Algeria (Mzab)	Middle East	32	3	30	29	29	0.738	0.082	[4-6]
36	Bedouin	Israel (Negev)	Middle East	31	35	48	47	46	0.734	0.078	[4-6]
37	Druze	Israel (Carmel)	Middle East	32	35	47	44	42	0.722	0.085	[4-6]
38	Palestinian	Israel (Central)	Middle East	32	35	51	50	46	0.732	0.080	[4-6]
50	Balochi	Pakistan	Central/South Asia	30.49871492	66.5	25	24	24	0.733	0.084	[4-6]
51	Brahui	Pakistan	Central/South Asia	30.49871492	66.5	25	25	25	0.730	0.087	[4-6]
52	Burusho	Pakistan	Central/South Asia	36.49838568	74	25	25	25	0.729	0.085	[4-6]
54	Hazara	Pakistan	Central/South Asia	33.49855601	70	23	22	21	0.728	0.093	[4-6]
56	Kalash	Pakistan	Central/South Asia	35.99366014	71.5	25	24	23	0.698	0.112	[4-6]
57	Makrani	Pakistan	Central/South Asia	26	64	24	24	24	0.737	0.086	[4-6]
58	Pathan	Pakistan	Central/South Asia	33.48700562	70.5	24	24	24	0.734	0.088	[4-6]
59	Sindhi	Pakistan	Central/South Asia	25.49063551	69	25	24	24	0.733	0.084	[4-6]
81[a]	Piapoco	Colombia	America	3	-68	13	7	7	0.625	0.162	[4-6]
82	Karitiana	Brazil	America	-10	-63	24	14	14	0.561	0.178	[4-6]
83	Surui	Brazil	America	-11	-62	21	9	8	0.507	0.203	[4-6]
86	Maya	Mexico	America	19	-91	25	22	21	0.676	0.122	[4-6]
87	Pima	Mexico	America	29	-108	25	14	14	0.617	0.161	[4-6]
430	Bantu (S. Africa)	Southern Africa	Africa	-25.56926433	24.25	8	8	8	0.765	0.103	[4-6]
441	Bantu (Kenya)	Kenya	Africa	-3	37	12	11	11	0.758	0.097	[4-6]
464	Mandenka	Senegal	Africa	12	-12	24	24	22	0.753	0.085	[4-6]
465	Yoruba	Nigeria	Africa	7.995094727	5	25	22	22	0.760	0.083	[4-6]
488	Biaka Pygmy	Central African Republic	Africa	4	17	32	27	23	0.758	0.084	[4-6]
489	Mbuti Pygmy	Congo	Africa	1	29	15	13	13	0.752	0.099	[4-6]
494	San	Namibia	Africa	-21	20	7	6	6	0.745	0.128	[4-6]

601	Han	China	East Asia	32.26566812	114	34	34	34	0.708	0.105	[4-6]
602	Han (N. China)	China	East Asia	32.26566812	114	10	10	10	0.714	0.117	[4-6]
606	Dai	China	East Asia	21	100	10	10	10	0.700	0.133	[4-6]
607	Daur	China	East Asia	48.49753416	124	10	10	10	0.711	0.119	[4-6]
608	Hezhen	China	East Asia	47.4976192	133.5	9	9	9	0.707	0.119	[4-6]
611	Lahu	China	East Asia	22	100	10	8	8	0.688	0.139	[4-6]
612	Miao	China	East Asia	28	109	10	10	10	0.700	0.127	[4-6]
613	Oroqen	China	East Asia	50.43389257	126.5	10	9	9	0.696	0.130	[4-6]
615	She	China	East Asia	27	119	10	10	10	0.694	0.122	[4-6]
616	Tujia	China	East Asia	29	109	10	10	10	0.702	0.127	[4-6]
617	Tu	China	East Asia	36	101	10	10	10	0.707	0.118	[4-6]
618	Xibo	China	East Asia	43.49792973	81.5	9	9	9	0.709	0.126	[4-6]
619	Yi	China	East Asia	28	103	10	10	10	0.709	0.124	[4-6]
622	Mongola	China	East Asia	45	111	10	10	10	0.711	0.122	[4-6]
625	Naxi	China	East Asia	26	100	10	9	9	0.699	0.122	[4-6]
629	Uygur	China	Central/South Asia	44	81	10	10	10	0.733	0.108	[4-6]
677	Cambodian	Cambodia	East Asia	12	105	11	10	10	0.711	0.127	[4-6]
684	Japanese	Japan	East Asia	38	138	29	29	29	0.702	0.113	[4-6]
699	Yakut	Siberia	East Asia	62.98287845	129.5	25	25	25	0.700	0.104	[4-6]
690	Tundra Nentsi	Siberia	East Asia	66.08	76.5	13	13	13	0.716	0.105	[7]
811	Chipewyan	Canada	America	59.55	-107.3	28	24	17	0.671	0.115	[7]
812	Cree	Canada	America	50.33	-102.5	17	16	16	0.695	0.116	[7]
813	Ojibwa	Canada	America	46.5	-81	20	16	12	0.696	0.115	[7]
821	Kaqchikel	Guatemala	America	15	-91	12	11	11	0.665	0.138	[7]
822	Mixtec	Mexico	America	17	-97	19	17	17	0.644	0.143	[7]
823	Mixe	Mexico	America	17	-96	20	20	20	0.641	0.134	[7]
824	Zapotec	Mexico	America	16	-97	17	17	17	0.665	0.140	[7]
831	Guaymi	Panama	America	8.5	-82	16	16	14	0.582	0.176	[7]
832	Cabecar	Costa Rica	America	9.5	-84	20	19	17	0.623	0.147	[7]
833	Aymara	Chile	America	-22	-70	18	18	18	0.660	0.132	[7]
834	Huilliche	Chile	America	-41	-73	19	19	19	0.667	0.120	[7]
835	Guarani	Brazil	America	-23	-54	10	8	8	0.649	0.146	[7]
836	Ache	Paraguay	America	-24	-56	17	16	14	0.483	0.207	[7]
837	Kaingang	Brazil	America	-24	-52.5	5	5	5	0.624	0.189	[7]
838	Quechua	Peru	America	-14	-74	20	20	20	0.669	0.124	[7]
841	Kogi	Colombia	America	11	-74	16	8	7	0.580	0.180	[7]

842 Zenu	Colombia	America	9	-75	18	16	13	0.643	0.143	[7]
843 Inga	Colombia	America	1	-77	16	15	14	0.640	0.140	[7]
844 Wayuu	Colombia	America	11	-73	17	15	15	0.671	0.126	[7]
845 Ticuna (Arara)	Colombia	America	-4	-70	15	14	14	0.590	0.163	[7]
846 Ticuna (Tarapaca)	Colombia	America	-4	-70	18	12	10	0.587	0.175	[7]
847 Embera	Colombia	America	7	-76	11	7	7	0.625	0.158	[7]
848 Waunana	Colombia	America	5	-77	20	12	11	0.625	0.160	[7]
849 Arhuaco	Colombia	America	11	-73.8	16	9	9	0.633	0.148	[7]
871 Oriente	Guatemala	Latino	14.633333	-90.516667	19	19	19	0.727	0.085	[8]
872 Mexico City	Mexico	Latino	19.4	-99.2	19	19	19	0.732	0.088	[8]
881 CVCR	Costa Rica	Latino	9.933333	-84.083333	20	20	20	0.736	0.084	[8]
882 Quetalmahue	Chile	Latino	-41.466667	-73.533333	20	20	19	0.703	0.103	[8]
883 Paposo	Chile	Latino	-25.05	-70.266667	20	19	18	0.710	0.099	[8]
884 Catamarca	Argentina	Latino	-28.466667	-65.783333	12	12	12	0.723	0.101	[8]
885 Salta	Argentina	Latino	-24.783333	-65.416667	19	19	18	0.703	0.107	[8]
886 Tucuman	Argentina	Latino	-26.816667	-65.216667	19	19	19	0.737	0.086	[8]
887 RGS	Brazil	Latino	-31	-54	20	20	20	0.744	0.076	[8]
891 Pasto	Colombia	Latino	1.216667	-77.283333	19	19	19	0.718	0.092	[8]
892 Peque	Colombia	Latino	7.316667	-75.833333	20	19	19	0.708	0.099	[8]
893 Medellin	Colombia	Latino	6.25	-75.583333	20	20	20	0.738	0.082	[8]
894 Cundinamarca	Colombia	Latino	4.6	-74.083333	19	19	19	0.721	0.096	[8]
901[b] Ashkenazi Jewish	Israel (Ashkelon)	Europe	31.666667	34.566667	19	19	19	0.732	0.088	[9]
902[b] Moroccan Jewish	Israel (Ashkelon)	Middle East	31.666667	34.566667	20	20	20	0.728	0.089	[9]
903[b] Tunisian Jewish	Israel (Ashkelon)	Middle East	31.666667	34.566667	20	20	18	0.722	0.095	[9]
904[b] Turkish Jewish	Israel (Ashkelon)	Middle East	31.666667	34.566667	20	20	20	0.735	0.084	[9]
501 Assamese	India	Central/South Asia	26	93	25	25	25	0.732	0.086	[10]
502 Bengali	India	Central/South Asia	23.25766454	87.66666667	27	27	27	0.732	0.087	[10]
504[c] Gujarati	India	Central/South Asia	23	72	252	234	229	0.725	0.083	[10, 11]
505 Hindi	India	Central/South Asia	26.98091726	78.39357143	28	28	28	0.729	0.087	[10]
506 Kannada	India	Central/South Asia	15	75	24	24	24	0.731	0.088	[10]
507 Kashmiri	India	Central/South Asia	32.44	74.54	25	25	25	0.735	0.087	[10]
508 Konkani	India	Central/South Asia	14.57081522	75.13095238	42	42	42	0.726	0.083	[10]
509 Malayalam	India	Central/South Asia	10	76.25	25	25	25	0.729	0.091	[10]
510 Marathi	India	Central/South Asia	19.81684261	75.94230769	26	26	26	0.734	0.084	[10]
511 Marwari	India	Central/South Asia	26.67171131	74.264	25	25	25	0.725	0.092	[10]
512 Oriya	India	Central/South Asia	20	85	26	26	26	0.728	0.088	[10]

513	Parsi	India	Central/South Asia	19	72.8	25	25	25	0.724	0.090	[10]
514	Punjabi	India	Central/South Asia	30.47159766	75.29071429	27	27	27	0.732	0.085	[10]
515	Tamil	India	Central/South Asia	11.10167224	77.94827586	29	29	29	0.732	0.087	[10]
516	Telugu	India	Central/South Asia	15.9257581	79.62962963	27	27	27	0.727	0.091	[10]
1001[d]	East Highlands (Gimi & Goroka)	New Guinea	Oceania	-6.083	145.4	29	28	27	0.677	0.134	[2]
1003	Sepik	New Guinea	Oceania	-3.583	143.333	20	20	20	0.682	0.134	[2]
1004	Kove	New Britain	Oceania	-5.4667	148.95	25	24	20	0.677	0.125	[2]
1005	Anem (Keraiai)	New Britain	Oceania	-5.45	148.9833	16	14	11	0.684	0.128	[2]
1006	Anem (Purailing)	New Britain	Oceania	-5.45	148.984	17	14	14	0.674	0.136	[2]
1007	Mangseng	New Britain	Oceania	-5.933	150.7	20	16	15	0.697	0.121	[2]
1008	Melamela	New Britain	Oceania	-5	151.25	25	24	20	0.685	0.119	[2]
1009	Mengen	New Britain	Oceania	-5.1	151.4	23	20	20	0.688	0.129	[2]
1010	Sulka (Ganai)	New Britain	Oceania	-4.5	152.333	22	17	17	0.689	0.128	[2]
1011	Sulka (Watwat)	New Britain	Oceania	-4.483	152.3	15	15	14	0.686	0.136	[2]
1012	Kol	New Britain	Oceania	-5.383	151.633	20	18	16	0.672	0.135	[2]
1013	Nakanai (Bileki)	New Britain	Oceania	-5.75	150.8	24	24	20	0.686	0.123	[2]
1014	Nakanai (Loso)	New Britain	Oceania	-5.483	150.8	17	16	15	0.657	0.135	[2]
1015	Mamusi (Kisiluvi)	New Britain	Oceania	-5.7333	151.0833	22	20	19	0.655	0.138	[2]
1016	Mamusi (Lingite)	New Britain	Oceania	-5.867	151.1	20	16	16	0.646	0.148	[2]
1017	Ata (Uasilau)	New Britain	Oceania	-5.683	151	24	24	23	0.649	0.136	[2]
1018	Ata (Lugei)	New Britain	Oceania	-5.6	151	23	18	17	0.661	0.130	[2]
1019	Baining (Malasait)	New Britain	Oceania	-4.467	151.9	25	22	21	0.620	0.155	[2]
1020	Baining (Marabu)	New Britain	Oceania	-4.633	152.3	25	18	18	0.624	0.156	[2]
1021	Baining (Rangulit)	New Britain	Oceania	-4.4	151.9	22	19	18	0.628	0.151	[2]
1022	Tolai (Kabakada)	New Britain	Oceania	-4.483	152.1	18	17	16	0.684	0.132	[2]
1023	Tolai (Vunairoto)	New Britain	Oceania	-4.2	152.1	21	21	19	0.690	0.124	[2]
1024	Mussau	Mussau	Oceania	-1.583	149.733	25	22	22	0.691	0.121	[2]
1025	Lavongai (North)	New Hanover	Oceania	-2.433	150.35	24	21	21	0.686	0.122	[2]
1026	Lavongai (South)	New Hanover	Oceania	-2.5667	150.4333	22	19	17	0.688	0.126	[2]
1027	Tigak	New Ireland	Oceania	-2.6	150.87	23	23	22	0.691	0.120	[2]
1028	Nalik	New Ireland	Oceania	-2.983	151.52	24	19	18	0.693	0.122	[2]
1029	Notsi	New Ireland	Oceania	-3.05	151.65	23	19	19	0.690	0.126	[2]
1030	Kuot (Kabil)	New Ireland	Oceania	-3.067	151.7	25	22	20	0.684	0.124	[2]
1031	Kuot (Lamalaua)	New Ireland	Oceania	-3	151.5	18	17	16	0.690	0.128	[2]
1032	Madak	New Ireland	Oceania	-3.1	151.7	24	20	17	0.687	0.126	[2]

1033	Saposa	Bougainville	Oceania	-5.5833	154.67	25	24	20	0.685	0.126	[2]
1034	Teop	Bougainville	Oceania	-5.85	155.18	23	19	18	0.690	0.128	[2]
1035	Aita	Bougainville	Oceania	-5.9	155.083	25	19	17	0.643	0.143	[2]
1037[e]	Nasioi	Bougainville	Oceania	-6.483	155.833	23	14	12	0.667	0.147	[2]
1040	Micronesians	Micronesia	Oceania	12.5	150	16	16	16	0.693	0.123	[2]
1041	Samoans	Polynesia	Oceania	-13.35	-172.2	10	10	10	0.680	0.142	[2]
1042	Maoris	Polynesia	Oceania	-41	174	30	27	27	0.662	0.122	[2]
1043	Ami	Taiwan	East Asia	23.3	121	25	23	23	0.672	0.126	[2]
1044	Taruko	Taiwan	East Asia	23.3	121	25	24	23	0.653	0.142	[2]
1101	Hadza	Tanzania	Africa	-3.8	35.3	61	61	57	0.730	0.092	[3]
1102	Sandawe	Tanzania	Africa	-5.5	35.5	51	51	51	0.763	0.074	[3]
1103	Iraqw	Tanzania	Africa	-4	35.5	46	46	45	0.758	0.073	[3]
1104	Turu	Tanzania	Africa	-5	35	32	32	32	0.768	0.072	[3]
1105	Mbugwe	Tanzania	Africa	-3.8	35.8	21	21	21	0.765	0.082	[3]
1106	Rangi	Tanzania	Africa	-5	36	36	36	36	0.765	0.077	[3]
1107	Burunge	Tanzania	Africa	-5.3	36	21	21	21	0.767	0.082	[3]
1108	Maasai (Tanzania)	Tanzania	Africa	-4	37	36	36	36	0.767	0.072	[3]
1109	Akie	Tanzania	Africa	-5	37.5	23	23	23	0.756	0.087	[3]
1110	Pare	Tanzania	Africa	-4.5	38	23	23	22	0.762	0.082	[3]
1111	Mbugu	Tanzania	Africa	-4.8	38.5	22	22	22	0.750	0.084	[3]
1112	Baka	Cameroon	Africa	2.5	13.5	48	48	48	0.761	0.083	[3]
1113	Bakola	Cameroon	Africa	2.8	10	42	42	42	0.760	0.082	[3]
1114	Bedzan	Cameroon	Africa	5.5	11.6	17	16	15	0.756	0.094	[3]
1115	Mvae	Cameroon	Africa	3	12	24	24	24	0.764	0.081	[3]
1116	Ngumba	Cameroon	Africa	3	10.3	26	26	26	0.762	0.083	[3]
1117	Fulani (Adamawa)	Cameroon	Africa	9	13.5	41	41	41	0.755	0.078	[3]
1118	Kanuri	Cameroon	Africa	11.3	14.3	31	31	31	0.760	0.079	[3]
1119	Mada	Cameroon	Africa	10.8	14.1	28	28	28	0.754	0.082	[3]
1120	Zime	Cameroon	Africa	9	14.5	30	30	30	0.763	0.082	[3]
1121	Ouldeme	Cameroon	Africa	11	14.3	26	26	26	0.753	0.085	[3]
1122	Giziga	Cameroon	Africa	10.3	14.3	24	24	24	0.756	0.086	[3]
1123	Mandara	Cameroon	Africa	11.3	14	26	26	26	0.756	0.080	[3]
1124	Baggara	Cameroon	Africa	12.5	14.5	23	23	23	0.758	0.079	[3]
1125	Kotoko	Cameroon	Africa	11.8	14.8	17	17	17	0.756	0.086	[3]
1126	Zulgo	Cameroon	Africa	10.8	14	22	22	22	0.751	0.087	[3]
1127	Bamoun	Cameroon	Africa	5.5	10.8	31	31	30	0.760	0.081	[3]

1128	Banen	Cameroon	Africa	4.8	10.8	25	25	25	0.760	0.084	[3]
1129	Bafia	Cameroon	Africa	4.8	11	30	30	30	0.762	0.082	[3]
1130	Lemande	Cameroon	Africa	4.5	11	26	26	26	0.758	0.083	[3]
1131	Batanga	Cameroon	Africa	3	10	20	20	20	0.757	0.089	[3]
1132	Podokwo	Cameroon	Africa	11	12.1	30	30	29	0.751	0.084	[3]
1159	Yoruba	Nigeria	Africa	8	4	25	25	25	0.756	0.086	[3]
1185	Bassange	Nigeria	Africa	9	5.5	20	20	20	0.754	0.090	[3]
1186	Igbo	Nigeria	Africa	6	7	28	28	28	0.760	0.084	[3]
1187	Igala	Nigeria	Africa	7	7	17	17	17	0.758	0.084	[3]
1188	Gwari	Nigeria	Africa	10	7	22	22	22	0.758	0.086	[3]
1189	Hausa (Nigeria)	Nigeria	Africa	12	8	16	16	16	0.755	0.093	[3]
1190	Beja (Banuamir)	Sudan	Middle East	21	36	23	23	23	0.752	0.080	[3]
1191	Dinka	Sudan	Africa	8	30	16	16	16	0.750	0.090	[3]
1192	Datoga	Tanzania	Africa	-4.5	35.5	54	54	54	0.760	0.072	[3]
1193	Sukuma	Tanzania	Africa	-3	33.5	10	10	10	0.766	0.090	[3]
1194	Gogo	Tanzania	Africa	-6	36	13	13	13	0.767	0.084	[3]
1195	Fiome (Gorowa)	Tanzania	Africa	-4.3	35.8	22	22	22	0.762	0.081	[3]
1196	Iyassa	Cameroon	Africa	2.5	9.8	37	37	37	0.761	0.081	[3]
1197	Fang	Cameroon	Africa	2.5	13	19	19	19	0.759	0.087	[3]
1198	Mabea	Cameroon	Africa	2.9	10.3	13	13	13	0.761	0.090	[3]
1199	Yambassa	Cameroon	Africa	4.8	11.3	17	17	17	0.758	0.090	[3]
1200	Tikar (South)	Cameroon	Africa	5.5	11.5	21	21	20	0.763	0.085	[3]
1201	Tikar (North)	Cameroon	Africa	6.3	11.5	13	13	13	0.759	0.092	[3]
1202	Ntumu	Cameroon	Africa	2.3	10.5	11	11	11	0.762	0.091	[3]
1203	Massa	Cameroon	Africa	10.3	15.3	15	15	15	0.754	0.093	[3]
1204	Tupuri	Cameroon	Africa	10.3	14.8	22	22	22	0.757	0.086	[3]
1205	Bulu	Cameroon	Africa	3	11	22	22	22	0.765	0.081	[3]
1206	Ashanti	Ghana	Africa	6	-1	15	15	15	0.759	0.092	[3]
1207	Brong	Ghana	Africa	7.5	-2	26	26	26	0.757	0.084	[3]
1209[b]	Chicago	USA	Afro-European	41.881944	-87.627778	15	15	15	0.766	0.082	[3]
1210[b]	Pittsburgh	USA	Afro-European	40.441667	-80	21	21	21	0.766	0.079	[3]
1211[b]	Baltimore	USA	Afro-European	39.283333	-76.616667	44	44	44	0.767	0.073	[3]
1212	North Carolina	USA	Afro-European	-	-	18	18	18	0.768	0.080	[3]
1214	Maasai (Mumonyot)	Kenya	Africa	0.6	37	12	12	12	0.759	0.083	[3]
1215	Maasai (Ilgwesi)	Kenya	Africa	0.3	36.8	21	21	21	0.758	0.082	[3]
1216	Samburu	Kenya	Africa	1.5	37	18	18	18	0.763	0.082	[3]

1217	Yaaku	Kenya	Africa	0.5	37	19	19	19	0.750	0.082	[3]
1219	Sambaa	Tanzania	Africa	-4.5	38.3	18	18	18	0.760	0.085	[3]
1220	Dorobo	Tanzania	Africa	-5	37	10	10	10	0.759	0.097	[3]
1222	Australian	Australia	Oceania	-	-	10	9	9	0.719	0.121	[3]
1223	Beja (Hadandawa)	Sudan	Middle East	21	36	19	19	19	0.745	0.084	[3]
1224	Tugen	Kenya	Africa	0.8	35.8	22	22	22	0.757	0.077	[3]
1225	Burji	Ethiopia	Africa	5.5	37.8	24	24	24	0.763	0.076	[3]
1226	Marakwet	Kenya	Africa	1.3	35.5	14	14	14	0.754	0.094	[3]
1227	Luhya	Kenya	Africa	0.5	34.5	17	17	17	0.763	0.086	[3]
1228	Luo	Kenya	Africa	-0.5	34.5	28	28	28	0.763	0.082	[3]
1229	Kikuyu	Kenya	Africa	-1	37	21	21	21	0.764	0.080	[3]
1230	Sengwer	Kenya	Africa	1	35	21	21	21	0.754	0.079	[3]
1231	Okiek	Kenya	Africa	0.3	36	22	22	22	0.746	0.085	[3]
1232	Wata	Kenya	Africa	3.5	37	6	6	6	0.762	0.105	[3]
1233	Nandi	Kenya	Africa	0	35.5	11	11	11	0.761	0.094	[3]
1234	El Molo	Kenya	Africa	2.8	36.8	16	16	16	0.749	0.088	[3]
1235	Gabra	Kenya	Africa	3	37.5	16	16	16	0.755	0.083	[3]
1236	Sabaot	Kenya	Africa	1	34.8	20	20	20	0.762	0.079	[3]
1237	Rendille	Kenya	Africa	2.3	37.5	28	28	28	0.755	0.075	[3]
1238	Turkana	Kenya	Africa	3	36	26	26	26	0.760	0.081	[3]
1239	Pokot	Kenya	Africa	1.5	35.5	23	23	22	0.755	0.082	[3]
1240	Borana	Kenya	Africa	3	38	32	32	32	0.760	0.078	[3]
1241	Maasai (Ilchamus)	Kenya	Africa	1.5	37.1	26	26	26	0.759	0.079	[3]
1242	Konso	Ethiopia	Africa	5.5	37.5	14	14	14	0.762	0.090	[3]
1243	Laka	Chad	Africa	8	16	33	33	33	0.760	0.084	[3]
1244	Ngambaye	Chad	Africa	9	16	30	30	30	0.758	0.082	[3]
1245	Kaba	Chad	Africa	8	16.8	27	27	27	0.760	0.080	[3]
1246	Bulala	Chad	Africa	13	18	15	15	15	0.754	0.089	[3]
1247	Kanembou	Chad	Africa	14	15	5	5	5	0.756	0.119	[3]
1248	Mbum	Central African Republic	Africa	5.5	13.5	13	13	13	0.755	0.099	[3]
1249	Tutsi/Hutu	Rwanda	Africa	-2	30	8	8	8	0.764	0.097	[3]
1250	Yakoma	Central African Republic	Africa	4.3	22.3	6	6	6	0.759	0.121	[3]
1252	Gbaya	Central African Republic	Africa	5	15	15	15	15	0.760	0.085	[3]
1253	Fulani (Mbororo)	Cameroon	Africa	11.8	14.8	13	13	13	0.737	0.093	[3]
1254	Wimbum	Cameroon	Africa	6.5	10.8	14	14	14	0.756	0.095	[3]
1255	Batie	Cameroon	Africa	4.3	11	16	16	16	0.760	0.093	[3]

1256	Hausa (Cameroon)	Cameroon	Africa	10.5	14.5	27	27	27	0.760	0.083	[3]
1257	Kongo	Democratic Republic Congo	Africa	-5.5	15	17	17	17	0.759	0.088	[3]
1258	Barega	Democratic Republic Congo	Africa	-3.5	28	4	4	4	0.763	0.135	[3]
1259	Baluba	Democratic Republic Congo	Africa	-9	25	5	5	5	0.757	0.130	[3]
1262	Dioula	Ivory Coast	Africa	9.5	-4.5	5	5	5	0.756	0.119	[3]
1266	Ewondo	Cameroon	Africa	3.8	11.5	3	3	3	0.760	0.163	[3]
1267	Eton	Cameroon	Africa	4.3	11.5	4	4	4	0.764	0.130	[3]
1268	Nuer	Sudan	Africa	8.5	31	17	17	17	0.750	0.087	[3]
1269	Shilook	Sudan	Africa	10	32	15	15	15	0.751	0.094	[3]
1270	Nyimang	Sudan	Africa	12.3	29.5	12	12	12	0.750	0.095	[3]
1271	Sara (various)	Chad	Africa	8	17.5	26	26	26	0.759	0.084	[3]
1272	Cape Mixed Ancestry	South Africa (Cape Town)	Afro-European	-34	18.5	33	33	33	0.767	0.074	[3]
1273	Venda	South Africa	Africa	-22.5	30	11	11	11	0.752	0.096	[3]
1274	!Xun/Kxoe	South Africa	Africa	-30	18	6	6	6	0.757	0.129	[3]
1275	Xhosa	South Africa	Africa	-32	28	27	27	27	0.762	0.086	[3]
1276	Koma	Nigeria	Africa	8.5	12.7	9	9	9	0.743	0.113	[3]
1277	Beta Israel	Ethiopia	Africa	12	38	17	17	17	0.753	0.081	[3]
1278	Dogon	Mali	Africa	14	-3	3	3	3	0.371	0.305	[3]
1280	Temani	Yemen	Middle East	15	45	18	18	18	0.724	0.093	[3]
1281	Fulani (Nigeria)	Nigeria	Africa	11	11	4	4	4	0.757	0.138	[3]

[a]Referred to as 'Colombian' in Rosenberg *et al*. [6], Rosenberg *et al*. [5], and Ramachandran *et al*. [4].

[b]The geographic coordinates of the city where sampling took place are provided.

[c]This population includes the Gujarati individuals studied by Pemberton *et al*. [11].

[d]This population includes the individuals from the HGDP-CEPH Papuan population.

[e]This population includes the individuals from the HGDP-CEPH Melanesian population.

Table S21 The 248 individuals in MS5795 but not in MS5547

Population		Identification number
ID	Name	
20	Orcadian	801
25	French	532
34	Mozabite	1281
36	Bedouin	633
37	Druze	592
37	Druze	603
37	Druze	605
38	Palestinian	695
50	Balochi	84
54	Hazara	113
56	Kalash	292
59	Sindhi	203
81	Piapoco	705
81	Piapoco	707
81	Piapoco	709
81	Piapoco	792
81	Piapoco	793
81	Piapoco	827
82	Karitiana	996
82	Karitiana	997
82	Karitiana	1000
82	Karitiana	1004
82	Karitiana	1005
82	Karitiana	1007
82	Karitiana	1008
82	Karitiana	1011
82	Karitiana	1016
82	Karitiana	1017
83	Surui	830
83	Surui	834
83	Surui	835
83	Surui	839
83	Surui	840
83	Surui	841
83	Surui	842
83	Surui	844
83	Surui	847
83	Surui	848
83	Surui	850
83	Surui	851
86	Maya	866
86	Maya	867
86	Maya	878
87	Pima	1038
87	Pima	1039
87	Pima	1040
87	Pima	1042
87	Pima	1045
87	Pima	1046
87	Pima	1049
87	Pima	1052
87	Pima	1054
87	Pima	1061
441	Bantu (Kenya)	1413
465	Yoruba	921
465	Yoruba	922
465	Yoruba	923
488	Biaka Pygmy	451
488	Biaka Pygmy	477
488	Biaka Pygmy	1088
488	Biaka Pygmy	1089
488	Biaka Pygmy	1093
489	Mbuti Pygmy	468
489	Mbuti Pygmy	983
494	San	988
504	Gujarati	4250001
504	Gujarati	46500080
504	Gujarati	48900024
504	Gujarati	48900027
504	Gujarati	425000471
504	Gujarati	425000474
504	Gujarati	425000475
504	Gujarati	425000476
504	Gujarati	425000477
504	Gujarati	425000478
504	Gujarati	425000480
504	Gujarati	425000481
504	Gujarati	425000482
504	Gujarati	425000485
504	Gujarati	425000489
504	Gujarati	425000501
504	Gujarati	425000502
504	Gujarati	506000139
611	Lahu	1324
611	Lahu	1325
613	Oroqen	1210
625	Naxi	1343
677	Cambodian	718
811	Chipewyan	2395
811	Chipewyan	2399
811	Chipewyan	2400
811	Chipewyan	2560
812	Cree	2418
813	Ojibwa	2421
813	Ojibwa	2430
813	Ojibwa	2432
813	Ojibwa	2440
821	Kaqchikel	2690

87	Pima	1048	822	Mixtec	2054
822	Mixtec	2055	1007	Mangseng	12034
832	Cabecar	2034	1007	Mangseng	12041
835	Guarani	2726	1007	Mangseng	12131
835	Guarani	2729	1008	Melamela	13111
836	Ache	2746	1009	Mengen	14021
841	Kogi	2461	1009	Mengen	14081
841	Kogi	2462	1009	Mengen	14111
841	Kogi	2463	1010	Sulka (Ganai)	20001
841	Kogi	2464	1010	Sulka (Ganai)	20161
841	Kogi	2467	1010	Sulka (Ganai)	20171
841	Kogi	2473	1010	Sulka (Ganai)	20201
841	Kogi	2475	1010	Sulka (Ganai)	20241
841	Kogi	2476	1012	Kol	8021
842	Zenu	2485	1012	Kol	8051
842	Zenu	2487	1014	Nakanai (Loso)	16001
843	Inga	2518	1015	Mamusi (Kisiluvi)	10161
844	Wayuu	2525	1015	Mamusi (Kisiluvi)	10241
844	Wayuu	2539	1016	Mamusi (Lingite)	11011
845	Ticuna (Arara)	2543	1016	Mamusi (Lingite)	11051
846	Ticuna (Tarapaca)	2764	1016	Mamusi (Lingite)	11121
846	Ticuna (Tarapaca)	2767	1016	Mamusi (Lingite)	11191
846	Ticuna (Tarapaca)	2768	1018	Ata (Lugei)	6003
846	Ticuna (Tarapaca)	2793	1018	Ata (Lugei)	6101
846	Ticuna (Tarapaca)	2795	1018	Ata (Lugei)	6123
846	Ticuna (Tarapaca)	2797	1018	Ata (Lugei)	6161
847	Embera	2562	1018	Ata (Lugei)	6181
847	Embera	2564	1019	Baining (Malasait)	17051
847	Embera	2566	1019	Baining (Malasait)	17081
847	Embera	2569	1019	Baining (Malasait)	17211
848	Waunana	2584	1020	Baining (Marabu)	18001
848	Waunana	2585	1020	Baining (Marabu)	18021
848	Waunana	2586	1020	Baining (Marabu)	18131
848	Waunana	2587	1020	Baining (Marabu)	18171
848	Waunana	2588	1020	Baining (Marabu)	18191
848	Waunana	2590	1020	Baining (Marabu)	18221
848	Waunana	2597	1020	Baining (Marabu)	18241
848	Waunana	2600	1021	Baining (Rangulit)	19071
849	Arhuaco	2158	1021	Baining (Rangulit)	19081
849	Arhuaco	2159	1021	Baining (Rangulit)	19101
849	Arhuaco	2458	1022	Tolai (Kabakada)	22191
849	Arhuaco	2460	1024	Mussau	27081
849	Arhuaco	2576	1024	Mussau	27181
849	Arhuaco	2577	1024	Mussau	27201
849	Arhuaco	2579	1025	Lavongai (North)	25001
883	Paposo	2266	1025	Lavongai (North)	25121
892	Peque	2623	1025	Lavongai (North)	25181
1001	East Highlands	54071	1026	Lavongai (North)	26141
1004	Kove	9201	1026	Lavongai (North)	26171
1005	Anem (Keraiai)	4051	1026	Lavongai (North)	26231
1005	Anem (Keraiai)	4101	1028	Nalik	31031
1006	Anem (Purailing)	5041	1028	Nalik	31071
1006	Anem (Purailing)	5053	1028	Nalik	31101
1006	Anem (Purailing)	5151	1028	Nalik	31144

1007	Mangseng	12021	1028	Nalik	31161
1029	Notsi	32061	1035	Aita	36121
1029	Notsi	32091	1035	Aita	36151
1029	Notsi	32171	1035	Aita	36231
1029	Notsi	32181	1037	Nasioi	490
1030	Kuot (Kabil)	28021	1037	Nasioi	657
1030	Kuot (Kabil)	28091	1037	Nasioi	658
1030	Kuot (Kabil)	28101	1037	Nasioi	662
1031	Kuot (Lamalaua)	29001	1037	Nasioi	663
1032	Madak	30081	1037	Nasioi	664
1032	Madak	30161	1037	Nasioi	789
1032	Madak	30171	1037	Nasioi	824
1032	Madak	30221	1037	Nasioi	825
1033	Saposa	34021	1042	Maoris	62171
1034	Teop	35001	1042	Maoris	62181
1034	Teop	35201	1042	Maoris	62221
1034	Teop	35211	1043	Ami	47191
1034	Teop	35221	1043	Ami	47221
1035	Aita	36003	1044	Taruko	42141
1035	Aita	36011	1114	Bedzan	71584
1035	Aita	36031	1222	Australian	79195

Table S22 The 112 individuals in MS5547 but not in MS5435

Population		Identification number
ID	Name	
36	Bedouin	617
37	Druze	570
37	Druze	585
38	Palestinian	681
38	Palestinian	728
38	Palestinian	742
38	Palestinian	743
54	Hazara	128
56	Kalash	321
83	Surui	833
86	Maya	874
464	Mandenka	916
464	Mandenka	919
488	Biaka Pygmy	448
488	Biaka Pygmy	1084
488	Biaka Pygmy	1085
488	Biaka Pygmy	1091
504	Gujarati	42600087
504	Gujarati	50200040
504	Gujarati	51300078
504	Gujarati	404000112
504	Gujarati	503000106
811	Chipewyan	2156
811	Chipewyan	2383
811	Chipewyan	2387
811	Chipewyan	2390
811	Chipewyan	2393
811	Chipewyan	2515
811	Chipewyan	2800
813	Ojibwa	2428
813	Ojibwa	2436
813	Ojibwa	2437
813	Ojibwa	2438
831	Guaymi	2002
831	Guaymi	2014
832	Cabecar	2026
832	Cabecar	2037
836	Ache	2747
836	Ache	2748
841	Kogi	2466
842	Zenu	2494
842	Zenu	2496
842	Zenu	2499
843	Inga	2505
846	Ticuna (Tarapaca)	2761
846	Ticuna (Tarapaca)	2792
848	Waunana	2599
882	Quetalmahue	2288
883	Paposo	2267
903	Tunisian Jewish	2345
903	Tunisian Jewish	2348
1001	East Highlands	54011
1004	Kove	9091
1004	Kove	9111
1004	Kove	9133
1004	Kove	9151
1005	Anem (Keraiai)	4071
1005	Anem (Keraiai)	4091
1005	Anem (Keraiai)	4141
1007	Mangseng	12071
1008	Melamela	13001
1008	Melamela	13091
1008	Melamela	13131
1008	Melamela	13141
1011	Sulka (Watwat)	21031
1012	Kol	8081
1012	Kol	8171
1013	Nakanai (Bileki)	15063
1013	Nakanai (Bileki)	15121
1013	Nakanai (Bileki)	15151
1013	Nakanai (Bileki)	15204
1014	Nakanai (Loso)	16023
1015	Mamusi (Kisiluvi)	10121
1017	Ata (Uasilau)	7181
1018	Ata (Lugei)	6081
1019	Baining (Malasait)	17201
1021	Baining (Rangulit)	19001
1022	Tolai (Kabakada)	22051
1023	Tolai (Vunairoto)	23131
1023	Tolai (Vunairoto)	23143
1026	Lavongai (South)	26091
1026	Lavongai (South)	26211
1027	Tigak	33081
1028	Nalik	31211
1030	Kuot (Kabil)	28081
1030	Kuot (Kabil)	28171
1031	Kuot (Lamalaua)	29131
1032	Madak	30072
1032	Madak	30104
1032	Madak	30111
1033	Saposa	34011
1033	Saposa	34151
1033	Saposa	34171
1033	Saposa	34231
1034	Teop	35043
1035	Aita	36091
1035	Aita	36211
1037	Nasioi	823

885	Salta	2180	1037	Nasioi	978
1044	Taruko	42151	1110	Pare	70446
1101	Hadza	70027	1114	Bedzan	71578
1101	Hadza	70047	1127	Bamoun	71054
1101	Hadza	70048	1132	Podokwo	73033
1101	Hadza	71474	1200	Tikar (South)	71731
1103	Iraqw	70204	1239	Pokot	72636

Table S23 Six chimpanzee populations present in the chimpanzee data set together with their sample sizes and average heterozygosities in the combined human-chimpanzee data set

**Population**		**Reported category in Becquet *et al.***	**Sample size**			**Unbiased heterozygosity**		**Source**
**ID**	**Species**		**MS5879**	**MS5631**	**MS5519**	**Mean**	**SD across loci**	
1	Chimpanzee	Central	16	16	16	0.738	0.212	[12]
2	Chimpanzee	Eastern	7	7	7	0.698	0.229	[12]
3	Chimpanzee	Western	41	41	41	0.627	0.239	[12]
4	Chimpanzee	Unreported	11	11	11	0.644	0.245	[12]
5	Chimpanzee	Hybrid	3	3	3	0.746	0.238	[12]
6	Bonobo	Bonobo	6	6	6	0.656	0.271	[12]

Population IDs are the same as those used in Becquet *et al*. [12].

Table S24 Three individuals with >27.5% missing data in the combined human-chimpanzee data set

Population			Identification number of individual	Fraction of loci with missing genotypes
ID	Name	Data set of origin		
1270	Nyimang	African	103351	0.382
886	Tucuman	Latino	2194	0.317
1014	Nakanai (Loso)	Pacific Islander	16024	0.297

Table S25 27 loci from the combined human data set of 645 loci with no genotype data in at least one population

ID in combined data set	Populations with no genotype data
GATA35_11	Cape Mixed Ancestry, Venda, !Xun/Kxoe, Xhosa, Koma, Beta Israel, Dogon, Temani
GATA11B08P_6	Koma, Beta Israel, Dogon, Temani
TCTA017M_9	Koma, Beta Israel, Dogon, Temani
AGAT139P_20	Koma, Beta Israel, Dogon, Temani
TTTA063P_1	Dogon, Fulani (Nigeria)
TTAT027P_15	Nuer, Shilook
D1S1653	Dogon
GATA51H01_1	Dogon
AGAT117_2	Dogon
D3S3045	Dogon
GATA138B05_5	Australian
GATA142H05P_5	Fulani (Nigeria)
D6S1027	Dogon
GATA61G06_7	Dogon
AGAT049P_7	Nuer
GATA65D11_9	Fulani (Nigeria)
D10S1208	Ewondo
ATA20B07_10	Dogon
ATA44G07M_10	Dogon
D11S4459	Nuer
AGAT110P_13	Shilook
GATA137B09_13	Fulani (Nigeria)
ATA70B03P_14	Nuer
D16S753	Dogon
D17S1294	Dogon
D18S858	Dogon
D19S1034	!Xun\Kxoe

Table S26 10 loci from the combined human-chimpanzee data set of 246 loci with no genotype data in at least one population

ID in combined data set	Populations with no genotype data
TCTA017M_9	Koma, Beta Israel, Dogon, Temani
D1S1653	Dogon
GATA138B05_5	Australian
D6S1027	Dogon
GATA61G06_7	Dogon
D11S4459	Nuer
D12S1042	Bonobo
ATA70B03P_14	Nuer
D17S1294	Dogon
D18S858	Dogon

References

1. Rosenberg N. A., 2006 Standardized subsets of the HGDP-CEPH Human Genome Diversity Cell Line Panel, accounting for atypical and duplicated samples and pairs of close relatives. Ann. Hum. Genet. 70: 841-847.

2. Friedlaender J. S., F. R. Friedlaender , F. A. Reed, K. K. Kidd, J. R. Kidd, G. K. Chambers, R. A. Lea, J. H. Loo, G. Koki, J. A. Hodgson, *et al*., 2008 The genetic structure of Pacific Islanders. PLoS Genet. 4: e19.

3. Tishkoff S. A., F. A. Reed, F. R. Friedlaender, C. Ehret, A. Ranciaro, A. Froment, J. B. Hirbo, A. A. Awomoyi, J. M. Bodo, O. Doumbo, *et al*., 2009 The genetic structure and history of Africans and African Americans. Science 324: 1035-1044.

4. Ramachandran S., O. Deshpande, C. C. Roseman, N. A. Rosenberg, M. W. Feldman, and L. L. Cavalli-Sforza, 2005 Support from the relationship of genetic and geographic distance in human populations for a serial founder effect originating in Africa. Proc. Natl. Acad. Sci. U S A 102: 15942-15947.

5. Rosenberg N. A., S. Mahajan, S. Ramachandran, C. Zhao, J. K. Pritchard, and M. W. Feldman, 2005 Clines, clusters, and the effect of study design on the inference of human population structure. PLoS Genet. 1: e70.

6. Rosenberg N. A., J. K. Pritchard, J. L. Weber, H. M. Cann, K. K. Kidd, L. A. Zhivotovsky, and M. W. Feldman, 2002 Genetic structure of human populations. Science 298: 2381-2385.

7. Wang S., C. M. Lewis, M. Jakobsson, S. Ramachandran, N. Ray, G. Bedoya, W. Rojas, M. V. Parra, J. A. Molina, C. Gallo, *et al*., 2007 Genetic variation and population structure in Native Americans. PLoS Genet. 3: e185.

8. Wang S., N. Ray, W. Rojas, M. V. Parra, G. Bedoya, C. Gallo, G. Poletti, G. Mazzotti, K. Hill, A. M. Hurtado, *et al*., 2008 Geographic patterns of genome admixture in Latin American Mestizos. PLoS Genet. 4: e1000037.

9. Kopelman N. M., L. Stone, C. Wang, D. Gefel, M. W. Feldman, J. Hillel, and N. A. Rosenberg, 2009 Genomic microsatellites identify shared Jewish ancestry intermediate between Middle Eastern and European populations. BMC Genet. 10: 80.

10. Rosenberg N. A., S. Mahajan, C. Gonzalez-Quevedo, M. G. Blum, L. Nino-Rosales, V. Ninis, P. Das, M. Hegde, L. Molinari, G. Zapata, *et al*., 2006 Low levels of genetic divergence across geographically and linguistically diverse populations from India. PLoS Genet. 2: e215.

11. Pemberton T. J., F-Y. Li, E. K. Hanson, N. U. Mehta, S. Choi, J. Ballantyne, J. W. Belmont, N. A. Rosenberg, C. Tyler-Smith, and P. I. Patel, 2012 Impact of restricted marital practices on genetic variation in an endogamous Gujarati group. Am. J. Phys. Anthropol. 149: 92-103.

12. Becquet C., N. Patterson, A. C. Stone, M. Przeworski, and D. Reich, 2007 Genetic structure of chimpanzee populations. PLoS Genet. 3: e66.
