# Supplementary material for: Population Structure in a Comprehensive Genomic Data Set on Human Microsatellite Variation
Source: G3 (Bethesda). 2013 May 1;3(5):891–907. doi: 10.1534/g3.113.005728 (PMC3656735; doi:10.1534/g3.113.005728)
Supplement: Supporting Information [file supp_g3.113.005728_FigureS1.pdf]

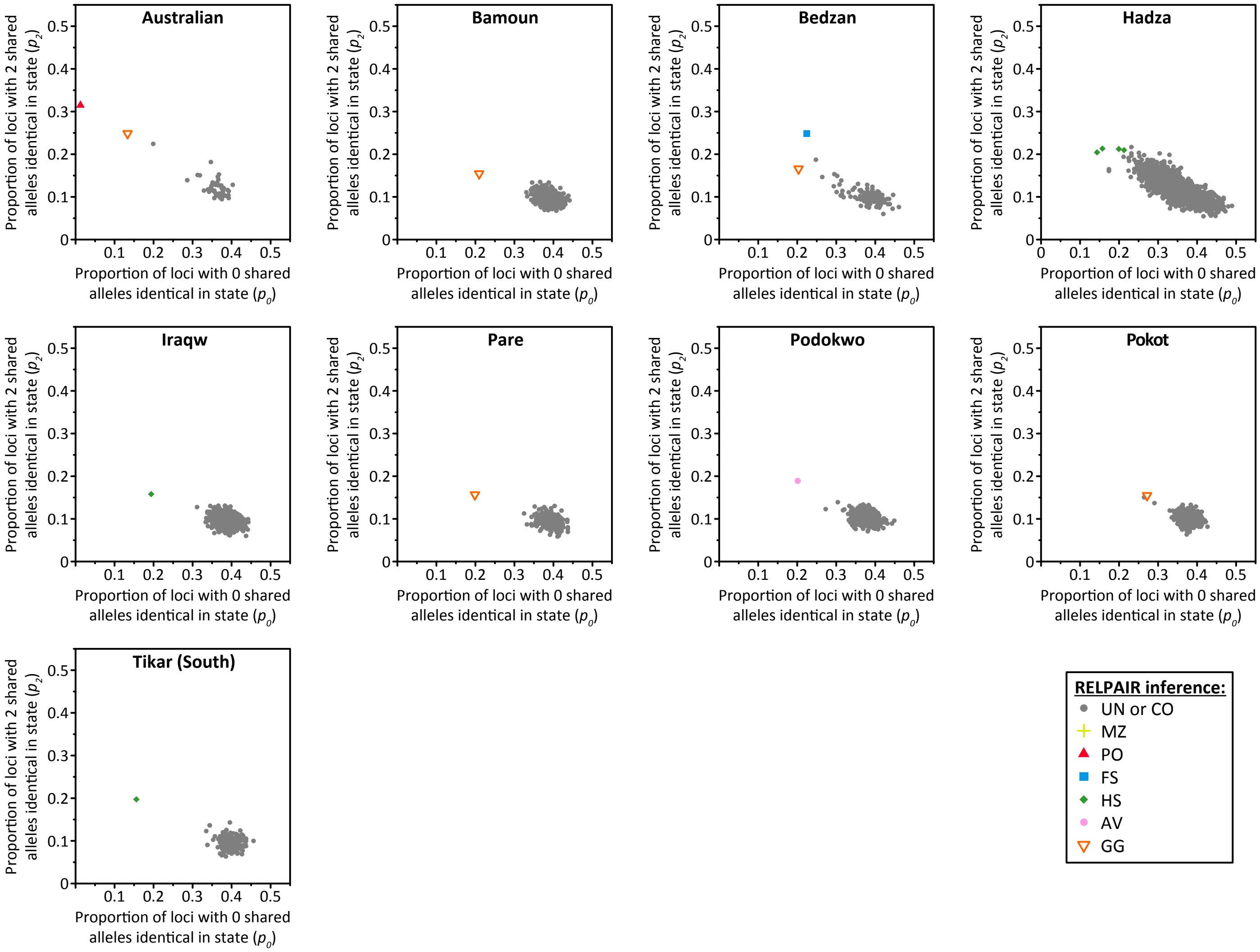

Figure S1 Intra-population allele-sharing for pairs of individuals in those populations in the African data set for which we inferred at least one relative pair. First- and second-degree intra-population relative pairs are reported in Tables S5 and S6, respectively.
