# Supplementary material for: Population Structure in a Comprehensive Genomic Data Set on Human Microsatellite Variation
Source: G3 (Bethesda). 2013 May 1;3(5):891–907. doi: 10.1534/g3.113.005728 (PMC3656735; doi:10.1534/g3.113.005728)
Supplement: Supporting Information [file supp_g3.113.005728_FigureS5.pdf]

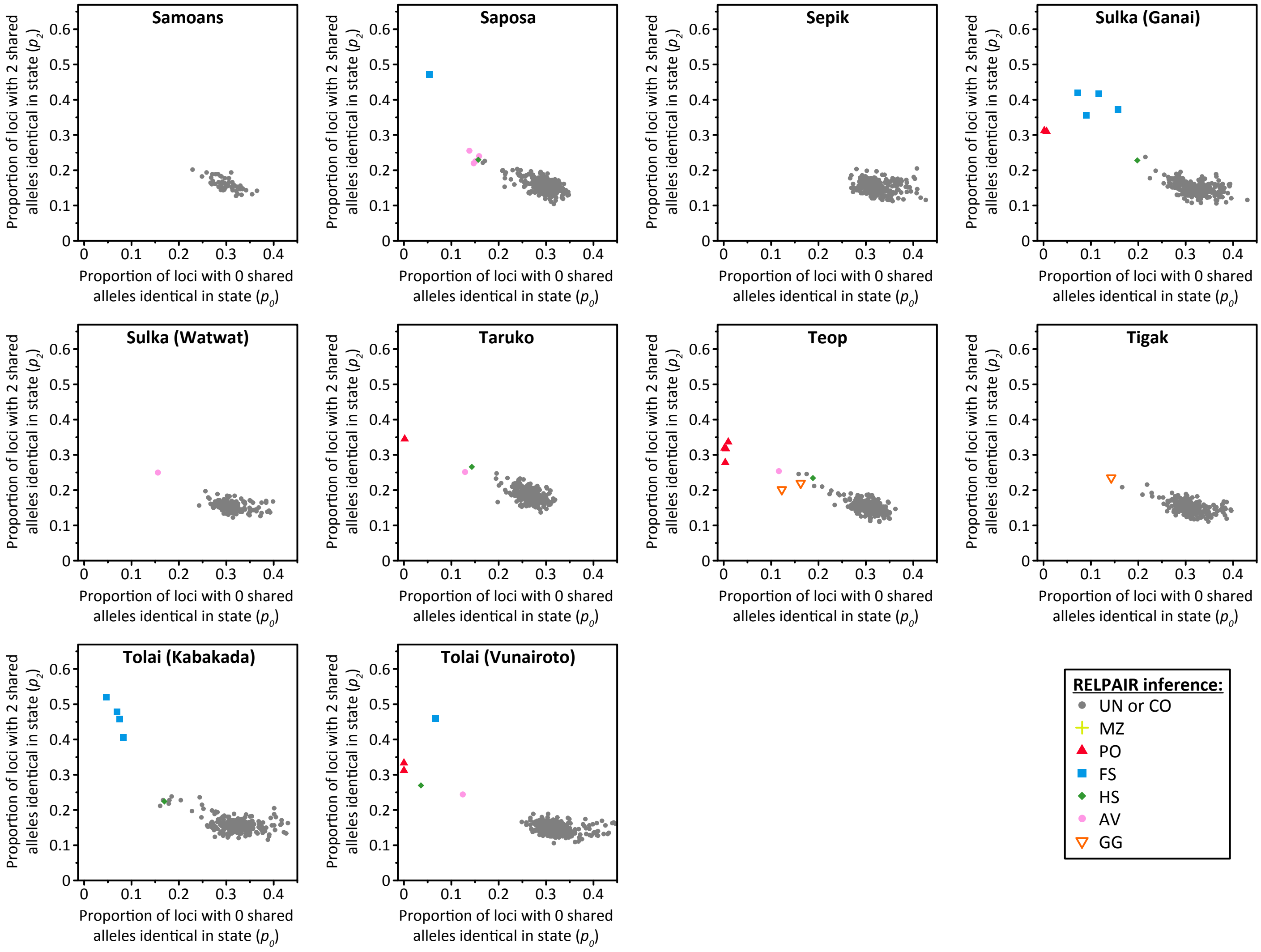

Figure S5 Intra-population allele-sharing for pairs of individuals in the Pacific Islander data set (part 3). First- and second-degree relative pairs are reported in Tables S10 and S11, respectively.
