# Supplementary material for: Population Structure in a Comprehensive Genomic Data Set on Human Microsatellite Variation
Source: G3 (Bethesda). 2013 May 1;3(5):891–907. doi: 10.1534/g3.113.005728 (PMC3656735; doi:10.1534/g3.113.005728)
Supplement: Supporting Information [file supp_g3.113.005728_FigureS6.pdf]

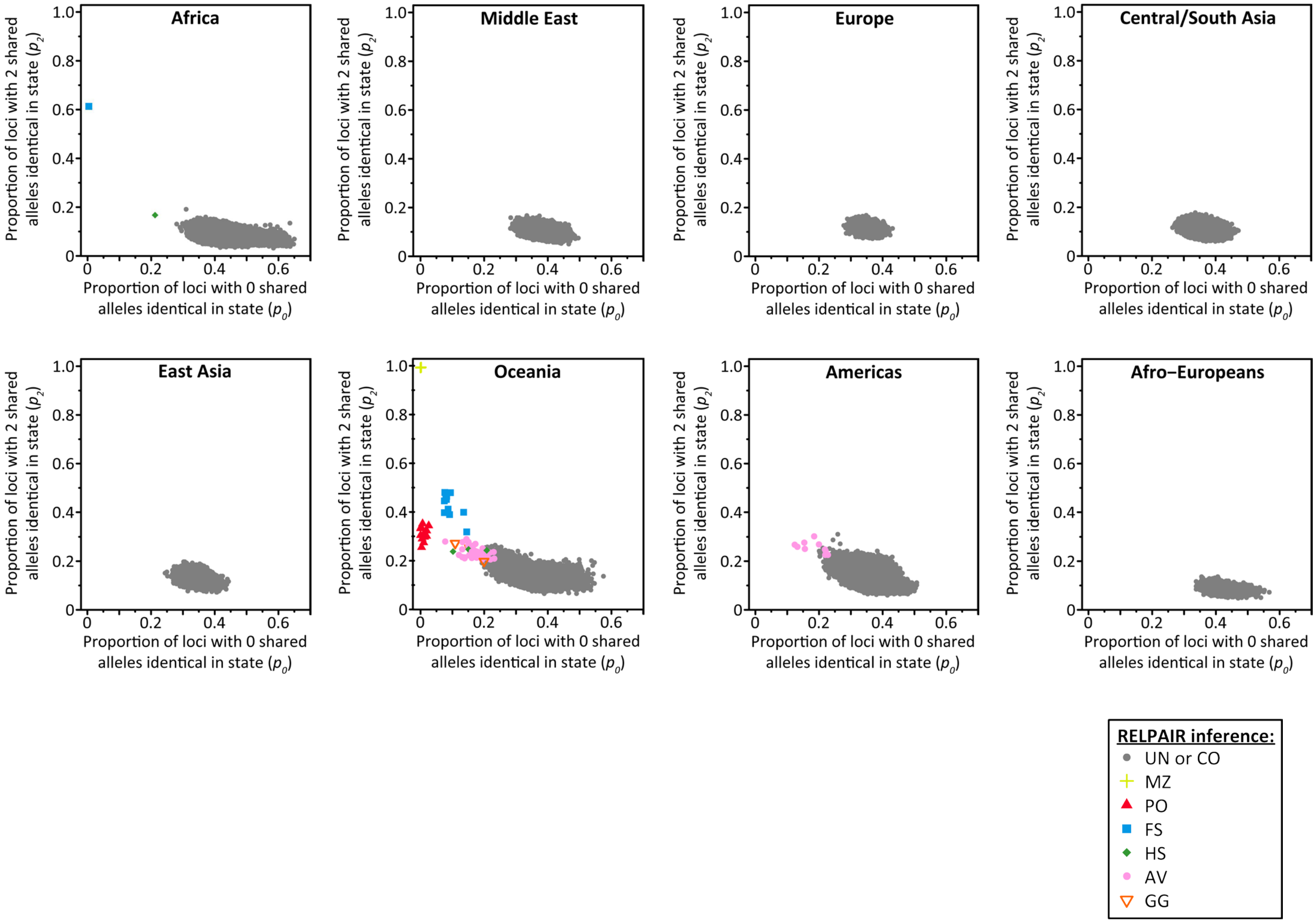

Figure S6 Inter-population allele-sharing for pairs of individuals in each of eight subsets that group populations by their geographic affiliation (Africa, the Middle East, Europe, Central/South Asia, East Asia, Oceania, and the Americas) or admixture status (Afro-European). Latino individuals were included in the Americas analysis, as they were genotyped concurrently with the Native American data set. First- and second-degree relative pairs in the Africa analysis are reported in Tables S13 and S14, respectively. Monozygotic, first-degree, and second-degree relative pairs in the Oceania analysis are reported in Tables S15, S16, and S17, respectively. Second-degree relative pairs in the Americas analysis are reported in Table S19.
