# Supplementary material for: Population Structure in a Comprehensive Genomic Data Set on Human Microsatellite Variation
Source: G3 (Bethesda). 2013 May 1;3(5):891–907. doi: 10.1534/g3.113.005728 (PMC3656735; doi:10.1534/g3.113.005728)
Supplement: Supporting Information [file supp_g3.113.005728_FigureS8.pdf]

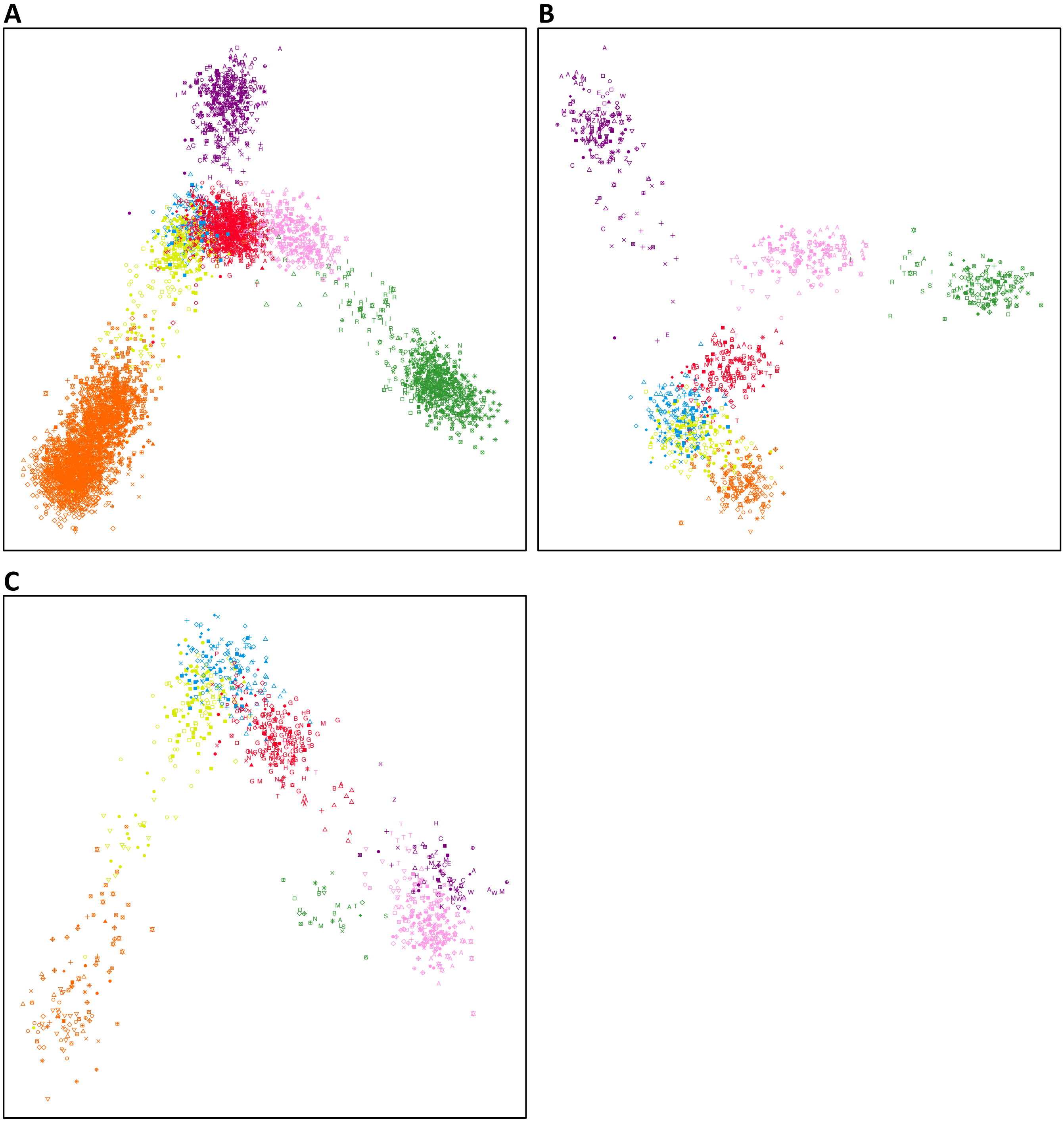

Figure S8 The effect of sample size on population patterns in worldwide multidimensional scaling (MDS) analyses. Procrustes-transformed MDS representations of pairwise allele-sharing distances between (A) 5063 non-admixed individuals in the MS5435 data set, (B) 1106 non-admixed individuals from the MS5435 data set where each geographic region is represented by a random sample of 158 individuals—the smallest sample size across geographic regions—with membership to that region, and (C) 952 non-admixed individuals from the MS5435 data set where each geographic region is represented by the same number of individuals as in subset H952 of the HGDP-CEPH data set [1], chosen at random from all individuals with membership to that region. Individuals are colored by geographic affiliation and indicated by the symbols in Figure 2.
