# Supplementary material for: Population Structure in a Comprehensive Genomic Data Set on Human Microsatellite Variation
Source: G3 (Bethesda). 2013 May 1;3(5):891–907. doi: 10.1534/g3.113.005728 (PMC3656735; doi:10.1534/g3.113.005728)
Supplement: Supporting Information [file supp_g3.113.005728_TableS1.pdf]

Table S1 Allele size adjustments used to make the Pacific Islander data set comparable to the combined HGDP-CEPH, Native American, Latino, Jewish, Asian Indian, and CGP data set

ID in combined data set	ID in Pacific Islander data set	Amount added to genotypes in the Pacific Islander data set (c^*)
AAT107_16	AAT107Z	-53
AAT200_1	AAT200ZP	-137
AATA019_8	AATA019ZP	-131
ATA50C05_6	ATA50C05ZP	116
ATA70B03P_14	ATA70B03ZP	-124
ATA77F05_14	ATA77F05Z	74
D10S1221	ATA21A03Z	73
D10S1412	ATA31G11P	-7
D10S1430	GATA84C01ZP	-39
D11S1304	UT2095M	-1
D11S1392	GATA6B09P	-7
D11S1984	GGAA17G05P	-7
D11S1998	GATA23E06L	-3
D11S1999	GATA23F06L	-3
D11S2000	GATA28D01M	-1
D12S1042	ATA27A06P	-7
D12S1045	ATA29A06P	-7
D12S1052	GATA26D02M	-1
D12S1064	GATA63D12P	-7
D12S1300	GATA85A04M	-1
D12S1301	GATA91H06M	-1
D12S2070	ATA25F09M	-1
D13S1807	GATA11C08P	-7
D13S787	GATA23C03P	-7
D13S796[a]	GATA51B02ZP	-46
D13S895	GGAA22G01ZP	17
D14S608	GATA43H01M	-1
D14S617	GGAA21G11L	-2
D15S1515	GATA197B10P	-7
D15S816	GATA73F01M	-1
D16S2624	GATA81D12M	-1
D16S3253	GATA22F09P	-7
D17S1298	GAAT2C03P	-7
D17S2180	ATC6A06M	-1
D17S2195	ATA58A02P	-7
D18S1376[b]	GATA185C06Z	-17
D19S589	GATA29B01L	1
D19S591	GATA44F10P	-7
D1S1596	GATA26G09P	-7
D1S1612	GGAA3A07M	-1
D1S1627	ATA25E07M	-1
D1S1728	GATA109Z	-130
D1S3669	GATA29A05P	-7
D20S477	GATA29F06Z	-1
D21S1411	UT1355Z	-3

D22S686	GGAA10F06M	-1
D2S1352	ATA27D04P	-7
D2S1363	GATA23D03ZP	-107
D2S1384	GATA52A04M	-1
D2S1391	GATA65C03M	-1
D2S1394	GATA69E12M	-1
D2S1400	GGAA20G10M	-1
D2S2944	GATA30E06P	-7
D2S2968	GATA178G09M	-1
D3S1744[c]	GATA3C02ZP	-26
D3S1768	GATA8B05M	-1
D3S2427	GATA22F11NZ	58
D3S2432	GATA27C08P	-7
D3S4529	GATA128C02M	-1
D4S1627	GATA7D01ZP	37
D4S1652	GATA5B02M	-1
D4S2366	GATA22G05M	-1
D4S2397	ATA27C07P	-7
D4S2417	GATA42H02P	-8
D4S2623	GATA62A12Z	-35
D4S2632	GATA72G09Z	25
D5S1456	GATA11A11P	-7
D5S1462	GATA3H06M	-1
D5S1470	GATA7C06M	-1
D5S1480	ATA23A10M	-1
D5S1725[d]	GATA89G08Z	31
D5S2488	ATA20G07M	-1
D6S1017	GGAT3H10M	-1
D6S1027	ATA22G07P	-7
D6S2436	GATA165G02M	-1
D7S1802	GATA41G07M	-1
D7S1818	GATA24D12P	-7
D7S2204	GATA73D10L	4
D7S2477	035XB9ZP	-70
D7S3056	GATA24F03ZP	7
D7S3070	GATA189C06M	-1
D8S1048	UT7129L	1
D8S1110	GATA8G10M	-1
D8S1132	GATA26E03M	-1
D8S1477[e]	GGAA20C10Z	-104
D8S373	UT721M	-1
D8S592	GATA6B02P	-7
D9S1120	GATA81C04M	-1
D9S2169	GATA62F03M	-1
D9S910	ATA18A07M	-1
GATA138B05_5	GATA138B05ZP	49
GATA169E06_14	GATA169E06ZP	38
GATA66D01_2	GATA66D01ZP	-110
GGAA30H04_14	GGAA30H04ZP	-103
NA.D18S.1	GATA178F11Z	67

NA.D1S.3	GATA133A08Q	-4
TPO.D2S	SRAP	-7

[a]Friedlaender *et al*. [2] used an adjustment of -45 nt.

[b]This locus was not present in the list of adjusted loci reported by Friedlaender *et al*. [2].

[c]Friedlaender *et al*. [2] used an adjustment of -25 nt.

[d]Friedlaender *et al*. [2] used an adjustment of 27 nt.

[e]Friedlaender *et al*. [2] used an adjustment of -103 nt.
