# Supplementary material for: Population Structure in a Comprehensive Genomic Data Set on Human Microsatellite Variation
Source: G3 (Bethesda). 2013 May 1;3(5):891–907. doi: 10.1534/g3.113.005728 (PMC3656735; doi:10.1534/g3.113.005728)
Supplement: Supporting Information [file supp_g3.113.005728_TableS3.pdf]

Table S3 11 loci excluded from the combined data set of 656 loci due to >10% missing data

Locus ID	Fraction of individuals with missing genotypes
D2S1780	0.287
D9S938	0.192
GTTTT002	0.188
AGAT135	0.177
AAT258	0.175
NA.D1S.1	0.171
D1S2134	0.167
AAT267	0.161
AGAT017	0.157
D1S3721	0.147
D3S2432	0.147
