# Supplementary material for: Population Structure in a Comprehensive Genomic Data Set on Human Microsatellite Variation
Source: G3 (Bethesda). 2013 May 1;3(5):891–907. doi: 10.1534/g3.113.005728 (PMC3656735; doi:10.1534/g3.113.005728)
Supplement: Supporting Information [file supp_g3.113.005728_TableS4.pdf]

Table S4 68 individuals excluded from the combined data set of 5916 individuals due to >27.5% missing data

Population			Identification number of individual	Fraction of loci with missing genotypes
ID	Name	Data set of origin		
1009	Mengen	Pacific Islander	14131	0.868
1009	Mengen	Pacific Islander	14141	0.786
1116	Ngumba	African	70753	0.586
1280	Temani	African	103376	0.524
1278	Dogon	African	103335	0.513
1280	Temani	African	103372	0.510
811	Chipewyan	Native American	2384	0.501
1013	Nakanai (Bileki)	Pacific Islander	15241	0.491
845	Ticuna (Arara)	Native American	2547	0.490
1022	Tolai (Kabakada)	Pacific Islander	22071	0.478
824	Zapotec	Native American	2091	0.471
1017	Ata (Uasilau)	Pacific Islander	7003	0.471
1272	Cape Mixed Ancestry	African	103215	0.468
1005	Anem (Keraiai)	Pacific Islander	4031	0.457
1268	Nuer	African	73132	0.457
884	Catamarca	Latino	2154	0.456
1273	Venda	African	103242	0.456
1254	Wimbum	African	72932	0.451
871	Oriente	Latino	2219	0.440
822	Mixtec	Native American	2042	0.434
1034	Teop	Pacific Islander	35111	0.434
843	Inga	Native American	2509	0.431
831	Guaymi	Native American	2009	0.420
690	Tundra Nentsi	Native American	2457	0.419
1271	Sara (Various)	African	73026	0.419
1278	Dogon	African	103333	0.419
1241	Maasai (Ilchamus)	African	72708	0.411
1272	Cape Mixed Ancestry	African	103216	0.411
837	Kaingang	Native American	2757	0.408
824	Zapotec	Native American	2082	0.406
831	Guaymi	Native American	2003	0.398
884	Catamarca	Latino	2150	0.398
836	Ache	Native American	2742	0.389
1235	Gabra	African	72527	0.388
1278	Dogon	African	103339	0.386
1278	Dogon	African	103352	0.383
1276	Koma	African	103304	0.369
1278	Dogon	African	103332	0.367
1012	Kol	Pacific Islander	8281	0.364
1274	!Xun/Kxoe	African	103258	0.364
849	Arhuaco	Native American	2733	0.363
1280	Temani	African	103385	0.353
1272	Cape Mixed Ancestry	African	103186	0.349
836	Ache	Native American	2737	0.347
1273	Venda	African	103248	0.346
1101	Hadza	African	71463	0.343
54	Hazara	HGDP-CEPH	127	0.341

1022	Tolai (Kabakada)	Pacific Islander	22041	0.341
812	Cree	Native American	2403	0.340
1272	Cape Mixed Ancestry	African	103211	0.340
1274	!Xun/Kxoe	African	103260	0.338
514	Punjabi	Asian Indian	63000346	0.333
57	Makrani	HGDP-CEPH	139	0.332
837	Kaingang	Native American	2756	0.332
1272	Cape Mixed Ancestry	African	103206	0.330
1275	Xhosa	African	103287	0.326
1101	Hadza	African	71454	0.322
845	Ticuna (Arara)	Native American	2541	0.316
834	Huilliche	Native American	2122	0.313
1276	Koma	African	103306	0.305
901	Ashkenazi Jewish	Jewish	2315	0.301
1011	Sulka (Watwat)	Pacific Islander	21163	0.299
841	Kogi	Native American	2472	0.298
1107	Burunge	African	70347	0.290
1259	Baluba	African	73014	0.288
1272	Cape Mixed Ancestry	African	103210	0.287
1278	Dogon	African	103343	0.279
1229	Kikuyu	African	72381	0.278
