# Supplementary material for: Population Structure in a Comprehensive Genomic Data Set on Human Microsatellite Variation
Source: G3 (Bethesda). 2013 May 1;3(5):891–907. doi: 10.1534/g3.113.005728 (PMC3656735; doi:10.1534/g3.113.005728)
Supplement: Supporting Information [file supp_g3.113.005728_TableS5.pdf]

Table S5 Two previously unreported intra-population first-degree relative pairs in the African data set

Population		Identification number		RELPAIR inference: parent/offspring (PO) or full-sibling (FS)	Support for inference: RELPAIR (R) or allele-sharing (A)
ID	Name	First individual	Second individual		
1222	Australian	79193	79195	PO	R,A
1114	Bedzan	71580	71584	FS	R[†]

[†]Allele-sharing suggests this pair is a second-degree relative pair (Figure S1). However, in the RELPAIR analysis, the likelihood ratio statistic for all other relative types was <0.0001 for this pair. To be conservative, this pair was treated as a first-degree relative pair when creating the standardized subsets MS5547 and MS5435.
