# Supplementary material for: Population Structure in a Comprehensive Genomic Data Set on Human Microsatellite Variation
Source: G3 (Bethesda). 2013 May 1;3(5):891–907. doi: 10.1534/g3.113.005728 (PMC3656735; doi:10.1534/g3.113.005728)
Supplement: Supporting Information [file supp_g3.113.005728_TableS6.pdf]

Table S6 12 previously unreported intra-population second-degree relative pairs in the African data set

Population		Identification number		RELPAIR inference: Avuncular (AV), grandparental (GG), or half-sibling (HS)	Support for inference: RELPAIR (R) or allele-sharing (A)
ID	Name	First individual	Second individual		
1132	Podokwo	73033	72886	AV	R,A
1110	Pare	70446	70452	GG	R,A
1114	Bedzan	71578	70672	GG	R,A
1127	Bamoun	71053	71054	GG	R,A
1222	Australian	79194	79195	GG	R,A
1239	Pokot	72632	72636	GG	R,A
1101	Hadza	70030	70027	HS	R,A
1101	Hadza	70047	70038	HS	R,A
1101	Hadza	70048	70071	HS	R,A
1101	Hadza	71474	70079	HS	R,A
1103	Iraqw	70207	70204	HS	R,A
1200	Tikar (South)	71729	71731	HS	R,A
