# Supplementary material for: Population Structure in a Comprehensive Genomic Data Set on Human Microsatellite Variation
Source: G3 (Bethesda). 2013 May 1;3(5):891–907. doi: 10.1534/g3.113.005728 (PMC3656735; doi:10.1534/g3.113.005728)
Supplement: Supporting Information [file supp_g3.113.005728_TableS7.pdf]

Table S7 Two previously unreported intra-population first-degree relative pairs in the Latino data set

Population		Identification number		RELPAIR inference: parent/offspring (PO) or full-sibling (FS)	Support for inference: RELPAIR (R) or allele-sharing (A)
ID	Name	First individual	Second individual		
883	Paposo	2264	2266	PO	R,A
892	Peque	2623	2639	FS	R,A
