# Supplementary material for: Population Structure in a Comprehensive Genomic Data Set on Human Microsatellite Variation
Source: G3 (Bethesda). 2013 May 1;3(5):891–907. doi: 10.1534/g3.113.005728 (PMC3656735; doi:10.1534/g3.113.005728)
Supplement: Supporting Information [file supp_g3.113.005728_TableS8.pdf]

Table S8 Four previously unreported intra-population second-degree relative pairs in the Latino data set

Population		Identification number		RELPAIR inference: Avuncular (AV), grandparental (GG), or half-sibling (HS)	Support for inference: RELPAIR (R) or allele-sharing (A)
**ID**	**Name**	**First individual**	**Second individual**		
882	Quetalmahue	2282	2288	AV	R,A
885	Salta	2169	2180	AV	R,A
883	Paposo	2267	2268	HS	R,A
883	Paposo	2267	2274	HS	R,A
