# Supplementary material for: Population Structure in a Comprehensive Genomic Data Set on Human Microsatellite Variation
Source: G3 (Bethesda). 2013 May 1;3(5):891–907. doi: 10.1534/g3.113.005728 (PMC3656735; doi:10.1534/g3.113.005728)
Supplement: Supporting Information [file supp_g3.113.005728_TableS9.pdf]

Table S9 Six previously unreported intra-population monozygotic pairs in the Pacific Islander data set

Population		Identification number		Support for inference: RELPAIR (R) or allele-sharing (A)
ID	Name	First individual	Second individual	
1040	Micronesians	53071	53141	R,A
1040	Micronesians	53081	53151	R,A
1040	Micronesians	53091	53161	R,A
1040	Micronesians	53101	53181	R,A
1040	Micronesians	53111	53191	R,A
1040	Micronesians	53131	53061	R,A
