# Supplementary material for: Population Structure in a Comprehensive Genomic Data Set on Human Microsatellite Variation
Source: G3 (Bethesda). 2013 May 1;3(5):891–907. doi: 10.1534/g3.113.005728 (PMC3656735; doi:10.1534/g3.113.005728)
Supplement: Supporting Information [file supp_g3.113.005728_TableS10.pdf]

Table S10 127 previously unreported intra-population first-degree relative pairs in the Pacific Islander data set

Population		Identification number		RELPAIR inference: parent/offspring (PO) or full-sibling (FS)	Support for inference: RELPAIR (R) or allele-sharing (A)
ID	Name	First individual	Second individual		
1001	East Highlands (Gimi & Goroka)	54071	54061	PO	R,A
1001	East Highlands (Gimi & Goroka)	54091	54071	PO	R,A
1005	Anem (Keraiai)	4051	4091	PO	R,A
1005	Anem (Keraiai)	4061	4051	PO	R,A
1005	Anem (Keraiai)	4141	4021	PO	R,A
1006	Anem (Purailing)	5041	5061	PO	R,A
1006	Anem (Purailing)	5053	5061	PO	R,A
1007	Mangseng	12021	12111	PO	R,A
1007	Mangseng	12041	12091	PO	R,A
1008	Melamela	13071	13111	PO	R,A
1010	Sulka (Ganai)	20171	20193	PO	R,A
1010	Sulka (Ganai)	20171	20201	PO	R,A
1012	Kol	8021	8071	PO	R,A
1012	Kol	8113	8051	PO	R,A
1014	Nakanai (Loso)	16001	16041	PO	R,A
1014	Nakanai (Loso)	16131	16061	PO	R,A
1016	Mamusi (Lingite)	11121	11101	PO	R,A
1018	Ata (Lugei)	6013	6123	PO	R,A
1018	Ata (Lugei)	6161	6231	PO	R,A
1020	Baining (Marabu)	18191	18111	PO	R,A
1020	Baining (Marabu)	18221	18153	PO	R,A
1020	Baining (Marabu)	18241	18021	PO	R,A
1020	Baining (Marabu)	18241	18041	PO	R,A
1023	Tolai (Vunairoto)	23212	23214	PO	R,A
1023	Tolai (Vunairoto)	23213	23212	PO	R,A
1024	Mussau	27041	27201	PO	R,A
1024	Mussau	27201	27101	PO	R,A
1024	Mussau	27221	27181	PO	R,A
1025	Lavongai (North)	25033	25001	PO	R,A
1025	Lavongai (North)	25041	25181	PO	R,A
1025	Lavongai (North)	25051	25121	PO	R,A
1026	Lavongai (South)	26141	26154	PO	R,A
1026	Lavongai (South)	26141	26171	PO	R,A
1026	Lavongai (South)	26231	26201	PO	R,A
1028	Nalik	31071	31101	PO	R,A
1028	Nalik	31071	31111	PO	R,A
1028	Nalik	31161	31173	PO	R,A
1028	Nalik	31161	31193	PO	R,A
1029	Notsi	32131	32171	PO	R,A
1029	Notsi	32143	32181	PO	R,A
1030	Kuot (Kabil)	28101	28211	PO	R,A
1032	Madak	30021	30081	PO	R,A
1032	Madak	30053	30221	PO	R,A
1032	Madak	30211	30161	PO	R,A
1034	Teop	35061	35221	PO	R,A

1034	Teop	35071	35001	PO	R,A
1034	Teop	35181	35211	PO	R,A
1034	Teop	35201	35221	PO	R,A
1035	Aita	36061	36121	PO	R,A
1035	Aita	36073	36003	PO	R,A
1035	Aita	36073	36011	PO	R,A
1037	Nasioi	663	52004	PO	R,A
1037	Nasioi	52004	662	PO	R,A
1037	Nasioi	52041	658	PO	R,A
1042	Maoris	62161	62181	PO	R,A
1044	Taruko	42161	42141	PO	R,A
1004	Kove	9201	9241	FS	R,A
1005	Anem (Keraiai)	4101	4013	FS	R,A
1006	Anem (Purailing)	5053	5041	FS	R,A
1006	Anem (Purailing)	5071	5151	FS	R,A
1006	Anem (Purailing)	5181	5101	FS	R,A
1007	Mangseng	12021	12034	FS	R,A
1007	Mangseng	12033	12021	FS	R,A
1007	Mangseng	12033	12034	FS	R,A
1007	Mangseng	12131	12101	FS	R,A
1009	Mengen	14001	14021	FS	R,A
1009	Mengen	14081	14071	FS	R,A
1009	Mengen	14111	14071	FS	R,A
1009	Mengen	14111	14081	FS	R,A
1010	Sulka (Ganai)	20013	20161	FS	R,A
1010	Sulka (Ganai)	20031	20001	FS	R,A
1010	Sulka (Ganai)	20201	20193	FS	R,A
1010	Sulka (Ganai)	20241	20221	FS	R,A
1014	Nakanai (Loso)	16051	16001	FS	R,A
1015	Mamusi (Kisiluvi)	10091	10161	FS	R,A
1015	Mamusi (Kisiluvi)	10241	10111	FS	R,A
1016	Mamusi (Lingite)	11011	11141	FS	R,A
1016	Mamusi (Lingite)	11031	11131	FS	R,A
1016	Mamusi (Lingite)	11051	11011	FS	R,A
1016	Mamusi (Lingite)	11051	11141	FS	R,A
1016	Mamusi (Lingite)	11071	11031	FS	R,A
1016	Mamusi (Lingite)	11071	11131	FS	R,A
1016	Mamusi (Lingite)	11091	11191	FS	R,A
1018	Ata (Lugei)	6003	6013	FS	R,A
1018	Ata (Lugei)	6101	6071	FS	R,A
1018	Ata (Lugei)	6181	6051	FS	R,A
1019	Baining (Malasait)	17011	17211	FS	R,A
1019	Baining (Malasait)	17051	17141	FS	R,A
1019	Baining (Malasait)	17091	17081	FS	R,A
1020	Baining (Marabu)	18041	18021	FS	R,A
1020	Baining (Marabu)	18171	18131	FS	R,A
1020	Baining (Marabu)	18171	18201	FS	R,A
1020	Baining (Marabu)	18201	18131	FS	R,A
1020	Baining (Marabu)	18211	18001	FS	R,A
1021	Baining (Rangulit)	19011	19101	FS	R,A

1021	Baining (Rangulit)	19071	19201	FS	R,A
1021	Baining (Rangulit)	19081	19151	FS	R,A
1022	Tolai (Kabakada)	22113	22091	FS	R,A
1022	Tolai (Kabakada)	22113	22121	FS	R,A
1022	Tolai (Kabakada)	22121	22091	FS	R,A
1022	Tolai (Kabakada)	22191	22211	FS	R,A
1023	Tolai (Vunairoto)	23213	23214	FS	R,A
1024	Mussau	27081	27031	FS	R,A
1026	Lavongai (South)	26171	26154	FS	R,A
1028	Nalik	31101	31111	FS	R,A
1028	Nalik	31134	31031	FS	R,A
1028	Nalik	31144	31121	FS	R,A
1029	Notsi	32061	32081	FS	R,A
1029	Notsi	32061	32091	FS	R,A
1029	Notsi	32091	32081	FS	R,A
1030	Kuot (Kabil)	28031	28021	FS	R,A
1030	Kuot (Kabil)	28091	28073	FS	R,A
1031	Kuot (Lamalaua)	29171	29001	FS	R,A
1032	Madak	30061	30171	FS	R,A
1032	Madak	30061	30221	FS	R,A
1032	Madak	30221	30171	FS	R,A
1033	Saposa	34021	34121	FS	R,A
1035	Aita	36021	36151	FS	R,A
1035	Aita	36031	36081	FS	R,A
1035	Aita	36221	36231	FS	R,A
1037	Nasioi	52004	490	FS	R,A
1037	Nasioi	52041	664	FS	R,A
1042	Maoris	62011	62171	FS	R,A
1042	Maoris	62221	62011	FS	R,A
1042	Maoris	62221	62171	FS	R,A
1043	Ami	47181	47221	FS	R[†]
1043	Ami	47211	47191	FS	R,A

[†]Allele-sharing suggests this pair is a second-degree relative pair (Figure S3). In the RELPAIR analysis, the likelihood ratio statistic for HS was the next highest after FS for this pair. To be conservative, this pair was treated as a first-degree relative pair when creating the standardized subsets MS5547 and MS5435.
