# Supplementary material for: Population Structure in a Comprehensive Genomic Data Set on Human Microsatellite Variation
Source: G3 (Bethesda). 2013 May 1;3(5):891–907. doi: 10.1534/g3.113.005728 (PMC3656735; doi:10.1534/g3.113.005728)
Supplement: Supporting Information [file supp_g3.113.005728_TableS11.pdf]

Table S11 87 previously unreported intra-population second-degree relative pairs in the Pacific Islander data set

Population		Identification number		RELPAIR inference: Avuncular (AV), grandparental (GG), or half-sibling (HS)	Support for inference: RELPAIR (R) or allele-sharing (A)
ID	Name	First individual	Second individual		
1001	East Highlands (Gimi & Goroka)	54001	54011	AV	R,A
1004	Kove	9111	9221	AV	R,A
1004	Kove	9151	9031	AV	R,A
1007	Mangseng	12053	12041	AV	R,A
1007	Mangseng	12121	12071	AV	R,A
1008	Melamela	13001	13181	AV	R,A
1011	Sulka (Watwat)	21081	21031	AV	R,A
1012	Kol	8171	8161	AV	R,A
1013	Nakanai (Bileki)	15111	15151	AV	R,A
1015	Mamusi (Kisiluvi)	10061	10241	AV	R,A
1015	Mamusi (Kisiluvi)	10211	10121	AV	R,A
1015	Mamusi (Kisiluvi)	10241	10121	AV	R,A
1017	Ata (Uasilau)	7131	7181	AV	R,A
1018	Ata (Lugei)	6003	6123	AV	R,A
1018	Ata (Lugei)	6021	6201	AV	R,A
1019	Baining (Malasait)	17161	17201	AV	R,A
1020	Baining (Marabu)	18031	18131	AV	R,A
1021	Baining (Rangulit)	19001	19021	AV	R,A
1021	Baining (Rangulit)	19111	19081	AV	R,A
1023	Tolai (Vunairoto)	23071	23131	AV	R,A
1025	Lavongai (North)	25041	25001	AV	R,A
1025	Lavongai (North)	25121	25091	AV	R,A
1026	Lavongai (South)	26011	26091	AV	R,A
1028	Nalik	31211	31121	AV	R,A
1030	Kuot (Kabil)	28073	28171	AV	R,A
1030	Kuot (Kabil)	28081	28181	AV	R,A
1030	Kuot (Kabil)	28091	28171	AV	R,A
1031	Kuot (Lamalaua)	29041	29131	AV	R,A
1032	Madak	30191	30104	AV	R,A
1033	Saposa	34011	34041	AV	R,A
1033	Saposa	34033	34151	AV	R,A
1033	Saposa	34111	34231	AV	R,A
1033	Saposa	34171	34191	AV	R,A
1034	Teop	35201	35211	AV	R,A
1035	Aita	36051	36211	AV	R,A
1037	Nasioi	825	823	AV	R,A
1044	Taruko	42151	42201	AV	R,A
1004	Kove	9091	9121	GG	R,A
1005	Anem (Keraiai)	4081	4051	GG	R,A
1005	Anem (Keraiai)	4141	4131	GG	R,A
1006	Anem (Purailing)	5181	5083	GG	R,A
1008	Melamela	13051	13141	GG	R,A
1008	Melamela	13091	13193	GG	R,A
1008	Melamela	13131	13211	GG	R,A
1008	Melamela	13231	13141	GG	R,A

1013	Nakanai (Bileki)	15121	15235	GG	R,A
1013	Nakanai (Bileki)	15161	15121	GG	R,A
1014	Nakanai (Loso)	16051	16023	GG	R,A
1018	Ata (Lugei)	6181	6041	GG	R,A
1020	Baining (Marabu)	18051	18191	GG	R,A
1020	Baining (Marabu)	18171	18031	GG	R,A
1020	Baining (Marabu)	18221	18241	GG	R,A
1021	Baining (Rangulit)	19111	19101	GG	R,A
1027	Tigak	33081	33051	GG	R,A
1028	Nalik	31051	31144	GG	R,A
1031	Kuot (Lamalaua)	29091	29131	GG	R,A
1032	Madak	30081	30141	GG	R,A
1032	Madak	30184	30111	GG	R,A
1034	Teop	35043	35051	GG	R,A
1034	Teop	35201	35061	GG	R,A
1035	Aita	36151	36003	GG	R,A
1035	Aita	36191	36091	GG	R,A
1004	Kove	9014	9133	HS	R,A
1004	Kove	9091	9001	HS	R,A
1004	Kove	9201	9051	HS	R,A
1005	Anem (Keraiai)	4061	4091	HS	R,A
1005	Anem (Keraiai)	4131	4021	HS	R,A
1005	Anem (Keraiai)	4181	4071	HS	R,A
1010	Sulka (Ganai)	20161	20072	HS	R,A
1012	Kol	8001	8081	HS	R,A
1013	Nakanai (Bileki)	15063	15221	HS	R,A
1013	Nakanai (Bileki)	15211	15204	HS	R,A
1015	Mamusi (Kisiluvi)	10121	10111	HS	R,A
1018	Ata (Lugei)	6081	6031	HS	R,A
1022	Tolai (Kabakada)	22231	22051	HS	R,A
1023	Tolai (Vunairoto)	23142	23143	HS	R,A
1026	Lavongai (South)	26121	26091	HS	R,A
1026	Lavongai (South)	26191	26211	HS	R,A
1028	Nalik	31211	31144	HS	R,A
1030	Kuot (Kabil)	28141	28081	HS	R,A
1032	Madak	30081	30171	HS	R,A
1032	Madak	30231	30072	HS	R,A
1033	Saposa	34071	34151	HS	R,A
1034	Teop	35024	35001	HS	R,A
1035	Aita	36003	36161	HS	R,A
1035	Aita	36021	36003	HS	R,A
1044	Taruko	42141	42111	HS	R,A
