# Supplementary material for: Population Structure in a Comprehensive Genomic Data Set on Human Microsatellite Variation
Source: G3 (Bethesda). 2013 May 1;3(5):891–907. doi: 10.1534/g3.113.005728 (PMC3656735; doi:10.1534/g3.113.005728)
Supplement: Supporting Information [file supp_g3.113.005728_TableS12.pdf]

Table S12 13 previously unreported intra-population parent/parent/offspring trios in the Pacific Islander data set

Population		Identification number			Support for inference: RELPAIR (R) or allele-sharing (A)
ID	Name	Parent 1	Parent 2	Offspring	
1001	East Highlands (Gimi & Goroka)	54061	54091	54071	R,A
1005	Anem (Keraiai)	4061	4091	4051	R,A
1006	Anem (Purailing)	5041	5053	5061	R,A
1010	Sulka (Ganai)	20193	20201	20171	R,A
1020	Baining (Marabu)	18021	18041	18241	R,A
1023	Tolai (Vunairoto)	23213	23214	23212	R,A
1024	Mussau	27041	27101	27201	R,A
1026	Lavongai (South)	26154	26171	26141	R,A
1028	Nalik	31101	31111	31071	R,A
1028	Nalik	31173	31193	31161	R,A
1034	Teop	35061	35201	35221	R,A
1035	Aita	36003	36011	36073	R,A
1037	Nasioi	662	663	52004	R,A
