# Supplementary material for: Population Structure in a Comprehensive Genomic Data Set on Human Microsatellite Variation
Source: G3 (Bethesda). 2013 May 1;3(5):891–907. doi: 10.1534/g3.113.005728 (PMC3656735; doi:10.1534/g3.113.005728)
Supplement: Supporting Information [file supp_g3.113.005728_TableS14.pdf]

Table S14 One previously unreported inter-population second-degree relative pair in the African data set

First Individual			Second Individual			RELPAIR inference: Avuncular (AV), grandparental (GG), or half-sibling (HS)	Support for inference: RELPAIR (R) or allele-sharing (A)
Population		Identification number	Population		Identification number		
ID	Name		ID	Name			
1113	Bakola	70618	1115	Mvae	70717	HS	R,A
