# Supplementary material for: Population Structure in a Comprehensive Genomic Data Set on Human Microsatellite Variation
Source: G3 (Bethesda). 2013 May 1;3(5):891–907. doi: 10.1534/g3.113.005728 (PMC3656735; doi:10.1534/g3.113.005728)
Supplement: Supporting Information [file supp_g3.113.005728_TableS15.pdf]

Table S15 Two previously unreported inter-population monozygotic pairs in the Pacific Islander data set

First individual			Second individual			Support for inference: RELPAIR (R) or allele-sharing (A)
Population		Identification number	Population		Identification number	
ID	Name		ID	Name		
1018	Ata (Lugei)	6021	1026	Lavongai (North)	26051	R,A
1040	Micronesians	53171	1041	Samoans	55001	R,A
