# Supplementary material for: Population Structure in a Comprehensive Genomic Data Set on Human Microsatellite Variation
Source: G3 (Bethesda). 2013 May 1;3(5):891–907. doi: 10.1534/g3.113.005728 (PMC3656735; doi:10.1534/g3.113.005728)
Supplement: Supporting Information [file supp_g3.113.005728_TableS16.pdf]

Table S16 24 previously unreported inter-population first-degree relative pairs in the Pacific Islander data set

First individual			Second individual			RELPAIR inference: parent/offspring (PO) or full-sibling (FS)	Support for inference: RELPAIR (R) or allele-sharing (A)
Population		Identification number	Population		Identification number		
ID	Name		ID	Name			
1003	Sepik	3091	1026	Lavongai (North)	26163	PO	R,A
1003	Sepik	3191	1029	Madak	32161	PO	R,A
1003	Sepik	3171	1032	Notsi	30201	PO	R,A
1005	Anem (Keraiai)	4021	1006	Anem (Purailing)	5021	PO	R,A
1005	Anem (Keraiai)	4081	1006	Anem (Purailing)	5011	PO	R,A
1005	Anem (Keraiai)	4181	1006	Anem (Purailing)	5111	PO	R,A
1005	Anem (Keraiai)	4201	1006	Anem (Purailing)	5101	PO	R,A
1005	Anem (Keraiai)	4201	1006	Anem (Purailing)	5181	PO	R,A
1010	Sulka (Ganai)	20101	1011	Sulka (Watwat)	21081	PO	R,A
1015	Mamusi (Kisiluvi)	10103	1016	Mamusi (Lingite)	11081	PO	R,A
1022	Tolai (Kabakada)	22131	1023	Tolai (Vunairoto)	23212	PO	R,A
1028	Nalik	31221	1029	Notsi	32021	PO	R,A
1005	Anem (Keraiai)	4114	1006	Anem (Purailing)	5091	FS	R,A
1010	Sulka (Ganai)	20091	1011	Sulka (Watwat)	21101	FS	R,A
1014	Nakanai (Loso)	16061	1018	Ata (Lugei)	6211	FS	R,A
1015	Mamusi (Kisiluvi)	10051	1016	Mamusi (Lingite)	11031	FS	R,A
1015	Mamusi (Kisiluvi)	10051	1016	Mamusi (Lingite)	11071	FS	R,A
1015	Mamusi (Kisiluvi)	10051	1016	Mamusi (Lingite)	11131	FS	R,A
1015	Mamusi (Kisiluvi)	10181	1016	Mamusi (Lingite)	11221	FS	R[†]
1022	Tolai (Kabakada)	22091	1023	Tolai (Vunairoto)	23152	FS	R,A
1022	Tolai (Kabakada)	22113	1023	Tolai (Vunairoto)	23152	FS	R,A
1022	Tolai (Kabakada)	22121	1023	Tolai (Vunairoto)	23152	FS	R,A
1022	Tolai (Kabakada)	22131	1023	Tolai (Vunairoto)	23213	FS	R,A
1022	Tolai (Kabakada)	22131	1023	Tolai (Vunairoto)	23214	FS	R,A

[†]Allele-sharing suggests this pair is a second-degree relative pair (Figure S6). In the RELPAIR analysis, the likelihood ratio statistic for AV was the next highest after FS for this pair. To be conservative, this pair was treated as a first-degree relative pair when creating the MS5795 and MS5879 data sets.
