# Supplementary material for: Population Structure in a Comprehensive Genomic Data Set on Human Microsatellite Variation
Source: G3 (Bethesda). 2013 May 1;3(5):891–907. doi: 10.1534/g3.113.005728 (PMC3656735; doi:10.1534/g3.113.005728)
Supplement: Supporting Information [file supp_g3.113.005728_TableS17.pdf]

Table S17 54 previously unreported inter-population second-degree relative pairs in the Pacific Islander data set

First individual			Second individual			RELPAIR inference: Avuncular (AV), grandparental (GG), or half-sibling (HS)	Support for inference: RELPAIR (R) or allele-sharing (A)
Population		Identification number	Population		Identification number		
ID	Name			Name			
1005	Anem (Keraiai)	4001	1006	Anem (Purailing)	5031	AV	R,A
1005	Anem (Keraiai)	4051	1006	Anem (Purailing)	5091	AV	R,A
1005	Anem (Keraiai)	4071	1006	Anem (Purailing)	5191	AV	R,A
1005	Anem (Keraiai)	4131	1006	Anem (Purailing)	5021	AV	R,A
1005	Anem (Keraiai)	4181	1006	Anem (Purailing)	5191	AV	R,A
1005	Anem (Keraiai)	4191	1006	Anem (Purailing)	5083	AV	R,A
1005	Anem (Keraiai)	4191	1006	Anem (Purailing)	5101	AV	R,A
1007	Mangseng	12011	1013	Nakanai (Bileki)	15001	AV	R,A
1009	Mengen	14201	1012	Kol	8261	AV	R,A
1010	Sulka (Ganai)	20041	1011	Sulka (Watwat)	21021	AV	R,A
1010	Sulka (Ganai)	20171	1011	Sulka (Watwat)	21021	AV	R,A
1010	Sulka (Ganai)	20131	1027	Tigak	33241	AV	R,A
1014	Nakanai (Loso)	16161	1015	Mamusi (Kisiluvi)	10091	AV	R,A
1014	Nakanai (Loso)	16161	1015	Mamusi (Kisiluvi)	10161	AV	R,A
1014	Nakanai (Loso)	16131	1018	Ata (Lugei)	6211	AV	R,A
1015	Mamusi (Kisiluvi)	10041	1016	Mamusi (Lingite)	11081	AV	R,A
1015	Mamusi (Kisiluvi)	10071	1016	Mamusi (Lingite)	11031	AV	R,A
1015	Mamusi (Kisiluvi)	10071	1016	Mamusi (Lingite)	11071	AV	R,A
1015	Mamusi (Kisiluvi)	10071	1016	Mamusi (Lingite)	11131	AV	R,A
1015	Mamusi (Kisiluvi)	10181	1016	Mamusi (Lingite)	11231	AV	R,A
1015	Mamusi (Kisiluvi)	10191	1016	Mamusi (Lingite)	11021	AV	R,A
1015	Mamusi (Kisiluvi)	10201	1016	Mamusi (Lingite)	11175	AV	R,A
1015	Mamusi (Kisiluvi)	10231	1016	Mamusi (Lingite)	11043	AV	R,A
1018	Ata (Lugei)	6201	1026	Lavongai (South)	26051	AV	R,A
1019	Baining (Malasait)	17001	1021	Baining (Rangulit)	19141	AV	R,A
1019	Baining (Malasait)	17031	1021	Baining (Rangulit)	19181	AV	R,A
1019	Baining (Malasait)	17051	1021	Baining (Rangulit)	19071	AV	R,A
1019	Baining (Malasait)	17051	1021	Baining (Rangulit)	19201	AV	R,A
1019	Baining (Malasait)	17081	1021	Baining (Rangulit)	19171	AV	R,A
1019	Baining (Malasait)	17141	1021	Baining (Rangulit)	19071	AV	R,A

1019	Baining (Malasait)	17141	1021	Baining (Rangulit)	19201	AV	R,A
1019	Baining (Malasait)	17151	1021	Baining (Rangulit)	19201	AV	R,A
1019	Baining (Malasait)	17211	1021	Baining (Rangulit)	19131	AV	R,A
1019	Baining (Malasait)	17221	1021	Baining (Rangulit)	19051	AV	R,A
1019	Baining (Malasait)	17221	1021	Baining (Rangulit)	19181	AV	R,A
1019	Baining (Malasait)	17241	1021	Baining (Rangulit)	19181	AV	R,A
1022	Tolai (Kabakada)	22001	1023	Tolai (Vunairoto)	23171	AV	R,A
1022	Tolai (Kabakada)	22161	1023	Tolai (Vunairoto)	23201	AV	R,A
1022	Tolai (Kabakada)	22211	1023	Tolai (Vunairoto)	23152	AV	R,A
1024	Mussau	27051	1030	Kuot (Kabil)	28201	AV	R,A
1027	Tigak	33021	1029	Notsi	32223	AV	R,A
1027	Tigak	33051	1029	Notsi	32231	AV	R,A
1028	Nalik	31181	1029	Notsi	32021	AV	R,A
1028	Nalik	31121	1030	Kuot (Kabil)	28151	AV	R,A
1028	Nalik	31144	1030	Kuot (Kabil)	28151	AV	R,A
1029	Notsi	32211	1032	Madak	30091	AV	R,A
1005	Anem (Keraiai)	4161	1006	Anem (Purailing)	5061	GG	R,A
1005	Anem (Keraiai)	4181	1006	Anem (Purailing)	5131	GG	R,A
1015	Mamusi (Kisiluvi)	10173	1016	Mamusi (Lingite)	11151	GG	R,A
1026	Lavongai (South)	26081	1030	Kuot (Kabil)	28031	GG	R,A
1005	Anem (Keraiai)	4001	1006	Anem (Purailing)	5141	HS	R,A
1005	Anem (Keraiai)	4191	1006	Anem (Purailing)	5181	HS	R,A
1022	Tolai (Kabakada)	22191	1023	Tolai (Vunairoto)	23152	HS	R,A
1026	Lavongai (South)	26081	1030	Kuot (Kabil)	28021	HS	R,A
