# Supplementary material for: Population Structure in a Comprehensive Genomic Data Set on Human Microsatellite Variation
Source: G3 (Bethesda). 2013 May 1;3(5):891–907. doi: 10.1534/g3.113.005728 (PMC3656735; doi:10.1534/g3.113.005728)
Supplement: Supporting Information [file supp_g3.113.005728_TableS18.pdf]

Table S18 Three previously unreported inter-population parent/parent/offspring trios in the Pacific Islander data set

Parent 1			Parent 2			Offspring			Support for inference: RELPAIR (R) or allele-sharing (A)
Population		Identification number	Population		Identification number	Population		Identification number	
ID	Name		ID	Name		ID	Name		
1005	Anem (Keraiai)	4141	1006	Anem (Purailing)	5021	1005	Anem (Keraiai)	4021	R,A
1006	Anem (Purailing)	5101	1006	Anem (Purailing)	5181	1005	Anem (Keraiai)	4201	R,A
1022	Tolai (Kabakada)	22131	1023	Tolai (Vunairoto)	23213	1023	Tolai (Vunairoto)	23212	R,A
