# Supplementary material for: Population Structure in a Comprehensive Genomic Data Set on Human Microsatellite Variation
Source: G3 (Bethesda). 2013 May 1;3(5):891–907. doi: 10.1534/g3.113.005728 (PMC3656735; doi:10.1534/g3.113.005728)
Supplement: Supporting Information [file supp_g3.113.005728_TableS19.pdf]

Table S19 10 previously unreported inter-population second-degree relative pairs in the Native American data set

First individual			Second individual			RELPAIR inference: Avuncular (AV), grandparental (GG), or half-sibling (HS)	Support for inference: RELPAIR (R) or allele-sharing (A)
Population		Identification number	Population		Identification number		
ID	Name		ID	Name			
841	Kogi	2463	849	Arhuaco	2157	AV	R,A
841	Kogi	2463	849	Arhuaco	2158	AV	R,A
841	Kogi	2463	849	Arhuaco	2159	AV	R,A
841	Kogi	2463	849	Arhuaco	2160	AV	R,A
841	Kogi	2463	849	Arhuaco	2419	AV	R,A
841	Kogi	2463	849	Arhuaco	2420	AV	R,A
841	Kogi	2463	849	Arhuaco	2578	AV	R,A
841	Kogi	2463	849	Arhuaco	2732	AV	R,A
845	Ticuna (Arara)	2543	846	Ticuna (Tarapaca)	2794	AV	R,A
845	Ticuna (Arara)	2545	846	Ticuna (Tarapaca)	2794	AV	R,A
