# Supplementary material for: Population Structure in a Comprehensive Genomic Data Set on Human Microsatellite Variation
Source: G3 (Bethesda). 2013 May 1;3(5):891–907. doi: 10.1534/g3.113.005728 (PMC3656735; doi:10.1534/g3.113.005728)
Supplement: Supporting Information [file supp_g3.113.005728_TableS20.pdf]

Table S20 267 human populations present in the combined data set together with their geographic coordinates, sample sizes, and mean heterozygosities

Population		Location	Geographic region	Latitude [Degrees North]	Longitude [Degrees East]	Sample size			Unbiased heterozygosity		Source
ID	Name					MS5795	MS5547	MS5435	Mean	SD across loci	
20	Orcadian	Orkney Islands	Europe	59	-3	16	15	15	0.724	0.096	[4-6]
21	Adygei	Russia (Caucasus)	Europe	44	39	17	17	17	0.728	0.088	[4-6]
22	Russian	Russia	Europe	61	40	25	25	25	0.731	0.084	[4-6]
24	Basque	France	Europe	43	0	24	24	24	0.718	0.096	[4-6]
25	French	France	Europe	46	2	29	28	28	0.730	0.083	[4-6]
27	Italian	Italy (Bergamo)	Europe	46	10	13	13	13	0.727	0.095	[4-6]
28	Sardinian	Italy	Europe	40	9	28	28	28	0.723	0.086	[4-6]
29	Tuscan	Italy	Europe	43	11	8	8	8	0.735	0.109	[4-6]
34	Mozabite	Algeria (Mzab)	Middle East	32	3	30	29	29	0.738	0.082	[4-6]
36	Bedouin	Israel (Negev)	Middle East	31	35	48	47	46	0.734	0.078	[4-6]
37	Druze	Israel (Carmel)	Middle East	32	35	47	44	42	0.722	0.085	[4-6]
38	Palestinian	Israel (Central)	Middle East	32	35	51	50	46	0.732	0.080	[4-6]
50	Balochi	Pakistan	Central/South Asia	30.49871492	66.5	25	24	24	0.733	0.084	[4-6]
51	Brahui	Pakistan	Central/South Asia	30.49871492	66.5	25	25	25	0.730	0.087	[4-6]
52	Burusho	Pakistan	Central/South Asia	36.49838568	74	25	25	25	0.729	0.085	[4-6]
54	Hazara	Pakistan	Central/South Asia	33.49855601	70	23	22	21	0.728	0.093	[4-6]
56	Kalash	Pakistan	Central/South Asia	35.99366014	71.5	25	24	23	0.698	0.112	[4-6]
57	Makrani	Pakistan	Central/South Asia	26	64	24	24	24	0.737	0.086	[4-6]
58	Pathan	Pakistan	Central/South Asia	33.48700562	70.5	24	24	24	0.734	0.088	[4-6]
59	Sindhi	Pakistan	Central/South Asia	25.49063551	69	25	24	24	0.733	0.084	[4-6]
81[a]	Piapoco	Colombia	America	3	-68	13	7	7	0.625	0.162	[4-6]
82	Karitiana	Brazil	America	-10	-63	24	14	14	0.561	0.178	[4-6]
83	Surui	Brazil	America	-11	-62	21	9	8	0.507	0.203	[4-6]
86	Maya	Mexico	America	19	-91	25	22	21	0.676	0.122	[4-6]
87	Pima	Mexico	America	29	-108	25	14	14	0.617	0.161	[4-6]
430	Bantu (S. Africa)	Southern Africa	Africa	-25.56926433	24.25	8	8	8	0.765	0.103	[4-6]
441	Bantu (Kenya)	Kenya	Africa	-3	37	12	11	11	0.758	0.097	[4-6]
464	Mandenka	Senegal	Africa	12	-12	24	24	22	0.753	0.085	[4-6]
465	Yoruba	Nigeria	Africa	7.995094727	5	25	22	22	0.760	0.083	[4-6]
488	Biaka Pygmy	Central African Republic	Africa	4	17	32	27	23	0.758	0.084	[4-6]
489	Mbuti Pygmy	Congo	Africa	1	29	15	13	13	0.752	0.099	[4-6]
494	San	Namibia	Africa	-21	20	7	6	6	0.745	0.128	[4-6]

601	Han	China	East Asia	32.26566812	114	34	34	34	0.708	0.105	[4-6]
602	Han (N. China)	China	East Asia	32.26566812	114	10	10	10	0.714	0.117	[4-6]
606	Dai	China	East Asia	21	100	10	10	10	0.700	0.133	[4-6]
607	Daur	China	East Asia	48.49753416	124	10	10	10	0.711	0.119	[4-6]
608	Hezhen	China	East Asia	47.4976192	133.5	9	9	9	0.707	0.119	[4-6]
611	Lahu	China	East Asia	22	100	10	8	8	0.688	0.139	[4-6]
612	Miao	China	East Asia	28	109	10	10	10	0.700	0.127	[4-6]
613	Oroqen	China	East Asia	50.43389257	126.5	10	9	9	0.696	0.130	[4-6]
615	She	China	East Asia	27	119	10	10	10	0.694	0.122	[4-6]
616	Tujia	China	East Asia	29	109	10	10	10	0.702	0.127	[4-6]
617	Tu	China	East Asia	36	101	10	10	10	0.707	0.118	[4-6]
618	Xibo	China	East Asia	43.49792973	81.5	9	9	9	0.709	0.126	[4-6]
619	Yi	China	East Asia	28	103	10	10	10	0.709	0.124	[4-6]
622	Mongola	China	East Asia	45	111	10	10	10	0.711	0.122	[4-6]
625	Naxi	China	East Asia	26	100	10	9	9	0.699	0.122	[4-6]
629	Uygur	China	Central/South Asia	44	81	10	10	10	0.733	0.108	[4-6]
677	Cambodian	Cambodia	East Asia	12	105	11	10	10	0.711	0.127	[4-6]
684	Japanese	Japan	East Asia	38	138	29	29	29	0.702	0.113	[4-6]
699	Yakut	Siberia	East Asia	62.98287845	129.5	25	25	25	0.700	0.104	[4-6]
690	Tundra Nentsi	Siberia	East Asia	66.08	76.5	13	13	13	0.716	0.105	[7]
811	Chipewyan	Canada	America	59.55	-107.3	28	24	17	0.671	0.115	[7]
812	Cree	Canada	America	50.33	-102.5	17	16	16	0.695	0.116	[7]
813	Ojibwa	Canada	America	46.5	-81	20	16	12	0.696	0.115	[7]
821	Kaqchikel	Guatemala	America	15	-91	12	11	11	0.665	0.138	[7]
822	Mixtec	Mexico	America	17	-97	19	17	17	0.644	0.143	[7]
823	Mixe	Mexico	America	17	-96	20	20	20	0.641	0.134	[7]
824	Zapotec	Mexico	America	16	-97	17	17	17	0.665	0.140	[7]
831	Guaymi	Panama	America	8.5	-82	16	16	14	0.582	0.176	[7]
832	Cabecar	Costa Rica	America	9.5	-84	20	19	17	0.623	0.147	[7]
833	Aymara	Chile	America	-22	-70	18	18	18	0.660	0.132	[7]
834	Huilliche	Chile	America	-41	-73	19	19	19	0.667	0.120	[7]
835	Guarani	Brazil	America	-23	-54	10	8	8	0.649	0.146	[7]
836	Ache	Paraguay	America	-24	-56	17	16	14	0.483	0.207	[7]
837	Kaingang	Brazil	America	-24	-52.5	5	5	5	0.624	0.189	[7]
838	Quechua	Peru	America	-14	-74	20	20	20	0.669	0.124	[7]
841	Kogi	Colombia	America	11	-74	16	8	7	0.580	0.180	[7]

842	Zenu	Colombia	America	9	-75	18	16	13	0.643	0.143	[7]
843	Inga	Colombia	America	1	-77	16	15	14	0.640	0.140	[7]
844	Wayuu	Colombia	America	11	-73	17	15	15	0.671	0.126	[7]
845	Ticuna (Arara)	Colombia	America	-4	-70	15	14	14	0.590	0.163	[7]
846	Ticuna (Tarapaca)	Colombia	America	-4	-70	18	12	10	0.587	0.175	[7]
847	Embera	Colombia	America	7	-76	11	7	7	0.625	0.158	[7]
848	Waunana	Colombia	America	5	-77	20	12	11	0.625	0.160	[7]
849	Arhuaco	Colombia	America	11	-73.8	16	9	9	0.633	0.148	[7]
871	Oriente	Guatemala	Latino	14.633333	-90.516667	19	19	19	0.727	0.085	[8]
872	Mexico City	Mexico	Latino	19.4	-99.2	19	19	19	0.732	0.088	[8]
881	CVCR	Costa Rica	Latino	9.933333	-84.083333	20	20	20	0.736	0.084	[8]
882	Quetalmahue	Chile	Latino	-41.466667	-73.533333	20	20	19	0.703	0.103	[8]
883	Paposo	Chile	Latino	-25.05	-70.266667	20	19	18	0.710	0.099	[8]
884	Catamarca	Argentina	Latino	-28.466667	-65.783333	12	12	12	0.723	0.101	[8]
885	Salta	Argentina	Latino	-24.783333	-65.416667	19	19	18	0.703	0.107	[8]
886	Tucuman	Argentina	Latino	-26.816667	-65.216667	19	19	19	0.737	0.086	[8]
887	RGS	Brazil	Latino	-31	-54	20	20	20	0.744	0.076	[8]
891	Pasto	Colombia	Latino	1.216667	-77.283333	19	19	19	0.718	0.092	[8]
892	Peque	Colombia	Latino	7.316667	-75.833333	20	19	19	0.708	0.099	[8]
893	Medellin	Colombia	Latino	6.25	-75.583333	20	20	20	0.738	0.082	[8]
894	Cundinamarca	Colombia	Latino	4.6	-74.083333	19	19	19	0.721	0.096	[8]
901[b]	Ashkenazi Jewish	Israel (Ashkelon)	Europe	31.666667	34.566667	19	19	19	0.732	0.088	[9]
902[b]	Moroccan Jewish	Israel (Ashkelon)	Middle East	31.666667	34.566667	20	20	20	0.728	0.089	[9]
903[b]	Tunisian Jewish	Israel (Ashkelon)	Middle East	31.666667	34.566667	20	20	18	0.722	0.095	[9]
904[b]	Turkish Jewish	Israel (Ashkelon)	Middle East	31.666667	34.566667	20	20	20	0.735	0.084	[9]
501	Assamese	India	Central/South Asia	26	93	25	25	25	0.732	0.086	[10]
502	Bengali	India	Central/South Asia	23.25766454	87.66666667	27	27	27	0.732	0.087	[10]
504[c]	Gujarati	India	Central/South Asia	23	72	252	234	229	0.725	0.083	[10, 11]
505	Hindi	India	Central/South Asia	26.98091726	78.39357143	28	28	28	0.729	0.087	[10]
506	Kannada	India	Central/South Asia	15	75	24	24	24	0.731	0.088	[10]
507	Kashmiri	India	Central/South Asia	32.44	74.54	25	25	25	0.735	0.087	[10]
508	Konkani	India	Central/South Asia	14.57081522	75.13095238	42	42	42	0.726	0.083	[10]
509	Malayalam	India	Central/South Asia	10	76.25	25	25	25	0.729	0.091	[10]
510	Marathi	India	Central/South Asia	19.81684261	75.94230769	26	26	26	0.734	0.084	[10]
511	Marwari	India	Central/South Asia	26.67171131	74.264	25	25	25	0.725	0.092	[10]
512	Oriya	India	Central/South Asia	20	85	26	26	26	0.728	0.088	[10]

513	Parsi	India	Central/South Asia	19	72.8	25	25	25	0.724	0.090	[10]
514	Punjabi	India	Central/South Asia	30.47159766	75.29071429	27	27	27	0.732	0.085	[10]
515	Tamil	India	Central/South Asia	11.10167224	77.94827586	29	29	29	0.732	0.087	[10]
516	Telugu	India	Central/South Asia	15.9257581	79.62962963	27	27	27	0.727	0.091	[10]
1001[d]	East Highlands (Gimi & Goroka)	New Guinea	Oceania	-6.083	145.4	29	28	27	0.677	0.134	[2]
1003	Sepik	New Guinea	Oceania	-3.583	143.333	20	20	20	0.682	0.134	[2]
1004	Kove	New Britain	Oceania	-5.4667	148.95	25	24	20	0.677	0.125	[2]
1005	Anem (Keraiai)	New Britain	Oceania	-5.45	148.9833	16	14	11	0.684	0.128	[2]
1006	Anem (Purailing)	New Britain	Oceania	-5.45	148.984	17	14	14	0.674	0.136	[2]
1007	Mangseng	New Britain	Oceania	-5.933	150.7	20	16	15	0.697	0.121	[2]
1008	Melamela	New Britain	Oceania	-5	151.25	25	24	20	0.685	0.119	[2]
1009	Mengen	New Britain	Oceania	-5.1	151.4	23	20	20	0.688	0.129	[2]
1010	Sulka (Ganai)	New Britain	Oceania	-4.5	152.333	22	17	17	0.689	0.128	[2]
1011	Sulka (Watwat)	New Britain	Oceania	-4.483	152.3	15	15	14	0.686	0.136	[2]
1012	Kol	New Britain	Oceania	-5.383	151.633	20	18	16	0.672	0.135	[2]
1013	Nakanai (Bileki)	New Britain	Oceania	-5.75	150.8	24	24	20	0.686	0.123	[2]
1014	Nakanai (Loso)	New Britain	Oceania	-5.483	150.8	17	16	15	0.657	0.135	[2]
1015	Mamusi (Kisiluvi)	New Britain	Oceania	-5.7333	151.0833	22	20	19	0.655	0.138	[2]
1016	Mamusi (Lingite)	New Britain	Oceania	-5.867	151.1	20	16	16	0.646	0.148	[2]
1017	Ata (Uasilau)	New Britain	Oceania	-5.683	151	24	24	23	0.649	0.136	[2]
1018	Ata (Lugei)	New Britain	Oceania	-5.6	151	23	18	17	0.661	0.130	[2]
1019	Baining (Malasait)	New Britain	Oceania	-4.467	151.9	25	22	21	0.620	0.155	[2]
1020	Baining (Marabu)	New Britain	Oceania	-4.633	152.3	25	18	18	0.624	0.156	[2]
1021	Baining (Rangulit)	New Britain	Oceania	-4.4	151.9	22	19	18	0.628	0.151	[2]
1022	Tolai (Kabakada)	New Britain	Oceania	-4.483	152.1	18	17	16	0.684	0.132	[2]
1023	Tolai (Vunairoto)	New Britain	Oceania	-4.2	152.1	21	21	19	0.690	0.124	[2]
1024	Mussau	Mussau	Oceania	-1.583	149.733	25	22	22	0.691	0.121	[2]
1025	Lavongai (North)	New Hanover	Oceania	-2.433	150.35	24	21	21	0.686	0.122	[2]
1026	Lavongai (South)	New Hanover	Oceania	-2.5667	150.4333	22	19	17	0.688	0.126	[2]
1027	Tigak	New Ireland	Oceania	-2.6	150.87	23	23	22	0.691	0.120	[2]
1028	Nalik	New Ireland	Oceania	-2.983	151.52	24	19	18	0.693	0.122	[2]
1029	Notsi	New Ireland	Oceania	-3.05	151.65	23	19	19	0.690	0.126	[2]
1030	Kuot (Kabil)	New Ireland	Oceania	-3.067	151.7	25	22	20	0.684	0.124	[2]
1031	Kuot (Lamalaua)	New Ireland	Oceania	-3	151.5	18	17	16	0.690	0.128	[2]
1032	Madak	New Ireland	Oceania	-3.1	151.7	24	20	17	0.687	0.126	[2]

1033	Saposa	Bougainville	Oceania	-5.5833	154.67	25	24	20	0.685	0.126	[2]
1034	Teop	Bougainville	Oceania	-5.85	155.18	23	19	18	0.690	0.128	[2]
1035	Aita	Bougainville	Oceania	-5.9	155.083	25	19	17	0.643	0.143	[2]
1037[e]	Nasioi	Bougainville	Oceania	-6.483	155.833	23	14	12	0.667	0.147	[2]
1040	Micronesians	Micronesia	Oceania	12.5	150	16	16	16	0.693	0.123	[2]
1041	Samoans	Polynesia	Oceania	-13.35	-172.2	10	10	10	0.680	0.142	[2]
1042	Maoris	Polynesia	Oceania	-41	174	30	27	27	0.662	0.122	[2]
1043	Ami	Taiwan	East Asia	23.3	121	25	23	23	0.672	0.126	[2]
1044	Taruko	Taiwan	East Asia	23.3	121	25	24	23	0.653	0.142	[2]
1101	Hadza	Tanzania	Africa	-3.8	35.3	61	61	57	0.730	0.092	[3]
1102	Sandawe	Tanzania	Africa	-5.5	35.5	51	51	51	0.763	0.074	[3]
1103	Iraqw	Tanzania	Africa	-4	35.5	46	46	45	0.758	0.073	[3]
1104	Turu	Tanzania	Africa	-5	35	32	32	32	0.768	0.072	[3]
1105	Mbugwe	Tanzania	Africa	-3.8	35.8	21	21	21	0.765	0.082	[3]
1106	Rangi	Tanzania	Africa	-5	36	36	36	36	0.765	0.077	[3]
1107	Burunge	Tanzania	Africa	-5.3	36	21	21	21	0.767	0.082	[3]
1108	Maasai (Tanzania)	Tanzania	Africa	-4	37	36	36	36	0.767	0.072	[3]
1109	Akie	Tanzania	Africa	-5	37.5	23	23	23	0.756	0.087	[3]
1110	Pare	Tanzania	Africa	-4.5	38	23	23	22	0.762	0.082	[3]
1111	Mbugu	Tanzania	Africa	-4.8	38.5	22	22	22	0.750	0.084	[3]
1112	Baka	Cameroon	Africa	2.5	13.5	48	48	48	0.761	0.083	[3]
1113	Bakola	Cameroon	Africa	2.8	10	42	42	42	0.760	0.082	[3]
1114	Bedzan	Cameroon	Africa	5.5	11.6	17	16	15	0.756	0.094	[3]
1115	Mvae	Cameroon	Africa	3	12	24	24	24	0.764	0.081	[3]
1116	Ngumba	Cameroon	Africa	3	10.3	26	26	26	0.762	0.083	[3]
1117	Fulani (Adamawa)	Cameroon	Africa	9	13.5	41	41	41	0.755	0.078	[3]
1118	Kanuri	Cameroon	Africa	11.3	14.3	31	31	31	0.760	0.079	[3]
1119	Mada	Cameroon	Africa	10.8	14.1	28	28	28	0.754	0.082	[3]
1120	Zime	Cameroon	Africa	9	14.5	30	30	30	0.763	0.082	[3]
1121	Ouldeme	Cameroon	Africa	11	14.3	26	26	26	0.753	0.085	[3]
1122	Giziga	Cameroon	Africa	10.3	14.3	24	24	24	0.756	0.086	[3]
1123	Mandara	Cameroon	Africa	11.3	14	26	26	26	0.756	0.080	[3]
1124	Baggara	Cameroon	Africa	12.5	14.5	23	23	23	0.758	0.079	[3]
1125	Kotoko	Cameroon	Africa	11.8	14.8	17	17	17	0.756	0.086	[3]
1126	Zulgo	Cameroon	Africa	10.8	14	22	22	22	0.751	0.087	[3]
1127	Bamoun	Cameroon	Africa	5.5	10.8	31	31	30	0.760	0.081	[3]

1128	Banen	Cameroon	Africa	4.8	10.8	25	25	25	0.760	0.084	[3]
1129	Bafia	Cameroon	Africa	4.8	11	30	30	30	0.762	0.082	[3]
1130	Lemande	Cameroon	Africa	4.5	11	26	26	26	0.758	0.083	[3]
1131	Batanga	Cameroon	Africa	3	10	20	20	20	0.757	0.089	[3]
1132	Podokwo	Cameroon	Africa	11	12.1	30	30	29	0.751	0.084	[3]
1159	Yoruba	Nigeria	Africa	8	4	25	25	25	0.756	0.086	[3]
1185	Bassange	Nigeria	Africa	9	5.5	20	20	20	0.754	0.090	[3]
1186	Igbo	Nigeria	Africa	6	7	28	28	28	0.760	0.084	[3]
1187	Igala	Nigeria	Africa	7	7	17	17	17	0.758	0.084	[3]
1188	Gwari	Nigeria	Africa	10	7	22	22	22	0.758	0.086	[3]
1189	Hausa (Nigeria)	Nigeria	Africa	12	8	16	16	16	0.755	0.093	[3]
1190	Beja (Banuamir)	Sudan	Middle East	21	36	23	23	23	0.752	0.080	[3]
1191	Dinka	Sudan	Africa	8	30	16	16	16	0.750	0.090	[3]
1192	Datoga	Tanzania	Africa	-4.5	35.5	54	54	54	0.760	0.072	[3]
1193	Sukuma	Tanzania	Africa	-3	33.5	10	10	10	0.766	0.090	[3]
1194	Gogo	Tanzania	Africa	-6	36	13	13	13	0.767	0.084	[3]
1195	Fiome (Gorowa)	Tanzania	Africa	-4.3	35.8	22	22	22	0.762	0.081	[3]
1196	Iyassa	Cameroon	Africa	2.5	9.8	37	37	37	0.761	0.081	[3]
1197	Fang	Cameroon	Africa	2.5	13	19	19	19	0.759	0.087	[3]
1198	Mabea	Cameroon	Africa	2.9	10.3	13	13	13	0.761	0.090	[3]
1199	Yambassa	Cameroon	Africa	4.8	11.3	17	17	17	0.758	0.090	[3]
1200	Tikar (South)	Cameroon	Africa	5.5	11.5	21	21	20	0.763	0.085	[3]
1201	Tikar (North)	Cameroon	Africa	6.3	11.5	13	13	13	0.759	0.092	[3]
1202	Ntumu	Cameroon	Africa	2.3	10.5	11	11	11	0.762	0.091	[3]
1203	Massa	Cameroon	Africa	10.3	15.3	15	15	15	0.754	0.093	[3]
1204	Tupuri	Cameroon	Africa	10.3	14.8	22	22	22	0.757	0.086	[3]
1205	Bulu	Cameroon	Africa	3	11	22	22	22	0.765	0.081	[3]
1206	Ashanti	Ghana	Africa	6	-1	15	15	15	0.759	0.092	[3]
1207	Brong	Ghana	Africa	7.5	-2	26	26	26	0.757	0.084	[3]
1209[b]	Chicago	USA	Afro-European	41.881944	-87.627778	15	15	15	0.766	0.082	[3]
1210[b]	Pittsburgh	USA	Afro-European	40.441667	-80	21	21	21	0.766	0.079	[3]
1211[b]	Baltimore	USA	Afro-European	39.283333	-76.616667	44	44	44	0.767	0.073	[3]
1212	North Carolina	USA	Afro-European	-	-	18	18	18	0.768	0.080	[3]
1214	Maasai (Mumonyot)	Kenya	Africa	0.6	37	12	12	12	0.759	0.083	[3]
1215	Maasai (Ilgwesi)	Kenya	Africa	0.3	36.8	21	21	21	0.758	0.082	[3]
1216	Samburu	Kenya	Africa	1.5	37	18	18	18	0.763	0.082	[3]

1217	Yaaku	Kenya	Africa	0.5	37	19	19	19	0.750	0.082	[3]
1219	Sambaa	Tanzania	Africa	-4.5	38.3	18	18	18	0.760	0.085	[3]
1220	Dorobo	Tanzania	Africa	-5	37	10	10	10	0.759	0.097	[3]
1222	Australian	Australia	Oceania	-	-	10	9	9	0.719	0.121	[3]
1223	Beja (Hadandawa)	Sudan	Middle East	21	36	19	19	19	0.745	0.084	[3]
1224	Tugen	Kenya	Africa	0.8	35.8	22	22	22	0.757	0.077	[3]
1225	Burji	Ethiopia	Africa	5.5	37.8	24	24	24	0.763	0.076	[3]
1226	Marakwet	Kenya	Africa	1.3	35.5	14	14	14	0.754	0.094	[3]
1227	Luhya	Kenya	Africa	0.5	34.5	17	17	17	0.763	0.086	[3]
1228	Luo	Kenya	Africa	-0.5	34.5	28	28	28	0.763	0.082	[3]
1229	Kikuyu	Kenya	Africa	-1	37	21	21	21	0.764	0.080	[3]
1230	Sengwer	Kenya	Africa	1	35	21	21	21	0.754	0.079	[3]
1231	Okiek	Kenya	Africa	0.3	36	22	22	22	0.746	0.085	[3]
1232	Wata	Kenya	Africa	3.5	37	6	6	6	0.762	0.105	[3]
1233	Nandi	Kenya	Africa	0	35.5	11	11	11	0.761	0.094	[3]
1234	El Molo	Kenya	Africa	2.8	36.8	16	16	16	0.749	0.088	[3]
1235	Gabra	Kenya	Africa	3	37.5	16	16	16	0.755	0.083	[3]
1236	Sabaot	Kenya	Africa	1	34.8	20	20	20	0.762	0.079	[3]
1237	Rendille	Kenya	Africa	2.3	37.5	28	28	28	0.755	0.075	[3]
1238	Turkana	Kenya	Africa	3	36	26	26	26	0.760	0.081	[3]
1239	Pokot	Kenya	Africa	1.5	35.5	23	23	22	0.755	0.082	[3]
1240	Borana	Kenya	Africa	3	38	32	32	32	0.760	0.078	[3]
1241	Maasai (Ilchamus)	Kenya	Africa	1.5	37.1	26	26	26	0.759	0.079	[3]
1242	Konso	Ethiopia	Africa	5.5	37.5	14	14	14	0.762	0.090	[3]
1243	Laka	Chad	Africa	8	16	33	33	33	0.760	0.084	[3]
1244	Ngambaye	Chad	Africa	9	16	30	30	30	0.758	0.082	[3]
1245	Kaba	Chad	Africa	8	16.8	27	27	27	0.760	0.080	[3]
1246	Bulala	Chad	Africa	13	18	15	15	15	0.754	0.089	[3]
1247	Kanembou	Chad	Africa	14	15	5	5	5	0.756	0.119	[3]
1248	Mbum	Central African Republic	Africa	5.5	13.5	13	13	13	0.755	0.099	[3]
1249	Tutsi/Hutu	Rwanda	Africa	-2	30	8	8	8	0.764	0.097	[3]
1250	Yakoma	Central African Republic	Africa	4.3	22.3	6	6	6	0.759	0.121	[3]
1252	Gbaya	Central African Republic	Africa	5	15	15	15	15	0.760	0.085	[3]
1253	Fulani (Mbororo)	Cameroon	Africa	11.8	14.8	13	13	13	0.737	0.093	[3]
1254	Wimbum	Cameroon	Africa	6.5	10.8	14	14	14	0.756	0.095	[3]
1255	Batie	Cameroon	Africa	4.3	11	16	16	16	0.760	0.093	[3]

1256	Hausa (Cameroon)	Cameroon	Africa	10.5	14.5	27	27	27	0.760	0.083	[3]
1257	Kongo	Democratic Republic Congo	Africa	-5.5	15	17	17	17	0.759	0.088	[3]
1258	Barega	Democratic Republic Congo	Africa	-3.5	28	4	4	4	0.763	0.135	[3]
1259	Baluba	Democratic Republic Congo	Africa	-9	25	5	5	5	0.757	0.130	[3]
1262	Dioula	Ivory Coast	Africa	9.5	-4.5	5	5	5	0.756	0.119	[3]
1266	Ewondo	Cameroon	Africa	3.8	11.5	3	3	3	0.760	0.163	[3]
1267	Eton	Cameroon	Africa	4.3	11.5	4	4	4	0.764	0.130	[3]
1268	Nuer	Sudan	Africa	8.5	31	17	17	17	0.750	0.087	[3]
1269	Shilook	Sudan	Africa	10	32	15	15	15	0.751	0.094	[3]
1270	Nyimang	Sudan	Africa	12.3	29.5	12	12	12	0.750	0.095	[3]
1271	Sara (various)	Chad	Africa	8	17.5	26	26	26	0.759	0.084	[3]
1272	Cape Mixed Ancestry	South Africa (Cape Town)	Afro-European	-34	18.5	33	33	33	0.767	0.074	[3]
1273	Venda	South Africa	Africa	-22.5	30	11	11	11	0.752	0.096	[3]
1274	!Xun/Kxoe	South Africa	Africa	-30	18	6	6	6	0.757	0.129	[3]
1275	Xhosa	South Africa	Africa	-32	28	27	27	27	0.762	0.086	[3]
1276	Koma	Nigeria	Africa	8.5	12.7	9	9	9	0.743	0.113	[3]
1277	Beta Israel	Ethiopia	Africa	12	38	17	17	17	0.753	0.081	[3]
1278	Dogon	Mali	Africa	14	-3	3	3	3	0.371	0.305	[3]
1280	Temani	Yemen	Middle East	15	45	18	18	18	0.724	0.093	[3]
1281	Fulani (Nigeria)	Nigeria	Africa	11	11	4	4	4	0.757	0.138	[3]

[a] Referred to as 'Colombian' in Rosenberg *et al*. [6], Rosenberg *et al*. [5], and Ramachandran *et al*. [4].

[b] The geographic coordinates of the city where sampling took place are provided.

[c] This population includes the Gujarati individuals studied by Pemberton *et al*. [11].

[d] This population includes the individuals from the HGDP-CEPH Papuan population.

[e] This population includes the individuals from the HGDP-CEPH Melanesian population.
