# Supplementary material for: Population Structure in a Comprehensive Genomic Data Set on Human Microsatellite Variation
Source: G3 (Bethesda). 2013 May 1;3(5):891–907. doi: 10.1534/g3.113.005728 (PMC3656735; doi:10.1534/g3.113.005728)
Supplement: Supporting Information [file supp_g3.113.005728_TableS21.pdf]

Table S21 The 248 individuals in MS5795 but not in MS5547

Population		Identification number	Population		Identification number
ID	Name		ID	Name	
20	Orcadian	801	87	Pima	1049
25	French	532	87	Pima	1052
34	Mozabite	1281	87	Pima	1054
36	Bedouin	633	87	Pima	1061
37	Druze	592	441	Bantu (Kenya)	1413
37	Druze	603	465	Yoruba	921
37	Druze	605	465	Yoruba	922
38	Palestinian	695	465	Yoruba	923
50	Balochi	84	488	Biaka Pygmy	451
54	Hazara	113	488	Biaka Pygmy	477
56	Kalash	292	488	Biaka Pygmy	1088
59	Sindhi	203	488	Biaka Pygmy	1089
81	Piapoco	705	488	Biaka Pygmy	1093
81	Piapoco	707	489	Mbuti Pygmy	468
81	Piapoco	709	489	Mbuti Pygmy	983
81	Piapoco	792	494	San	988
81	Piapoco	793	504	Gujarati	4250001
81	Piapoco	827	504	Gujarati	46500080
82	Karitiana	996	504	Gujarati	48900024
82	Karitiana	997	504	Gujarati	48900027
82	Karitiana	1000	504	Gujarati	425000471
82	Karitiana	1004	504	Gujarati	425000474
82	Karitiana	1005	504	Gujarati	425000475
82	Karitiana	1007	504	Gujarati	425000476
82	Karitiana	1008	504	Gujarati	425000477
82	Karitiana	1011	504	Gujarati	425000478
82	Karitiana	1016	504	Gujarati	425000480
82	Karitiana	1017	504	Gujarati	425000481
83	Surui	830	504	Gujarati	425000482
83	Surui	834	504	Gujarati	425000485
83	Surui	835	504	Gujarati	425000489
83	Surui	839	504	Gujarati	425000501
83	Surui	840	504	Gujarati	425000502
83	Surui	841	504	Gujarati	506000139
83	Surui	842	611	Lahu	1324
83	Surui	844	611	Lahu	1325
83	Surui	847	613	Oroqen	1210
83	Surui	848	625	Naxi	1343
83	Surui	850	677	Cambodian	718
83	Surui	851	811	Chipewyan	2395
86	Maya	866	811	Chipewyan	2399
86	Maya	867	811	Chipewyan	2400
86	Maya	878	811	Chipewyan	2560
87	Pima	1038	812	Cree	2418
87	Pima	1039	813	Ojibwa	2421
87	Pima	1040	813	Ojibwa	2430
87	Pima	1042	813	Ojibwa	2432
87	Pima	1045	813	Ojibwa	2440
87	Pima	1046	821	Kaqchikel	2690

87	Pima	1048	822	Mixtec	2054
822	Mixtec	2055	1007	Mangseng	12034
832	Cabecar	2034	1007	Mangseng	12041
835	Guarani	2726	1007	Mangseng	12131
835	Guarani	2729	1008	Melamela	13111
836	Ache	2746	1009	Mengen	14021
841	Kogi	2461	1009	Mengen	14081
841	Kogi	2462	1009	Mengen	14111
841	Kogi	2463	1010	Sulka (Ganai)	20001
841	Kogi	2464	1010	Sulka (Ganai)	20161
841	Kogi	2467	1010	Sulka (Ganai)	20171
841	Kogi	2473	1010	Sulka (Ganai)	20201
841	Kogi	2475	1010	Sulka (Ganai)	20241
841	Kogi	2476	1012	Kol	8021
842	Zenu	2485	1012	Kol	8051
842	Zenu	2487	1014	Nakanai (Loso)	16001
843	Inga	2518	1015	Mamusi (Kisiluvi)	10161
844	Wayuu	2525	1015	Mamusi (Kisiluvi)	10241
844	Wayuu	2539	1016	Mamusi (Lingite)	11011
845	Ticuna (Arara)	2543	1016	Mamusi (Lingite)	11051
846	Ticuna (Tarapaca)	2764	1016	Mamusi (Lingite)	11121
846	Ticuna (Tarapaca)	2767	1016	Mamusi (Lingite)	11191
846	Ticuna (Tarapaca)	2768	1018	Ata (Lugei)	6003
846	Ticuna (Tarapaca)	2793	1018	Ata (Lugei)	6101
846	Ticuna (Tarapaca)	2795	1018	Ata (Lugei)	6123
846	Ticuna (Tarapaca)	2797	1018	Ata (Lugei)	6161
847	Embera	2562	1018	Ata (Lugei)	6181
847	Embera	2564	1019	Baining (Malasait)	17051
847	Embera	2566	1019	Baining (Malasait)	17081
847	Embera	2569	1019	Baining (Malasait)	17211
848	Waunana	2584	1020	Baining (Marabu)	18001
848	Waunana	2585	1020	Baining (Marabu)	18021
848	Waunana	2586	1020	Baining (Marabu)	18131
848	Waunana	2587	1020	Baining (Marabu)	18171
848	Waunana	2588	1020	Baining (Marabu)	18191
848	Waunana	2590	1020	Baining (Marabu)	18221
848	Waunana	2597	1020	Baining (Marabu)	18241
848	Waunana	2600	1021	Baining (Rangulit)	19071
849	Arhuaco	2158	1021	Baining (Rangulit)	19081
849	Arhuaco	2159	1021	Baining (Rangulit)	19101
849	Arhuaco	2458	1022	Tolai (Kabakada)	22191
849	Arhuaco	2460	1024	Mussau	27081
849	Arhuaco	2576	1024	Mussau	27181
849	Arhuaco	2577	1024	Mussau	27201
849	Arhuaco	2579	1025	Lavongai (North)	25001
883	Paposo	2266	1025	Lavongai (North)	25121
892	Peque	2623	1025	Lavongai (North)	25181
1001	East Highlands	54071	1026	Lavongai (North)	26141
1004	Kove	9201	1026	Lavongai (North)	26171
1005	Anem (Keraiai)	4051	1026	Lavongai (North)	26231
1005	Anem (Keraiai)	4101	1028	Nalik	31031
1006	Anem (Purailing)	5041	1028	Nalik	31071
1006	Anem (Purailing)	5053	1028	Nalik	31101
1006	Anem (Purailing)	5151	1028	Nalik	31144

1007	Mangseng	12021	1028	Nalik	31161
1029	Notsi	32061	1035	Aita	36121
1029	Notsi	32091	1035	Aita	36151
1029	Notsi	32171	1035	Aita	36231
1029	Notsi	32181	1037	Nasioi	490
1030	Kuot (Kabil)	28021	1037	Nasioi	657
1030	Kuot (Kabil)	28091	1037	Nasioi	658
1030	Kuot (Kabil)	28101	1037	Nasioi	662
1031	Kuot (Lamalaua)	29001	1037	Nasioi	663
1032	Madak	30081	1037	Nasioi	664
1032	Madak	30161	1037	Nasioi	789
1032	Madak	30171	1037	Nasioi	824
1032	Madak	30221	1037	Nasioi	825
1033	Saposa	34021	1042	Maoris	62171
1034	Teop	35001	1042	Maoris	62181
1034	Teop	35201	1042	Maoris	62221
1034	Teop	35211	1043	Ami	47191
1034	Teop	35221	1043	Ami	47221
1035	Aita	36003	1044	Taruko	42141
1035	Aita	36011	1114	Bedzan	71584
1035	Aita	36031	1222	Australian	79195
