# Supplementary material for: Population Structure in a Comprehensive Genomic Data Set on Human Microsatellite Variation
Source: G3 (Bethesda). 2013 May 1;3(5):891–907. doi: 10.1534/g3.113.005728 (PMC3656735; doi:10.1534/g3.113.005728)
Supplement: Supporting Information [file supp_g3.113.005728_TableS22.pdf]

Table S22 The 112 individuals in MS5547 but not in MS5435

Population		Identification number
ID	Name	
36	Bedouin	617
37	Druze	570
37	Druze	585
38	Palestinian	681
38	Palestinian	728
38	Palestinian	742
38	Palestinian	743
54	Hazara	128
56	Kalash	321
83	Surui	833
86	Maya	874
464	Mandenka	916
464	Mandenka	919
488	Biaka Pygmy	448
488	Biaka Pygmy	1084
488	Biaka Pygmy	1085
488	Biaka Pygmy	1091
504	Gujarati	42600087
504	Gujarati	50200040
504	Gujarati	51300078
504	Gujarati	404000112
504	Gujarati	503000106
811	Chipewyan	2156
811	Chipewyan	2383
811	Chipewyan	2387
811	Chipewyan	2390
811	Chipewyan	2393
811	Chipewyan	2515
811	Chipewyan	2800
813	Ojibwa	2428
813	Ojibwa	2436
813	Ojibwa	2437
813	Ojibwa	2438
831	Guaymi	2002
831	Guaymi	2014
832	Cabecar	2026
832	Cabecar	2037
836	Ache	2747
836	Ache	2748
841	Kogi	2466
842	Zenu	2494
842	Zenu	2496
842	Zenu	2499
843	Inga	2505
846	Ticuna (Tarapaca)	2761
846	Ticuna (Tarapaca)	2792
848	Waunana	2599
882	Quetalmahue	2288
883	Paposo	2267

Population		Identification number
ID	Name	
903	Tunisian Jewish	2345
903	Tunisian Jewish	2348
1001	East Highlands	54011
1004	Kove	9091
1004	Kove	9111
1004	Kove	9133
1004	Kove	9151
1005	Anem (Keraiai)	4071
1005	Anem (Keraiai)	4091
1005	Anem (Keraiai)	4141
1007	Mangseng	12071
1008	Melamela	13001
1008	Melamela	13091
1008	Melamela	13131
1008	Melamela	13141
1011	Sulka (Watwat)	21031
1012	Kol	8081
1012	Kol	8171
1013	Nakanai (Bileki)	15063
1013	Nakanai (Bileki)	15121
1013	Nakanai (Bileki)	15151
1013	Nakanai (Bileki)	15204
1014	Nakanai (Loso)	16023
1015	Mamusi (Kisiluvi)	10121
1017	Ata (Uasilau)	7181
1018	Ata (Lugei)	6081
1019	Baining (Malasait)	17201
1021	Baining (Rangulit)	19001
1022	Tolai (Kabakada)	22051
1023	Tolai (Vunairoto)	23131
1023	Tolai (Vunairoto)	23143
1026	Lavongai (South)	26091
1026	Lavongai (South)	26211
1027	Tigak	33081
1028	Nalik	31211
1030	Kuot (Kabil)	28081
1030	Kuot (Kabil)	28171
1031	Kuot (Lamalaua)	29131
1032	Madak	30072
1032	Madak	30104
1032	Madak	30111
1033	Saposa	34011
1033	Saposa	34151
1033	Saposa	34171
1033	Saposa	34231
1034	Teop	35043
1035	Aita	36091
1035	Aita	36211
1037	Nasioi	823

885	Salta	2180	1037	Nasioi	978
1044	Taruko	42151	1110	Pare	70446
1101	Hadza	70027	1114	Bedzan	71578
1101	Hadza	70047	1127	Bamoun	71054
1101	Hadza	70048	1132	Podokwo	73033
1101	Hadza	71474	1200	Tikar (South)	71731
1103	Iraqw	70204	1239	Pokot	72636
