# Supplementary material for: Population Structure in a Comprehensive Genomic Data Set on Human Microsatellite Variation
Source: G3 (Bethesda). 2013 May 1;3(5):891–907. doi: 10.1534/g3.113.005728 (PMC3656735; doi:10.1534/g3.113.005728)
Supplement: Supporting Information [file supp_g3.113.005728_TableS23.pdf]

Table S23 Six chimpanzee populations present in the chimpanzee data set together with their sample sizes and average heterozygosities in the combined human-chimpanzee data set

Population		Reported category in Becquet *et al.*	Sample size			Unbiased heterozygosity		Source
**ID**	**Species**		**MS5879**	**MS5631**	**MS5519**	**Mean**	**SD across loci**	
1	Chimpanzee	Central	16	16	16	0.738	0.212	[12]
2	Chimpanzee	Eastern	7	7	7	0.698	0.229	[12]
3	Chimpanzee	Western	41	41	41	0.627	0.239	[12]
4	Chimpanzee	Unreported	11	11	11	0.644	0.245	[12]
5	Chimpanzee	Hybrid	3	3	3	0.746	0.238	[12]
6	Bonobo	Bonobo	6	6	6	0.656	0.271	[12]

Population IDs are the same as those used in Becquet *et al*. [12].
