# Supplementary material for: Population Structure in a Comprehensive Genomic Data Set on Human Microsatellite Variation
Source: G3 (Bethesda). 2013 May 1;3(5):891–907. doi: 10.1534/g3.113.005728 (PMC3656735; doi:10.1534/g3.113.005728)
Supplement: Supporting Information [file supp_g3.113.005728_TableS24.pdf]

Table S24 Three individuals with >27.5% missing data in the combined human-chimpanzee data set

Population			Identification number of individual	Fraction of loci with missing genotypes
ID	Name	Data set of origin		
1270	Nyimang	African	103351	0.382
886	Tucuman	Latino	2194	0.317
1014	Nakanai (Loso)	Pacific Islander	16024	0.297
