# Supplementary material for: Population Structure in a Comprehensive Genomic Data Set on Human Microsatellite Variation
Source: G3 (Bethesda). 2013 May 1;3(5):891–907. doi: 10.1534/g3.113.005728 (PMC3656735; doi:10.1534/g3.113.005728)
Supplement: Supporting Information [file supp_g3.113.005728_TableS25.pdf]

Table S25 27 loci from the combined human data set of 645 loci with no genotype data in at least one population

ID in combined data set	Populations with no genotype data
GATA35_11	Cape Mixed Ancestry, Venda, !Xun/Kxoe, Xhosa, Koma, Beta Israel, Dogon, Temani
GATA11B08P_6	Koma, Beta Israel, Dogon, Temani
TCTA017M_9	Koma, Beta Israel, Dogon, Temani
AGAT139P_20	Koma, Beta Israel, Dogon, Temani
TTTA063P_1	Dogon, Fulani (Nigeria)
TTAT027P_15	Nuer, Shilook
D1S1653	Dogon
GATA51H01_1	Dogon
AGAT117_2	Dogon
D3S3045	Dogon
GATA138B05_5	Australian
GATA142H05P_5	Fulani (Nigeria)
D6S1027	Dogon
GATA61G06_7	Dogon
AGAT049P_7	Nuer
GATA65D11_9	Fulani (Nigeria)
D10S1208	Ewondo
ATA20B07_10	Dogon
ATA44G07M_10	Dogon
D11S4459	Nuer
AGAT110P_13	Shilook
GATA137B09_13	Fulani (Nigeria)
ATA70B03P_14	Nuer
D16S753	Dogon
D17S1294	Dogon
D18S858	Dogon
D19S1034	!Xun\Kxoe
