# Supplementary material for: Population Structure in a Comprehensive Genomic Data Set on Human Microsatellite Variation
Source: G3 (Bethesda). 2013 May 1;3(5):891–907. doi: 10.1534/g3.113.005728 (PMC3656735; doi:10.1534/g3.113.005728)
Supplement: Supporting Information [file supp_g3.113.005728_TableS26.pdf]

Table S26 10 loci from the combined human-chimpanzee data set of 246 loci with no genotype data in at least one population

ID in combined data set	Populations with no genotype data
TCTA017M_9	Koma, Beta Israel, Dogon, Temani
D1S1653	Dogon
GATA138B05_5	Australian
D6S1027	Dogon
GATA61G06_7	Dogon
D11S4459	Nuer
D12S1042	Bonobo
ATA70B03P_14	Nuer
D17S1294	Dogon
D18S858	Dogon
