# Supplementary material for: Population Structure in a Comprehensive Genomic Data Set on Human Microsatellite Variation
Source: G3 (Bethesda). 2013 May 1;3(5):891–907. doi: 10.1534/g3.113.005728 (PMC3656735; doi:10.1534/g3.113.005728)
Supplement: Supporting Information [file supp_g3.113.005728_SupportingReferences.pdf]

References

1. Rosenberg N. A., 2006 Standardized subsets of the HGDP-CEPH Human Genome Diversity Cell Line Panel, accounting for atypical and duplicated samples and pairs of close relatives. Ann. Hum. Genet. 70: 841-847.

2. Friedlaender J. S., F. R. Friedlaender , F. A. Reed, K. K. Kidd, J. R. Kidd, G. K. Chambers, R. A. Lea, J. H. Loo, G. Koki, J. A. Hodgson, *et al*., 2008 The genetic structure of Pacific Islanders. PLoS Genet. 4: e19.

3. Tishkoff S. A., F. A. Reed, F. R. Friedlaender, C. Ehret, A. Ranciaro, A. Froment, J. B. Hirbo, A. A. Awomoyi, J. M. Bodo, O. Doumbo, *et al*., 2009 The genetic structure and history of Africans and African Americans. Science 324: 1035-1044.

4. Ramachandran S., O. Deshpande, C. C. Roseman, N. A. Rosenberg, M. W. Feldman, and L. L. Cavalli-Sforza, 2005 Support from the relationship of genetic and geographic distance in human populations for a serial founder effect originating in Africa. Proc. Natl. Acad. Sci. U S A 102: 15942-15947.

5. Rosenberg N. A., S. Mahajan, S. Ramachandran, C. Zhao, J. K. Pritchard, and M. W. Feldman, 2005 Clines, clusters, and the effect of study design on the inference of human population structure. PLoS Genet. 1: e70.

6. Rosenberg N. A., J. K. Pritchard, J. L. Weber, H. M. Cann, K. K. Kidd, L. A. Zhivotovsky, and M. W. Feldman, 2002 Genetic structure of human populations. Science 298: 2381-2385.

7. Wang S., C. M. Lewis, M. Jakobsson, S. Ramachandran, N. Ray, G. Bedoya, W. Rojas, M. V. Parra, J. A. Molina, C. Gallo, *et al*., 2007 Genetic variation and population structure in Native Americans. PLoS Genet. 3: e185.

8. Wang S., N. Ray, W. Rojas, M. V. Parra, G. Bedoya, C. Gallo, G. Poletti, G. Mazzotti, K. Hill, A. M. Hurtado, *et al*., 2008 Geographic patterns of genome admixture in Latin American Mestizos. PLoS Genet. 4: e1000037.

9. Kopelman N. M., L. Stone, C. Wang, D. Gefel, M. W. Feldman, J. Hillel, and N. A. Rosenberg, 2009 Genomic microsatellites identify shared Jewish ancestry intermediate between Middle Eastern and European populations. BMC Genet. 10: 80.

10. Rosenberg N. A., S. Mahajan, C. Gonzalez-Quevedo, M. G. Blum, L. Nino-Rosales, V. Ninis, P. Das, M. Hegde, L. Molinari, G. Zapata, *et al*., 2006 Low levels of genetic divergence across geographically and linguistically diverse populations from India. PLoS Genet. 2: e215.

11. Pemberton T. J., F-Y. Li, E. K. Hanson, N. U. Mehta, S. Choi, J. Ballantyne, J. W. Belmont, N. A. Rosenberg, C. Tyler-Smith, and P. I. Patel, 2012 Impact of restricted marital practices on genetic variation in an endogamous Gujarati group. Am. J. Phys. Anthropol. 149: 92-103.

12. Becquet C., N. Patterson, A. C. Stone, M. Przeworski, and D. Reich, 2007 Genetic structure of chimpanzee populations. PLoS Genet. 3: e66.
